# Adaptive Riemannian optimization for multi-scale diffeomorphic matching

**DOI:** 10.1038/s41467-026-72508-3

**Published:** 2026-06-09

**Authors:** Rohit Jena, Pratik Chaudhari, James C. Gee

**Affiliations:** 1 Computer and Information Science, University of Pennsylvania, Philadelphia, PA USA; 2 Penn Image Computing and Science Laboratory, University of Pennsylvania, Philadelphia, PA USA; 3 Electrical and Systems Engineering, University of Pennsylvania, Philadelphia, PA USA; 4 Radiology, Perelman School of Medicine, University of Pennsylvania, Philadelphia, PA USA

**Keywords:** Computer science, Brain imaging, Radiography

## Abstract

Image matching is a fundamental task in quantitative biomedical and biological image analyses, enabling researchers to compare, integrate, and interpret imaging data across subjects, time points, modalities, and experimental conditions. Existing state-of-the-art registration methods are slow due to inefficient implementations and poor convergence rates because of the ill-conditioned nature of the optimization problem. Deep learning methods offer fast inference but require extensive training time, substantial inference memory, and fail to generalize across long-tailed distributions or diverse image modalities, necessitating costly retraining. We address these challenges by proposing FireANTs, a training-free, GPU-accelerated, multi-scale adaptive Riemannian optimization algorithm for fast and accurate dense diffeomorphic image matching. FireANTs more than doubles the speed of the community standard ANTs registration tool on a CPU, and is two orders of magnitude faster on a GPU. On the GPU, FireANTs performs competitively with deep learning methods on inference runtime while consuming up to 10 × less memory. FireANTs demonstrates robustness on a wide variety of matching problems across modalities, species, and organs, without any domain-specific training or tuning. Our framework allows hyperparameter grid search studies with less resources and time compared to traditional and deep learning registration algorithms alike.

## Abstract (web summary)

Large-scale image alignment of modern high-resolution biomedical and biological datasets demands higher-order algorithms that exploit GPU hardware and converge quickly. Here the authors present FireANTs, a scalable diffeomorphic registration framework achieving orders-of-magnitude speedups, enabling reproducible alignment of data across scales, modalities, species, and organs into common coordinate systems.

## Introduction

The ability to identify and map corresponding elements across diverse datasets or perceptual inputs—known as *correspondence matching*—is fundamental to interpreting and interacting with the world. Correspondence matching between images is one of the longstanding fundamental problems in computer vision. Influential computer vision researcher Takeo Kanade famously once said that the three fundamental problems of computer vision are: “Correspondence, correspondence, correspondence”^1^. Indeed, correspondence matching is fundamental and ubiquitous across various disciplines, manifesting in many forms including but not limited to stereo matching^2^, structure from motion^3,4^, template matching^5^, motion tracking^6,7^, shape correspondence^8^, semantic correspondence^9^, point cloud matching^10^, optical flow^11^, and deformable image matching^12^. Solving these problems addresses the desiderata for a wide range of applications in computer vision, robotics, medical imaging, remote sensing, photogrammetry, geological and ecological sciences, cognitive sciences, human-computer interaction, and self-driving, among many other fields.

Correspondence matching is broadly divided into two categories: sparse and dense matching. Most sparse matching problems, like stereo matching, structure from motion, and template matching, involve finding a *sparse set* of *salient features* across images followed by matching them. In such cases, the transformation between images, surfaces, or point clouds is typically also parameterized with a small number of parameters, e.g., an affine transform, homography, or a fundamental matrix. These methods are often robust to noise, occlusions, and salient features can be detected and matched efficiently via analytical closed forms. In contrast, dense matching is much harder because the entire image is considered for matching and cannot be reduced to a sparse set of salient features, and the transformation between images is typically parameterized with a large number of parameters, e.g., a dense deformation field. Moreover, dense matching is sensitive to local noise, and cannot be solved efficiently via analytical closed forms—necessitating iterative optimization methods^13–18^. Due to the dense and high-dimensional nature, these methods are often plagued with ill-posedness^12,19,20^, difficulty in optimization, inefficient implementations, and lack of scalability to high-resolution data.

In this work, we focus on dense deformable correspondence matching, which is the non-linear and local (hence deformable) alignment of two or more images into a common coordinate system. Dense deformable correspondence matching is a fundamental problem in computer vision^21^, medical imaging^22–24^, microscopy^25,26^, and remote sensing. Here, we focus on applications in biomedical and biological imaging. In the biomedical and biological sciences, deformable correspondence matching is also referred to as deformable registration. Within dense deformations, *diffeomorphisms* are of special interest as a family of deformations that are invertible transformations such that both the transform and its inverse are differentiable. This allows us to accurately model the correspondence between images while ensuring that the topological structure of the anatomy is preserved, i.e., no tearing or folding of the anatomy is introduced.

We address and tackle two fundamental problems in dense correspondence matching: ill-conditioning and scalability. The ill-conditioning arises due to the high-dimensional and heterogeneous nature of the dense matching optimization objective, that can be mitigated by adaptive optimization methods. Although standard adaptive optimization methods^27,28^ are shown to work in fixed Euclidean spaces, it is not obvious how to extend this formulation to the non-Euclidean space of diffeomorphisms. Fortunately, diffeomorphisms admit many interesting mathematical properties like being embedded in a Riemannian manifold, having a Lie Group structure, and local geodesic formulations that can be exploited for adaptive optimization. We present a *mathematically rigorous* framework for adaptive optimization of diffeomorphic matching Section “Exploiting the group structure of diffeomorphisms”. This is done by exploiting the group structure of diffeomorphisms to define a custom gradient descent algorithm, followed by adaptive optimization on this space. Second, we observe that most existing state-of-the-art methods are prohibitively slow for high-resolution data, which limits their applicability to rigorous hyperparameter studies, large-scale data, or high-resolution alignment at mesoscopic or microscopic resolutions.

Our meticulously implemented operational contributions lead to an algorithm that is around 2 − 7 × faster than state-of-the-art optimization toolkits on CPU, and up to three orders of magnitude faster on GPU. Compared to deep learning methods, our framework demonstrates better generalization to bespoke modalities and species while being competitive in terms of runtime and utilizing upto 10 × less memory. This quantum leap in speedup and scalability also allows us to perform hyperparameter grid search studies with significantly less resources and time compared to traditional and deep learning registration algorithms, and tackle bespoke application areas like sub-micron expansion microscopy, and high-resolution atlas generation in under 25 min. We package our contributions into a software toolkit called *FireANTs*, which is an open-source state-of-the-art toolkit for dense deformable correspondence matching. Our framework can allow a practitioner to perform interactive dense matching that is useful for aligning complex, multimodal, multi-channel, or high-resolution images^29,30^ or to provide guidance to compensate for missing data (e.g., in microscopy imaging), wherein a typical dense image matching algorithm would fail to run interactively.

## Results

FireANTs represents the next generation of frameworks superseding the widely established and successful adoption of the ANTs ecosystem spanning the gamut of biomedical and life sciences research. We evaluate our method on fourteen datasets spanning more than 15,000 image pairs, three organs (brain, lung, abdomen), seven modalities (T1w MRI, T2^*^w MRI, CT, expansion microscopy, LSFM, fMOST, 9.4T MRI), six species (human, ovine, zebrafish, mouse, rat, non-human primates), and show remarkable generalization across all datasets without any domain-specific enhancements. Since our method is a methodological improvement over ANTs, we evaluate FireANTs against established benchmarks where ANTs is one of the top performing methods, among other winning methods for the respective challenges.

FireANTs demonstrates remarkable runtime efficiency compared to ANTs on both CPU and GPU, while also outperforming most deep learning methods at inference runtime and consuming up to a tenth of the GPU memory, setting a new standard for runtime and memory efficiency. This unprecedented efficiency allows a multitude of new capabilities including registering 50 × larger volumes in minutes on a single GPU, faster amortized runtimes over large batches enabling scalable registration of large datasets, efficient hyperparameter grid search studies, and high-resolution atlas building in under 25 min.

### Experiment setup

We briefly describe the significance, existing state-of-the-art and challenges associated with the chosen benchmarks to demonstrate the efficacy of FireANTs. More details about the datasets and evaluation metrics are outlined in Appendix B.

#### In vivo brain mapping challenges^31,32^

Klein et al.^31^ in their landmark paper reported an extensive evaluation of 14 state-of-the-art registration algorithms on four neuroimaging datasets. The four neuroimaging datasets (IBSR18, CUMC12, MGH10, LPBA40) comprise different whole-brain labeling protocols, eight different evaluation measures and three independent analysis methods of over 2000 brain volume pairs. The Learn2Reg^33^ version of the OASIS dataset^32^ is another large scale dataset with 414 subjects for inter-subject brain MRI registration, routinely used as a training dataset for deep learning algorithms^34–36^. Evaluating on these challenges is therefore imperative to establish FireANTs as an effective, versatile and robust algorithm for neuroimaging applications. In total, we compare with state-of-the-art baselines on over *2500 brain volume pairs*, with varying number of labeled anatomical regions and resolutions.

#### PRIMatE Data Exchange (PRIME-DE)^37^

The overarching goal of PRIMatE Data Exchange (PRIME-DE) is to create an open science resource for the neuroimaging community to facilitate the mapping of the non-human primate connectome. The dataset features a familiar modality and anatomy (T1w MRI brain) but different structural organization (non-human primate). This presents a challenge to compare the generalization capabilities of domain-agnostic or foundational registration algorithms with FireANTs.

#### Ultracortex^38^

The Ultracortex dataset hosts a unique collection of ultra-high field (9.4 Tesla) MRI data of the human brain. This challenge provides a complementary problem to PRIME-DE—familiar anatomy and structural organization (human brain) but different modality (9.4T MRI) and resolution (sub-millimeters).

#### Waxholm rat brain and Allen CCFv3 mouse brain datasets^29,39^

The datasets feature high-resolution atlases of the rat and mouse brain with different modalities (T2^*^w MRI and STPT) respectively. The motivation for using these datasets is to provide a benchmark for *cross-species, multimodal* registration. This addresses the growing need to map neuroanatomy across species^40,41^, which is central to revealing the core evolutionary computational motifs and unique adaptations to handle specific ecological and behavioral demands.

#### Lung CT mapping challenges^24,42^

Pulmonary registration has significant clinical applications, including aligning breath-hold scans for visual comparison, modeling lung expansion, and tracking disease progression. Murphy et al. introduced the EMPIRE10 challenge^24^ to facilitate the evaluation of CT lung registration algorithms, including inspiration-expiration, breath-hold over time, 4D, ovine, contrast-noncontrast, and artificially warped scans. EMPIRE10 provides only scan pairs and binary lung masks, withholding fissures and landmarks for *private* evaluation. The scans vary in spatial and physical resolution, necessitating a registration algorithm agnostic to anisotropy in both voxel and physical space. The National Lung Screening Trial (NLST)^42^ subset curated by Learn2Reg challenge is another widely used community-standard dataset. It consists of 210 intra-subject lung pairs, with low-dose helical CT scans with limited field of view and high-dose scans with full field of view, supplemented with more than a thousand keypoints per subject pair. This challenge provides a benchmark for comparison of methods beyond the neuroanatomical domain.

#### RnR ExM mouse isocortex dataset^43^

The RnR-ExM challenge evaluates the ability to perform non linear deformable registration on ultra-high-resolution images. Out of the three species (mouse brain, *C. elegans*, zebrafish), the mouse isocortex dataset is the only dataset with non-trivial non-linear deformations. Registration of high-resolution sub-micron volumes is imperative to creating and understanding the comprehensive cell atlas of the mammalian brain at scale. The voxel size of each image volume is 2048 × 2048 × 81 and the voxel spacing is 0.1625 μm × 0.1625 μm × 0.4 μm. These volume sizes are about two orders of magnitude larger compared to existing biomedical datasets, representing a significant challenge in quick and scalable registration.

#### AZBA and ZBrain datasets^44,45^

The ZBrain atlas is an anatomical and functional reference constructed from high-resolution confocal microscopy images of larval zebrafish (6 days post fertilization) expressing nuclear and cytoplasmic fluorescent markers. The AZBA atlas, in contrast, represents the adult zebrafish brain at cellular resolution. Registration of these templates enables a powerful cross-developmental comparison between the larval and adult zebrafish brains. Conceptually, this task establishes spatial correspondence between larval and adult brain regions, providing a foundation for developmental neuroanatomy. We use this dataset to conduct preliminary experiments to access the generalization capabilities of registration algorithms to cross-developmental data on an unseen species and modality.

#### BICCN mouse dataset

The high-throughput and high-resolution fluorescence micro-optical sectioning tomography (fMOST) platform^46,47^ was used to image 55 mouse brains containing gene-defined neuron populations. The brains are imaged at a resolution of 0.35 × 0.35 × 1.0 μm^3^. The dataset is used to generate a 25 μm-resolution atlas of the mouse brain in under 25 min. This unprecedented scale, enabled by FireANTs, will advance multimodal integration, standardize cross-species comparisons, and drive scalable, reproducible neuroscience research highly pertinent to large-scale collaborative efforts such as BICCN and BICAN.

#### Learn2Reg abdomen MRCT registration^33^

The dataset features intra-patient multimodal abdominal MRI and CT registration (122 scans in total) for diagnostic and follow-up. We use this dataset as a testbed to ablate the effect of Jacobian-free optimization on abdominal MRCT registration.

### Results on generalization to long-tail of modalities

Generalization to unseen modalities, species, resolutions, and anatomical organization is a central requirement for accessible and scalable registration algorithms. Model-free optimization algorithms generalize well on clinical datasets, but the increased heterogeneity on large-scale datasets presents a significant challenge in terms of convergence and runtime. Deep Learning methods typically do not generalize well beyond the data distribution seen during training^34^, although domain-agnostic or foundational methods^36,48^ have shown promising results. Other methods^49^ claim generalization due to architectural design encoding inductive biases for the task. Therefore, we compare FireANTs against ANTs, VoxelMorph as a DL baseline, and SynthMorph, unigradICON, and VFA as other methods that claim generalization to unseen data.

**Fig. 1:**
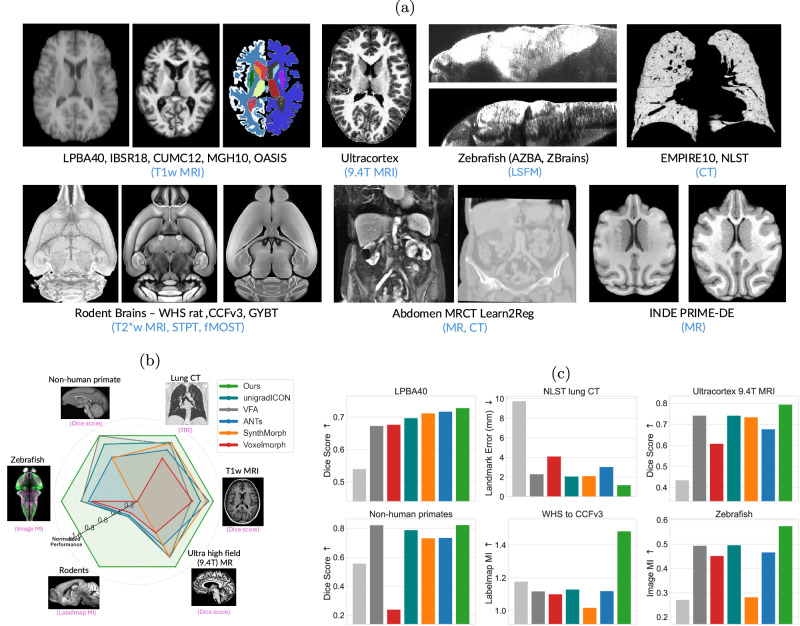
FireANTs can generalize to a large variety of modalities and datasets. Registration quality is validated by measuring either the labelmap overlap, mutual information between aligned labelmap for different labelmaps across datasets, or anatomical landmark distance between the fixed and warped coordinate frames. Across six datasets spanning a spectrum of anatomical systems, species, and modalities, FireANTs achieves the best performance across all evaluation criteria, showcasing its generalization capabilities. **a** Overview of the datasets used in the paper, including two community standard challenges (Klein et al., EMPIRE10) where ANTs was the winner, two analogous contemporary challenges (OASIS, NLST) to enable broader comparison with deep learning methods, and five other scenarios spanning a broad set of challenges. **b** Normalized performance of state-of-the-art registration algorithms across a wide range of datasets and benchmarks. FireANTs achieves asymptotically best normalized performance across various datasets and evaluation criteria. Colorbars are shown with light gray denoting zero displacement (baseline). **c** Raw performance (measured by Dice Score, Mutual Information of Labelmap and Intensity, Landmark Distance) of state-of-the-art registration algorithms across various datasets. In all datasets except NLST, higher scores are better.

Figure 1 shows the performance on six datasets—LPBA40, NLST, Ultracortex, PRIME-DE, Zebrafish (ZBrain and AZBA), and Rodents (Waxholm and CCFv3) encompassing four evaluation criteria (Anatomical Label overlap, Landmark Distance, Mutual information of registered Intensity and Labelmap volumes). The normalized performance is obtained by rescaling the performance of the baseline to 0 and the best performing method to 1. The radar chart shows the generalization of FireANTs across all datasets, and individual plots show unnormalized performance on each dataset. SynthMorph, unigradICON, and VFA perform at par with ANTs on the brain datasets (Ultracortex, PRIME-DE), but severely underperform on the Zebrafish and Rodent datasets. Surprisingly, VFA also underperforms compared to other methods on the LPBA40 dataset, showing a potential weakness in registering highly parceled anatomical regions. FireANTs achieves the best performance on *all* datasets, and performs significantly better on the multimodal cross-species registration task. This establishes FireANTs as a general-purpose registration algorithm that can be used across a wide range of modalities, species, and resolutions.

### Results on state-of-the-art biomedical benchmarks

FireANTs proposes an algorithmic improvement over the state-of-the-art ANTs toolkit. As such, we compare FireANTs with ANTs on community standard benchmarks where ANTs is established as one of the top performing methods. This include Klein et al.^31^ neuroimaging challenge, EMPIRE10^24^ pulmonary challenge. We also add comparisons on two contemporary benchmarks for neuroanatomy (OASIS) and pulmonary (NLST) datasets from the Learn2Reg^33^ challenge, which is of high relevance to the neuroimaging and pulmonary communities.

#### In vivo brain MRI mapping

**Fig. 2:**
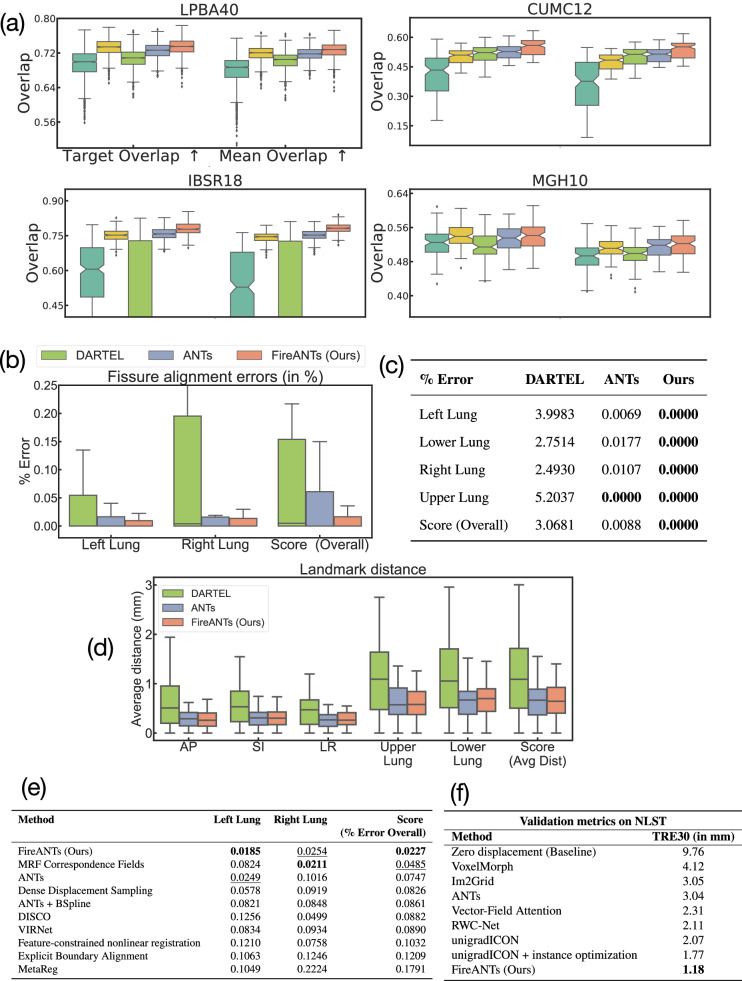
FireANTs demonstrates state-of-the-art performance on community-standard neuroimaging and pulmonary challenges. **a** Following the evaluation setup of ref. ^31^, we validate registration performance using the average volume overlap of all anatomical label maps between the fixed and warped label maps on four datasets: IBSR18 (*n* = 306), CUMC12 (*n* = 132), MGH10 (*n* = 90), and LPBA40 (*n* = 1560). Evaluation is shown for five metrics with *↑* denoting a higher score is better, and *↓* signifying a lower score is better. FireANTs shows significant gains in performance that are consistent across all four datasets, with the median overlap scores outperforming the third quartile of all other methods for IBSR18 and CUMC12 datasets. **b** On the EMPIRE10 dataset (*n* = 30), FireANTs achieves substantially lower inter-quartile range of fissure errors, defined as the percentage of marked pixels that are registered to points on the opposite side of the fissure boundary. Overall 30 scan pairs, our method performs 5 × better than ANTs. **c** (EMPIRE10) Singularity errors are defined as fraction of voxels that define a non-invertible deformation, quantifying implausible deformations. FireANTs achieves *zero percent* singularity errors. **d** (EMPIRE10) Landmark distance is the Euclidean distance between well-dispersed landmark points between the fixed and warped images. FireANTs have a lower median and narrower interquartile range than baselines on five out of six subregions. **e** (EMPIRE10) Fissure alignment error on top 10 algorithms in the challenge, sorted by fissure alignment error, averaged on all scan pairs. FireANTs outperforms a wide array of baselines, including direct optimization (ANTs, ANTs + BSpline), neural networks (VIRNet), and explicit correlation volumes (MRF, Disco). Best result is shown in bold, and the second-best result is shown in italics. **f** (NLST) Landmark distance in mm for provided landmarks. Our method outperforms a variety of state-of-the-art optimization and deep learning algorithms (*n* = 10). All box plots are annotated as follows: the center line is the median; box bounds are the interquartile range (25th–75th percentiles); whiskers extend to the minimum and maximum values within 1.5 × IQR of the lower/upper quartiles; points beyond the whiskers are outliers.

We compare FireANTs with two state-of-the-art optimization algorithms on Klein et al.^31^: ANTs—which won the original challenge, and Symmetric Log Demons^18^, and two widely used deep learning algorithms: VoxelMorph^34^ and SynthMorph^48^ using their provided pretrained models. Since no methods utilize label maps, we run registration on all 414 image pairs prescribed in the dataset. Results for the brain datasets are shown in Fig. 2a, Supplementary Fig. 7 and Supplementary Table 1. Our algorithm outperforms all baselines on four out of five datasets, with an improvement in *all* metrics evaluating the volume overlap of the fixed and warped label maps. The improvements are consistent across varying parcellations and relative sizes of anatomical label maps. In the IBSR18 and CUMC12 datasets, the median target overlap of our method is better than the third quantile of ANTs. Supplementary Fig. 7 also highlights the improvement in label overlap per labeled brain region across all datasets. For deep learning methods, a noticeable performance drop is observed when the anisotropic volumes are fed into the network, which is undesirable as the trained model is essentially ‘locked’ to a single physical resolution—which limits the generalizability of the model to various modalities with different physical resolutions. For Demons, ANTs, and FireANTs (Ours), we do not perform any additional normalization or resampling. On the OASIS dataset, all methods perform at par with each other with no significant differences. SynthMorph is more robust to the domain gap than VoxelMorph due to its training strategy with synthetic images, but still underperforms optimization baselines when their recommended hyperparameters are chosen.

#### Lung CT mapping challenges

**Fig. 3:**
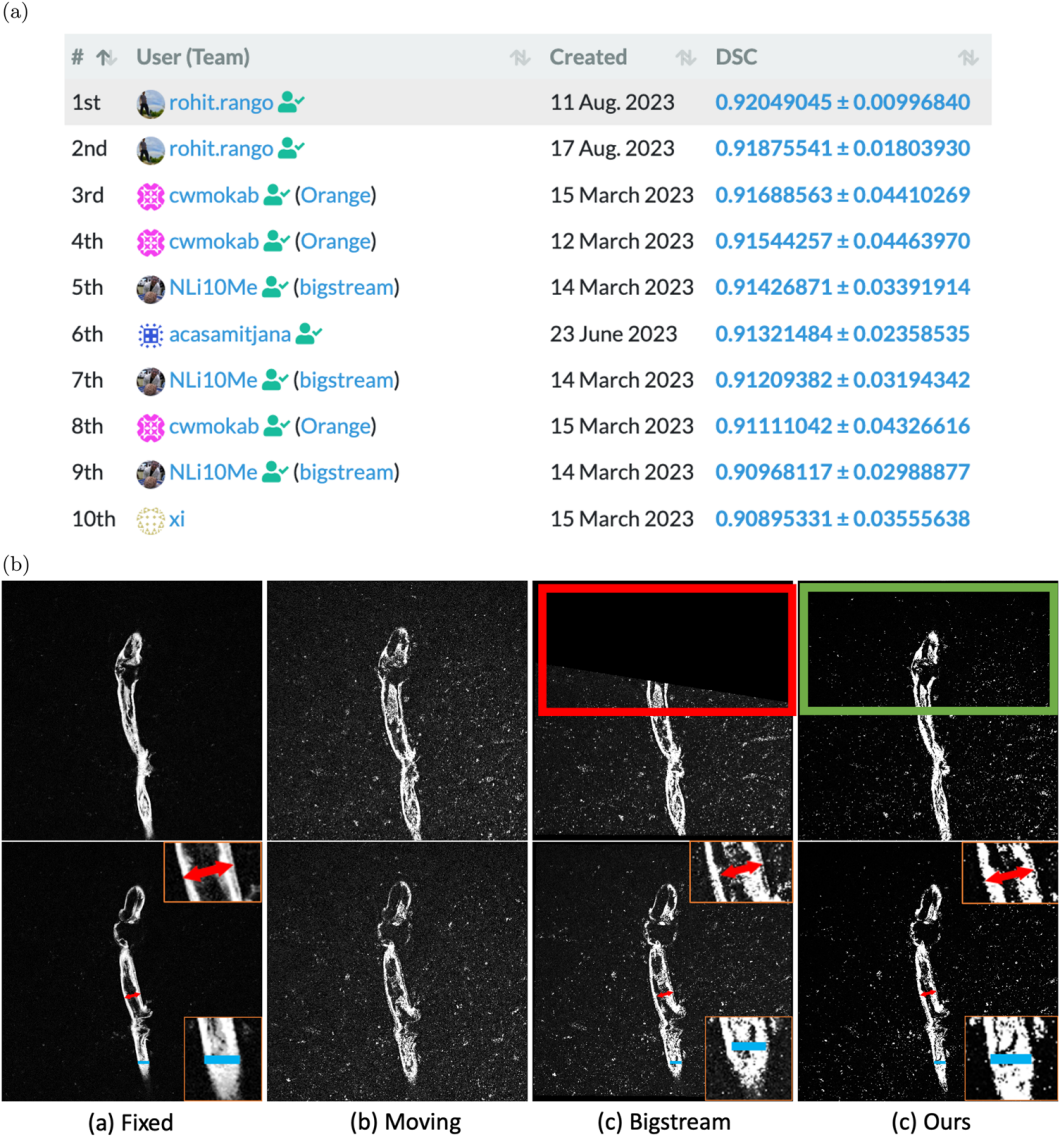
FireANTs secures first rank in the RnR-ExM mouse dataset. **a** As of March 4, 2026, our method ranks first in the mouse brain registration task, which is the only task in the challenge requiring deformable registration. Our top two successful submissions secure the first and second position, with a 0.361 improvement in Dice score compared to the 3rd ranked submission, which is 0.261 better than the 5th ranked submission (bigstream). Note that among the top 10 submissions, our method has the lowest standard deviation (4.42 × lower than the second-best submission), showing the robustness of our model across different microscopy volumes. **b** shows a qualitative comparison of FireANTs with Bigstream, the other top leading method in the challenge. The moving image volumes have substantially more noise than the fixed image volumes, making intensity-based registration difficult. The non-rigid deformation dynamics of the hydrogel are clearly visible, as the moving volume has a thicker boundary than the fixed volume. Bigstream does not capture these dynamics very well—this is illustrated by comparing the thickness of the cortex at various points (zoomed orange crops in bottom row), where Bigstream does not deform the cortex enough to match the fixed image. FireANTs deforms and accurately depicts these morphological changes, which can be crucial for downstream morphometric studies. Moreover, the affine registration in Bigstream knocks the boundary slices out of the volume (red highlight in top row), leading to drop in registration performance. On contrary, our method’s affine and deformable stages are more stable, leading to better registration and avoiding spurious out-of-bound artifacts at the boundary slices.

The EMPIRE10 lung dataset^24^ consists of volumes that are about 10 × larger than the brain dataset, thereby presenting a scaling challenge for deformable registration algorithms. Evaluation is done with privately withheld labels; we use the provided results from the leaderboard to compare with other methods. We evaluate three criteria: (1) fissure alignment errors (%)—the fraction of misaligned fissure voxels (Fig. 2b, e), (2) landmark distance in mm (Fig. 2d), and (3) singularity errors—the fraction of non-diffeomorphic voxels (Fig. 2c). Figure 2 highlights the impact of representation choice in modeling diffeomorphisms. DARTEL, using an exponential map, performs significantly worse than ANTs across all metrics by three orders of magnitude. In contrast, our method reduces fissure alignment error by 5 × compared to ANTs and outperforms it in 5 out of 6 landmark subregions. While all methods theoretically ensure diffeomorphism, SVF-based approaches introduce singularity errors due to non-adaptive scaling-and-squaring. We discuss the numerical limitations of SVF-based approaches in Appendix E. ANTs also introduces some singularities, whereas our method computes numerically perfect diffeomorphic transforms. Finally, Fig. 2e compares fissure alignment errors among EMPIRE10 submissions, showing FireANTs achieves the lowest landmark errors and the fastest runtime among the top 10 methods, setting new benchmarks in computational efficiency and accuracy. NLST: for the NLST dataset^42^, we compare with representative state-of-the-art optimization and deep-learning baselines. We use the evaluation criteria provided by the challenge and measure results on the Robust Target Registration Error (TRE30) in millimeters between the registered keypoints. Results in Fig. 2f show that FireANTs outperforms all baselines on the NLST dataset, with improvements of upto 51.6% in robust target registration error (TRE30) of provided keypoints compared to state-of-the-art deep learning benchmarks, including Im2Grid, Vector-Field Attention, RWC-Net, and a 50.8% improvement in TRE30 over foundation models like unigradICON. This demonstrates the broad applicability of FireANTs beyond neuroimaging applications.

### Evaluation of high-resolution mouse isocortex registration

Expansion microscopy (ExM) is an emerging super-resolution fluorescence imaging technique that enables 3D nanoscale visualization of cellular and molecular structures^50^. While ExM provides rich structural data, its large-scale images remain challenging for existing registration algorithms due to repetitive textures, highly non-linear hydrogel deformations, imaging noise, and size constraints. The Robust Non-rigid Registration Challenge for Expansion Microscopy (RnR-ExM)^43^ offers a benchmark dataset, where we focus on registering mouse isocortex images, characterized by hydrogel-induced deformations and staining intensity loss. Each volume (2048 × 2048 × 81 voxels) has a voxel spacing of 0.1625 μm ×  0.1625 μm × 0.4 μm and is 40.5 times larger than brain imaging datasets. Current state-of-the-art methods either register small independent chunks, losing inter-chunk information, or process highly downsampled images^35^, significantly reducing resolution (by 64 × in-plane).

In contrast, FireANTs is able to register the volume at native resolution. We perform an affine registration followed by a diffeomorphic registration step. The entire method takes about 2–3 min on a single A6000 GPU. As shown in Fig. 3, our method secures the first place on the leaderboard, with a considerable improvement in the Dice score and a 4.42 × reduction in the standard deviation of the Dice scores compared to the next best method. Figure 3 also shows a qualitative comparison of our method compared to Bigstream, the winner of the RnR-ExM challenge. Bigstream performs only an affine registration, leading to inaccurate registration in one of three test volumes, leading to a lower average Dice score and higher variance. Moreover, the affine registration leads to boundary in-plane slices being knocked out of the volume, leading to poor registration (Fig. 3). FireANTs preserves the boundary in-plane slices during its affine step, and subsequently performs an accurate diffeomorphic registration at submicron resolution, leading to accurate registration with substantially lower variance. This experiment demonstrates the versatility and applicability of FireANTs for high-resolution microscopy registration.

### Runtime and memory efficiency analysis

One of the critical bottlenecks for scalable registration with ANTs is the prohibitively large runtimes for single-threaded CPU registration^34^. Deep learning methods aim to reduce the runtime by performing feedforward inference, but these methods in turn have steep memory requirements due to activation overheads^51,52^, making them infeasible for high-resolution registration. FireANTs circumvents both these issues using a lightweight implementation on GPU.

#### Runtime compared to ANTs

**Fig. 4:**
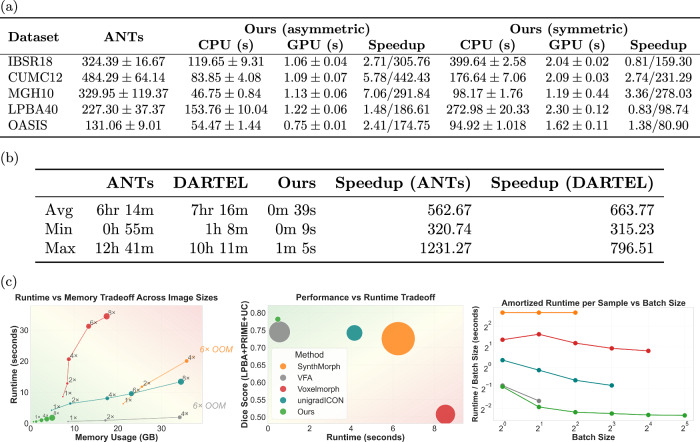
FireANTs facilitates quick and scalable registrations. We compare the runtime of our implementation with the ANTs library. **a** shows summary statistics of speedup (runtime of ANTs divided by runtime of our method) and runtimes (in seconds) for the five brain MRI datasets. For all datasets, our implementation runs two orders of magnitudes faster, making it suitable for hyperparameter search algorithms, and larger datasets. **b** shows the runtime of ANTs, DARTEL and our implementation on the EMPIRE10 challenge data. The first three columns show the actual runtime of the methods, followed by the speedup obtained by our method when compared to ANTs and DARTEL. Note that our method runs a *minimum* of 320 times faster than ANTs, saving a substantial amount of time, at no loss in registration quality. **c** shows the runtime and memory requirements of our method compared to deep learning methods. Left shows the runtime and memory requirements of our method compared to deep learning methods for increasing problem sizes. FireANTs is upto 10 × more memory efficient than SOTA deep learning methods, while performing faster than almost all of them at inference. Middle shows the plot of average performance over three brain datasets compared with average runtime, with the size of the bubble indicating memory usage. FireANTs performs *better* while being faster and more memory efficient than all deep learning methods, indicating that a tradeoff is not necessary for good performance. Right shows that further gains in amortized runtime are possible by increasing the batch size at inference. FireANTs achieves less than 0.25 s per image pair and runs more than double the number of image pairs compared to all other deep methods, showing unprecedented efficiency for high-throughput registration.

We evaluate the efficiency of FireANTs compared to ANTs by running both algorithms on the CPU with 32 threads, with identical multi-scale optimization settings. Furthermore, we run FireANTs on the GPU with a batch size of 1 to avoid amortizing runtimes over larger batches. The runtimes on all five brain datasets (IBSR18, CUMC12, MGH10, LPBA40, and OASIS) are shown in Fig. 4a. FireANTs is upto 7 × faster than ANTs on CPU, and 442 × faster on GPU. The runtime improvement on the CPU can be attributed to faster convergence and better implementation of the optimization since both methods are run with identical multi-scale optimization settings and capped at the same CPU resources. Figure 4b shows the runtime of FireANTs compared to ANTs on the EMPIRE10 dataset. Since the runtime for ANTs and DARTEL are provided in the submission writeup without details on hardware or number of threads, it is not possible to reproduce the same results, and use their provided numbers as expected runtime for the dataset. However, FireANTs runs an average of 560 × faster than ANTs on the GPU, which is at par with the runtime improvements on neuroimaging datasets.

#### Runtime and memory requirements compared to deep learning methods

FireANTs is highly efficient compared to deep learning methods, both in terms of runtime and memory usage. We highlight the efficiency using three experiments:

- Accuracy-Runtime tradeoff: we compare the performance, runtime, and memory usage of FireANTs with SOTA deep learning methods averaged over three neuroimaging datasets (LPBA40, Ultracortex, PRIME-DE).
- Runtime and Memory requirements with increasing problem sizes: we take an OASIS MRI image pair and progressively upsample it by factors of 2×, 4×, 6×, 8× larger and measure the runtime and memory usage of each method.
- Amortized runtime with increasing batch sizes: amortized runtime over larger batches can improve GPU utilization and hide kernel launch and activation cache loading overheads. We perform batched inference using an OASIS MRI volume for increasing batch sizes, and plot the amortized runtime (i.e. runtime divided by batch size) as a function of the batch size, and keep increasing the batch size until we run out of memory.

For all methods, we measure the runtime only for the registration call function, not including image loading, preprocessing and postprocessing steps, model loading, etc. We note that if these steps were to be included in the runtime comparison, the efficiency gains of FireANTs would be even more significant.

These results in Fig. 4c show that FireANTs is upto 10 × more memory efficient than SOTA deep learning methods, while performing faster than most of them at inference. This is a result that challenges the common belief that deep learning methods are faster than iterative optimization methods at inference, similar to results shown in Hering et al.^33^. Deep learning feedforward inference can be slow due to convolutions of large activations, skip connections requiring repeated memory accesses. In contrast, the iterative optimization updates in FireANTs are lightweight and can be run for more iterations. Since FireANTs does not generate any feature activations beyond that in the loss function, it is very memory efficient. Over three brain datasets, FireANTs achieves the best accuracy-runtime performance, showing that a tradeoff is not necessary for good performance. Amortized inference can further improve efficiency—on the OASIS dataset, FireANTs can register a batch of 32 image pairs in less than 0.25 seconds per pair. This unprecedented efficiency paves the way for rapid prototyping, hyperparameter tuning, and scaling to high resolution datasets. This sets a new standard for high-throughput image registration on GPUs.

#### FireANTs enables rapid prototyping and hyperparameter tuning

In optimization toolkits such as ANTs, correct choice of hyperparameters are key to high quality registration. Some of these hyperparameters are the window size for the similarity metric Cross-Correlation or bin size for Mutual Information. In our experience, the Gaussian smoothing kernel ${\sigma }_{{{{\rm{grad}}}}},{\sigma }_{{{{\rm{warp}}}}}$ for the gradient and the warp field are two of the most important parameters for accurate diffeomorphic registration. The optimal values of these hyperparameters vary by image modality, intensity profile, noise and resolution. Typically, these values are provided by some combination of expertise of domain experts and trial-and-error. However, non experts may not be able to adopt these parameters in different domains or bespoke acquisition settings. Recently, techniques such as hyperparameter tuning have become popular, especially in deep learning^53^.

**Fig. 5:**
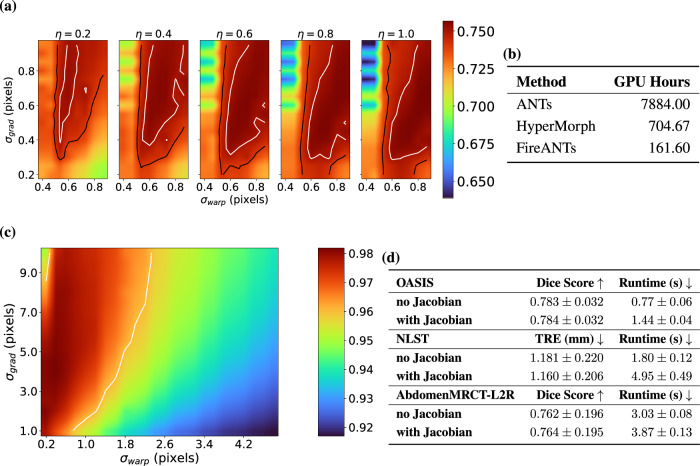
FireANTs facilitates feasibility of extensive hyperparameter search in registration. The speed of FireANTs makes hyperparameter studies like these feasible, which ANTs would take years to complete. **a** We perform a hyperparameter grid search on three hyperparameters of interest—smoothing kernel for the warp field (*σ*_warp_) in pixels, smoothing kernel for the gradient of warp field (${\sigma }_{{{{\rm{grad}}}}}$) in pixels and learning rate *η*. The metric to optimize in this case is the target overlap. For the LPBA40 dataset, we perform a hyperparameter sweep over 640 configurations in 40 h with 8 A6000 GPUs. A corresponding hyperparameter sweep with 8 concurrent jobs with each job consuming 8 CPUs would take  ~ 3.6 years to complete. The white contour representing the level set for target overlap = 0.75, and the black contour representing the level set for target overlap of 0.74 show the robustness of our method to hyperparameters—performance is not brittle or sensitive to choice of hyperparameters. **b** Efficiency of FireANTs on the LPBA40 dataset compared to ANTs and HyperMorph, measured in GPU hours (CPU hours for ANTs). Even with amortized hyperparameter optimization in HyperMorph, FireANTs is about 4.3 × more efficient than HyperMorph. **c** Hyperparameter grid search on the EMPIRE10 dataset over *σ*_warp_ and ${\sigma }_{{{{\rm{grad}}}}}$ parameters (456 configurations), with a fixed learning rate of *η* = 0.25. The metric to optimize is the Dice score of the provided binary lung mask. This sweep takes about 12.37 h on 8 GPUs, whereas a corresponding sweep would take 296 days for ANTs and 345 days for DARTEL (more in Fig. 4). The white contour corresponds to the level set for Dice score = 0.96, showing both a huge spectrum of parameters that achieve high Dice scores, and low sensitivity of the method to choice of hyperparameters. **d** Ablation study on the effect of Jacobian-free optimization on the OASIS, NLST, and AbdomenMRCT-L2R datasets, spanning three organ types and modalities. Jacobian-free optimization brings no penalty in Dice score, but significantly reduces the runtime, making convergence upto 2.75 × faster.

In the case of registration, hyperparameter search can be performed by considering some form of label/landmark overlap measure between images in a validation set. We demonstrate the stability and runtime efficiency of our method using two experiments: (1) Owing to the fast runtimes of our implementation, we show that hyperparameter tuning is now feasible for different datasets. The optimal set of hyperparameters is dependent on the dataset and image statistics, as shown in the LPBA40 and EMPIRE10 datasets; (2) within a particular dataset, the sensitivity of our method around the optimal hyperparameters is very low, demonstrating the robustness and reliability of our method. We choose the LPBA40 dataset among the 4 brain datasets due to its larger size (40 × 39 = 1560 pairs). We choose three parameters to search over : the learning rate (*η*), and the gaussian smoothing parameters ${\sigma }_{{{{\rm{warp}}}}},{\sigma }_{{{{\rm{grad}}}}}$. We use the Ray library (https://docs.ray.io/) to perform a hyperparameter tuning using grid search. For the LPBA40 dataset, a grid search over three parameters (shown in Fig. 5a) takes about 40.4 h with 8 parallel jobs. On the contrary, ANTs would require around 3.6 years to complete the same grid search, with 8 threads allocated to each job and 8 parallel jobs. This makes hyperparameter search for an unknown modality computationally tractable. A deep learning solution like HyperMorph^53^ can perform amortized training over a predefined hyperparameter space, but still requires significant GPU hours for training and inference of 1560 pairs for each configuration to generate a plot like Fig. 5a. Figure 5b shows the runtime and memory usage of FireANTs, ANTs, and HyperMorph on the LPBA40 dataset, showing that even a brute force grid search with FireANTs is about 4 × faster than state-of-the-art amortized hyperparameter learning.

#### FireANTs is robust to a wide range of hyperparameters

Figure 5a shows a dense red region suggesting the final target overlap is not sensitive to the choice of hyperparameters. Specifically, the maximum target overlap is 0.7565 and 58.4% of these configurations have an average target overlap of ≥0.74. This is demonstrated in Fig. 5a by the white contour line denoting the level set for target overlap = 0.75, and the black contour line denoting the level set for target overlap of 0.74. The target overlap is quite insensitive to the learning rate (≥0.4) showing that our algorithm achieves fast convergence with a smaller learning rate. On the EMPIRE10 dataset, we fix the learning rate and perform a similar hyperparameter search over two parameters, the Gaussian smoothing parameters ${\sigma }_{{{{\rm{warp}}}}},{\sigma }_{{{{\rm{grad}}}}}$, shown in Fig. 5c. We use the average Dice score between the fixed and moving lung mask to choose the optimal hyperparameters. FireANTs can perform a full grid search over 456 configurations on the EMPIRE10 dataset in 12.37 h with 8 A6000 GPUs, while it takes SyN 10.031 days to run over a single configuration. Normalizing for 8 concurrent jobs and 456 configurations, it would take ANTs about 296 days, and DARTEL about 345 days. This shows that our method and accompanying implementation can now make hyperparameter search for high-resolution 3D image registration studies feasible.

#### Runtime efficiency due to Jacobian-free optimization

A key methodological approach proposed in this work is the Jacobian-free approximation of the gradient field for faster diffeomorphic optimization (Section “Exploiting the group structure of diffeomorphisms”). A concern that may arise is the effect of this approximation on the accuracy of the registration, specifically due to the assumption that the Jacobian is positive-definite. We ablate on the effect of this approximation on the accuracy of the registration on three datasets—OASIS, NLST, and AbdomenMRCT-L2R, spanning three organ types and modalities. The heterogeneity and variable biomechanics and deformation dynamics across multiple organ systems is a challenging testbed for measuring the effect of Jacobian-free optimization on registration accuracy. Figure 5d shows that the Jacobian-free approximation does not significantly affect the accuracy of the registration, with a maximum Dice score difference of 0.002 and a maximum TRE difference of 0.021 mm. However, avoiding computation of the Jacobian-augmented descent direction significantly reduces the runtime of the registration, making convergence upto 2.75 × faster. Turning on the Jacobian-augmented descent direction is easy in our implementation by only changing a flag during runtime, but we recommend turning it off for most applications. In all our experiments except this ablation study, we do not compute the Jacobian-augmented descent direction.

### FireANTs facilitates scalable atlas generation

**Fig. 6:**
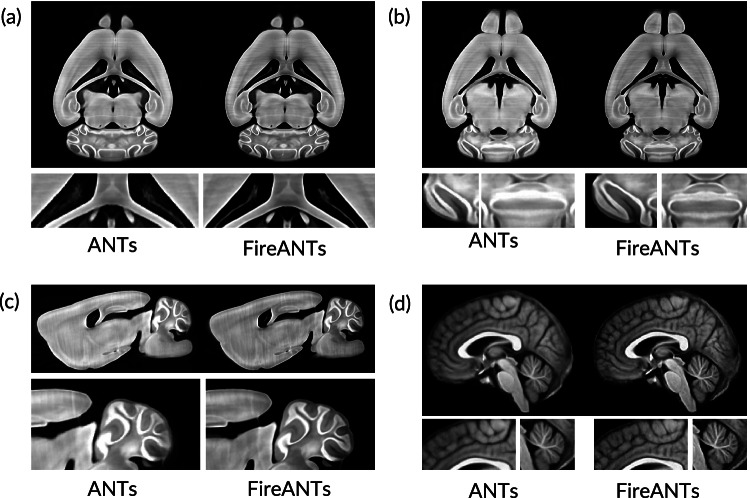
Comparison of brain templates (atlases) constructed using ANTs (left) and FireANTs (right). **a**–**c** Coronal and sagittal sections of the 25 μm fMOST mouse brain template illustrate the improved structural fidelity of FireANTs. In the ANTs template, the internal regions of the lateral ventricles appear blurred (**a**), and the cerebellar architecture exhibits intensity bleeding (**b**, **c**), whereas FireANTs yields crisper delineation of these anatomical structures. **d** The in vivo human brain atlas further demonstrates the advantages of FireANTs, with sharper cortical folding and improved contrast and realistic intensity features in the cerebellum compared to ANTs. FireANTs generates multiple high-fidelity templates while being 200–400 times faster than ANTs.

Atlas generation is an important component of integrating large-scale imaging data—including gene expression, connectivity patterns, and functional properties—onto a common spatial coordinate system facilitating multimodal data alignment and comparison. This requires atlas (or template) generation capabilities that scale with the unprecedented scale of acquired data. In this section, we showcase the efficiency of atlas generation by reproducing the fMOST atlas proposed in the ANTsX ecosystem^54^ for the mouse brain. We follow the steps outlined in the ANTsX Ecosystem^54^ for generating an fMOST atlas of the mouse brain, including preprocessing steps like downsampling to 25 μm resolution, destriping, flipping along the sagittal plane for left-right symmetry, bias field correction, and affine preregistration to a common template. Since no parcellations are available for the dataset, we qualitatively compare the atlases generated by ANTs and FireANTs, and compare their runtimes. We also generate an in vivo atlas for the OASIS dataset, to show scalability on smaller datasets and for quantitative evaluation. Figure 6 shows that the atlas generated by both methods is similar in terms of quality. On a 64-thread core machine, ANTs takes 141.5 h to generate the atlas with 6 epochs of template refinement. With the identical number of iterations and configuration, FireANTs runs in 22 min with a distributed setup on an 8-GPU workstation, showing a significant improvement in runtime efficiency. On a much lower-resolution OASIS dataset, ANTs takes 2 h and 16 min to generate an atlas with 16 subjects, while FireANTs runs in 32 s. To quantify atlas fidelity, we evaluate Dice Score overlap of image pairs after registering them to the atlas. While pairwise Dice score overlap of subjects is 0.704 ± 0.163 with the ANTs template, the FireANTs template improves the Dice score to 0.722 ± 0.161. This demonstrates that FireANTs can be used to generate high-fidelity atlases two orders of magnitude faster than ANTs at no loss in image quality, making it a powerful tool for large-scale atlas generation.

### Independent evaluation

Since the release of our code and documentation, FireANTs has been independently adopted by researchers in the field. Few anecdotal examples include the registration of high-resolution histology slides, and non-human primate data^55,56^. A compelling independent application and evaluation is performed by NextBrain^57^, a tool that utilizes FireANTs to perform Bayesian segmentation of in vivo and ex vivo brain MRI scans. NextBrain uses FireANTs to register input brain MRI scans to an augmented template with a resolution of 200 μm and 333 regions of interest, providing a comprehensive structural analysis of the subject. Quantitatively, incorporating FireANTs in the pipeline leads to no loss in performance as measured by Dice overlap. The utilization of GPU-based toolkits including FireANTs reduces the runtime from 2–3 days / 1 week for 1 mm in vivo / 300 μm ex vivo scans on a multi-core workstation, to less than 5 min on a GPU. Owing to the robustness and efficiency of FireANTs, it is set as the default registration method in NextBrain. Another independent evaluation is performed on the registration of high-resolution X-ray images^58^, where FireANTs establishes itself as a strong baseline, outperforming several baselines specialized for X-ray image registration. This demonstrates the accessibility, scalability and efficiency of FireANTs in real-world applications.

## Discussion

We present FireANTs, a powerful and general-purpose multi-scale registration algorithm. Our method performs registration by generalizing the concept of first-order adaptive optimization schemes for optimizing parameters in a fixed Euclidean space, to multi-scale *diffeomorphisms*. This generalization is highly non-trivial because diffeomorphisms are typically implemented as an image grid proportional to the size of the fixed image, and are optimized in a multi-scale manner to capture large deformations^13,17,59^ leading to changing grid size throughout optimization. Our method also avoids computationally expensive parallel transport and Riemannian metric tensor computation steps for diffeomorphisms by solving an Eulerian descent that exploits the group structure to define descent directions from the identity transform. FireANTs achieves consistent improvements in performance over state-of-the-art registration algorithms like ANTs, DARTEL, SynthMorph, VFA, unigradICON, and Bigstream. This improvement is shown across fourteen datasets encompassing a broad spectrum of anatomical systems, contrast, image volume sizes, species, and modalities. A key advantage of our method is that *we do not tradeoff* any of accuracy, speed, or robustness for the others, thus being a powerful registration algorithm.

FireANTs generalizes to a long tail of out-of-distribution image modalities and datasets. Although a substantial amount of research is focused on in vivo neuroimaging datasets and methods, a plethora of biomedical and life science application areas beyond in vivo neuroimaging require a common coordinate frame for downstream quantitative analysis. Building customized solutions from scratch for each application area requires significant domain expertise, resources, and time, and may only provide marginal additional value or insights on top of a general purpose registration algorithm. On the contrary, FireANTs allows researchers to focus on the application-specific aspects of the problem by providing a powerful base framework for accurate and fast registration, that can be easily extended to the specific application domain using customized loss functions, regularization terms, preconditioning strategies, and learnable feature representations^60,61^. Beyond community standard registration in vivo neuroimaging^31,32^ and pulmonary imaging^24^ benchmarks that are widely regarded as a comprehensive evaluation of registration algorithms, FireANTs shows remarkable generalization on high-resolution microscopy imaging^43^, X-ray imaging^58^, non-human primate neuroimaging^37^, sub-millimeter ultra-high field MRI scans^38^, multimodal rodent brains^29,39^, and zebrafish^44,45^. FireANTs achieves robust and accurate registration across a wide spectrum of anatomical systems, image scales, species, and modalities, providing a versatile foundation and modular framework that researchers can readily tailor to their specific application domains.

This study draws attention to an often overlooked source of performance variation - the choice of diffeomorphism representation (direct optimization versus exponential map), which motivated our adoption of direct optimization in FireANTs. This improvement can be attributed to the representation—one can interpret direct optimization as integrating a set of *time-dependent* velocity fields since the gradients change throughout optimization, allowing more flexibility in the space of diffeomorphisms it can represent, whereas SVF performs the integral of a *time-independent* velocity field by design. Using the SVF representation has three main drawbacks, which we mathematically formalize in Appendix E. First, the scaling-and-squaring step is computationally expensive^17,62^—requiring 7–8 repeated warp compositions to obtain the diffeomorphism, and another 7–8 steps of compositions to compute the gradient, compared to only 1 warp composition for direct optimization. Second, the sensitivity of the diffeomorphism depends on its magnitude, i.e., gradients with similar magnitudes will lead to larger updates in the diffeomorphism for larger velocity fields, which can lead to numerical instability for large deformations. Third, the number of integration steps to ensure numerical diffeomorphisms depends on the Lipschitz constant of the velocity field, which requires adaptive Euler integration step sizes to ensure numerical diffeomorphisms. Since most practitioners choose a fixed Euler integration step size regardless of the problem, choosing too few steps can lead to numerical inaccuracies while choosing too many steps can lead to high computational cost or numerical underflow in the base case. Empirically we observe the exponential map representation (DARTEL) takes substantially longer to run than ANTs (Fig. 4a). In Fig. 2, the results for shooting methods are substantially worse than for methods that optimize the transformation directly. We also observe this for the LPBA40 dataset in Supplementary Fig. 12, where the shooting method consistently underperformed over a wide range of hyperparameter choices. Modern deep learning methods have also moved away from SVF representations towards compositive (albeit non-diffeomorphic) updates^49,63^.

FireANTs is upto three orders of magnitude faster than ANTs on GPUs, and upto 10 × memory efficient than deep learning methods while having highly competitive runtimes. On the CPU, FireANTs is upto 7 × faster than ANTs under identical computational budget, owing to an efficient implementation and faster convergence. This efficiency allows for high-resolution registration of large microscopy datasets on a single GPU, grid search hyperparameter studies that are faster than amortized hyperparameter learning methods, amortized batched inference for large datasets, and high-resolution atlas generation in less than 25 min. This unparalleled throughout will enable fast and accurate registration of high-resolution mesoscale and microscale imaging data that will play a paramount role in advancing our understanding of connectomics, neuroscience, cellular and molecular biology, genetics, pathology, among many other disciplines in the biomedical and biological sciences. With breakthrough advances in high-resolution, high-throughput imaging techniques, it is imperative for registration algorithms to also scale with inordinate amounts of data. In summary, FireANTs is a powerful and general-purpose multi-scale registration algorithm and sets a new state-of-the-art benchmark. We propose to leverage the accurate, robust, and fast library to speed up registration workflows for ever-growing needs of performant and fast image registration in a spectrum of disciplines within the biomedical and biological sciences, wherein algorithms are bottlenecked by scalability.

## Methods

### Preliminaries

Given *d*-dimensional images $I:\Omega \to {{\mathbb{R}}}^{d}$ and $I{\prime} :\Omega \to {{\mathbb{R}}}^{d}$ where the domain Ω is a compact subset of ${{\mathbb{R}}}^{2}$ or ${{\mathbb{R}}}^{3}$, image registration is formulated as an optimization problem to find a transformation *φ* that warps $I{\prime}$ to *I*. The transformation can belong to an algebraic group, say *G*, whose elements *g* ∈ *G* act on the image by transforming the domain as (*I*∘*g*)(*x*) = *I*(*g*(*x*)) for all *x* ∈ *Ω*. The registration problem solves for

1 $${\varphi }^{*}={{{{\rm{argmin}}}}}_{\varphi \in G}L(\varphi ) \, \doteq \, C(I,I^{\prime} \circ \varphi )+R(\varphi )$$

where *C* is a cost function, e.g., that matches the pixel intensities of the warped image with those of the fixed image, or local normalized cross-correlation or mutual information of image patches. There are many types of regularizers *R* used in practice, e.g., total variation, elastic regularization^64^, enforcing the transformation to be invertible^65^, or volume-preserving^66^ using constraints on the determinant of the Jacobian of *φ*, etc. If, in addition to the pixel intensities, one also has access to label maps or different anatomical regions marked with correspondences across the two images, the cost *C* can be modified to ensure that *φ* transforms these label maps or landmarks appropriately.

#### Properties of the considered transformation group

A diffeomorphism is a smooth and invertible map with a corresponding differentiable inverse map^67–69^. We denote the set of all diffeomorphisms on Ω as $\,{{{\rm{Diff}}}}\,(\Omega ;{{\mathbb{R}}}^{d})$. It is useful to note that unlike rigid or affine transforms that have a fixed number of parameters, diffeomorphisms require dense and variable parameterization, typically proportional to the size of the image. When groups of transformations on continuous domains are endowed with a differentiable structure, they are called Lie groups. Diffeomorphisms are also examples of Riemannian manifolds, and are amenable to Riemannian optimization (see Section “Adaptive optimization for diffeomorphisms”).

In this work, we only consider a subgroup of diffeomorphisms. Consider the set of continuously differentiable functions $u\in {C}_{0}^{1}(\Omega,{{\mathbb{R}}}^{d})$ such that *u*, *J*(*u*) = 0 on ∂Ω, where *J*(*u*) is the Jacobian of *u*, such that ${[\; J(u)(x)]}_{ij}=\frac{\partial u{(x)}_{i}}{\partial {x}_{j}}$. These functions can be extended to have *u* ≡ 0 outside Ω. Then, for a small enough *ϵ* > 0, *x* + *ϵu*(*x*) is a diffeomorphism (Proof in Proposition 8.6 in ref. ^70^). Although these diffeomorphisms are close to identity, diffeomorphisms with larger deviations from the identity can be constructed by composing these ‘small diffeomorphisms’. Therefore, we study the subgroup of diffeomorphisms of the form

2 $${\phi }_{n}=(id+{\epsilon }_{1}{u}_{1})\circ \ldots \circ (id+{\epsilon }_{n}{u}_{n})$$

where *u*_*i*_s are defined as before. We denote this subgroup as $G(\Omega,{{\mathbb{R}}}^{d})$. This subgroup retains the group structure with identity element *id*, the composition operation ∘ induced from $\,{{{\rm{Diff}}}}\,(\Omega,{{\mathbb{R}}}^{d})$, and the inverse group element: ${\phi }_{n}^{(-1)}={(id+{\epsilon }_{n}{u}_{n})}^{(-1)}\circ \ldots \circ {(id+{\epsilon }_{1}{u}_{1})}^{(-1)}$ (as each individual *id* + *ϵ*_*n*_*u*_*n*_ is shown to have an inverse^69^). The elements of this subgroup can be thought of as diffeomorphisms arising from time-varying continuously differentiable flows.

However, the rate of convergence of these algorithms are contingent on the severity of ill-conditioning of (1). In subsequent sections, we first show the extent of ill-conditioning for diffeomorphic registration which subsequently warrants adaptive optimization over this subgroup of diffeomorphisms.

### Deformable image registration is a severely ill-conditioned problem

The ill-conditioned nature of image registration represents a comparatively neglected domain of inquiry within the extant literature. Recent works in the literature^19,62^ only speculate the ill-conditioned nature of registration but do not quantify it. Computing the ill-conditioning requires us to analyze the Hessian of the registration cost function. This is infeasible in general due to the high dimensionality of the problem; the full Hessian of a MRI brain registration problem requires more than 15 petabytes of memory to store. However, we consider a typical scenario of T1-weighted 3D MRI image registration with the L2 loss^15,16,34^: i.e. $C({I}_{f},{I}_{m},\varphi )={\sum }_{i}{({I}_{f}({{{{\bf{x}}}}}_{i})-{I}_{m}(\varphi ({{{{\bf{x}}}}}_{i})))}^{2}$. In this case, the gradient of *C* w.r.t. *φ*(**x**_*i*_) is (*I*_*m*_(*φ*(**x**_*i*_)) − *I*_*f*_(**x**_*i*_)) ∇ *I*_*m*_(*φ*(**x**_*i*_)), which does not depend on *φ*(**x**_*j*_), *j* ≠ *i*. Therefore, the full Hessian is simply a block-diagonal matrix containing pixelwise Hessians ${H}_{i}={\nabla }_{\varphi ({{{{\bf{x}}}}}_{i})}^{2}C$ with eigenvalues {*λ*_*i*_; *i* = {1, 2, 3}}. This makes the conditioning analysis tractable. We calculate the per-pixel condition number, defined as ${\kappa }_{i}=| {\lambda }_{i}^{\,\max }| /| {\lambda }_{i}^{\min }|$; and investigate the relationship between the fraction of foreground pixels and *κ*_*i*_ across multiple spatial resolutions of the images. The study considers three downsampling factors: 1x (original resolution), 2x, and 4x, in accordance with existing multi-scale optimization techniques. Supplementary Fig. 1 shows that across all resolutions, more than 60% of foreground pixels have a condition number greater than 10. To elucidate the impact of poor conditioning on optimization, we construct a simplified example of an ill-conditioned two-dimensional convex optimization problem, detailed in Appendix D. Even with *κ* = 10, convergence slows down drastically for an ill-conditioned convex optimization problem. This indicates severe ill-conditioning of the registration problem, strongly motivating the need for first-order adaptive optimization.

### Adaptive optimization for diffeomorphisms

We provide a brief overview of the mathematical frameworks employed to optimize parameters that reside on Riemannian manifolds like diffeomorphisms, followed by an algorithm that exploits the group action to define a gradient descent algorithm that eliminates computationally expensive steps. This formulation of the ‘gradient descent’ algorithm can then be formulated to incorporate adaptive algorithms such as Adam^28^ to optimize diffeomorphisms.

#### Euclidean gradient descent using the Lie algebra in shooting methods

Each Lie group has a corresponding Lie algebra ${\mathfrak{g}}$ which is the tangent space at identity. This creates a locally one-to-one correspondence between elements of the group *g* ∈ *G* and elements of its Lie algebra $v\in {\mathfrak{g}}$ given by the exponential map $\exp :{\mathfrak{g}}\to G;$ effectively to reach $g=\exp (v)\,{{{\rm{id}}}}$ from identity id ∈ *G*, the exponential map dictates that the group element has to move along *v* for unit time along the manifold. Exponential maps for many groups can be computed analytically, e.g., Rodrigues transformation for rotations, Jordan-Chevalley decomposition^70^, or the Cayley Hamilton theorem^71^ for matrices. For diffeomorphisms, the Lie algebra is the space of all smooth velocity fields $v:\Omega \to {{\mathbb{R}}}^{d}$. There exist iterative methods to approximate the exponential map called the scaling-and-squaring approach^17,34^ which uses the identity

3 $$\varphi=\exp (v)={\lim}_{N\to \infty }{\left({{{\rm{id}}}}+\frac{v}{N}\right)}^{N}$$

to define a recursion by choosing *N* to be a large power of 2, i.e. *N* = 2^*M*^ as

4 $${\varphi }^{(1/{2}^{M})}=id+v/{2}^{M}$$

5 $${\varphi }^{(1/{2}^{k})}={\varphi }^{(1/{2}^{(k+1)})}\circ {\varphi }^{(1/{2}^{(k+1)})}\,\,\forall k\in \{0,1\ldots,M-1\};$$

This can be thought of as a special case of (2) with $n={2}^{M},\epsilon=\frac{1}{n}$ and *u*_1_ = … = *u*_*n*_ = *v*.

By virtue of the exponential map, we can solve the registration problem of finding *φ* ∈ *G* by directly optimizing over the Lie algebra *v*. This is because the Lie algebra is a vector space and we can perform, for example, standard Euclidean gradient descent for registration^72–74^. Such methods are called stationary velocity field or shooting methods. At each iteration, one uses the exponential map to get the transformation *φ* from the velocity field *v*, computes the gradient of the registration objective with respect to *φ*, pulls back this gradient into the tangent space where *v* lies

$${\nabla }_{v}L=\frac{\partial \varphi }{\partial v}{\nabla }_{\varphi }L$$

and finally makes an update to *v*. Traditional methods like DARTEL^17^ implement this approach. This is also very commonly used by deep learning methods for registration^34,75,76^ due to its simplicity. Geodesic shooting methods are more sophisticated implementations of this approach where *φ* is the solution of a time-dependent velocity that follows the geodesic equation; the geodesic is completely determined by the initial velocity ${v}_{0}\in {\mathfrak{g}}$.

Adaptive optimization algorithms can be applied to the Lie algebra since it a Euclidean vector space ${\mathfrak{g}}$. However, there are a number of challenges with this method. First, this method requires computing the exponential map and its derivative, both of which need to be iteratively evaluated at each step of gradient descent. This is evident in Fig. 2 where direct optimization with ANTs runs much faster than the Lie-algebra counterpart. Moreover, the exponential map is only *locally* diffeomorphic, meaning it is suitable for modeling deviations close to the identity but not for large deformations—this leads to less expressivity and poor performance. In Fig. 2, the greedy SyN method which employs direct optimization significantly outperforms the Lie algebra-based DARTEL. In Supplementary Fig. 12, we observed that across a large variety of hyper-parameters evaluated via grid search, direct optimization consistently led to better target overlap compared to its Lie algebra counterpart on the LPBA40 dataset. Therefore, we do not consider this method in our work.

#### Riemannian gradient descent

Solving the registration problem directly on the space of diffeomorphisms avoids repeated computations to and fro via the exponential map. The downside however is that one now has to explicitly account for the curvature and tangent spaces of the manifold. The updates for Riemannian gradient descent^77^ at the *t*th iteration are

6 $${\varphi }_{t+1}=	 {\exp }_{{\varphi }_{t}}\left(-\eta \,{{{{\rm{Proj}}}}}_{{\varphi }_{t}}({\nabla }_{\varphi }L)\right)\\ \,{{{\rm{where}}}}\,{\nabla }_{\varphi }L=	 {{{{\bf{g}}}}}_{{\varphi }_{t}}^{-1}\frac{\partial L}{\partial \varphi },$$

where one pulls back the Euclidean gradient $\frac{\partial L}{\partial \varphi }$ onto the manifold using the inverse metric tensor **g** (which makes the gradient invariant to the parameterization of the manifold of diffeomorphisms) before projecting it to the tangent space using ${{{{\rm{Proj}}}}}_{{\varphi }_{t}}$. Since the tangent space is a local first-order approximation of the manifold’s surface, we can move along this descent direction by a step-size *η* and compute the updated diffeomorphism *φ*_*t*+1_, represented as the exponential map from *φ*_*t*_ computed in the direction of $-{{{{\rm{Proj}}}}}_{{\varphi }_{t}}({\nabla }_{\varphi }L)$.

However, there are a few challenges in optimizing diffeomorphisms using Riemannian gradient descent. First, adaptive optimization algorithms such as RMSProp^27^, Adagrad^78^ and Adam^28^ have become popular because they can handle poorly conditioned optimization problems in deep learning. Variants for optimization on low-dimensional Riemannian manifold exist^79–82^. In contrast to these manifolds, diffeomorphisms are a high-dimensional variable-sized group (e.g., the parameterization of the warp field scales with that of the image size). Therefore, operations like computing the Riemannian metric tensor, and parallel transport of the optimization state variables (momentum and curvature) are very computationally expensive. For diffeomorphisms, computing the parallel transport requires solving a system of partial differential equations, which is computationally expensive. For these reasons, we do not consider direct Riemannian optimization for diffeomorphisms in our work.

### Exploiting the group structure of diffeomorphisms

Diffeomorphisms are imbued with additional structure compared to a Riemannian manifold—they are a Lie group as well. Not all Riemannian manifolds are Lie groups—notable examples of non-Lie group Riemannian manifolds include the sphere ${{\mathbb{S}}}^{n}$, fixed-rank matrices, and the Stiefel and Oblique manifolds^77^. The additional Lie group structure of $G(\Omega,{{\mathbb{R}}}^{d})$ allows us to exploit the group action to define a gradient descent algorithm that eliminates computationally expensive steps. In the following text, we provide a method to compute a descent direction in the group of diffeomorphisms that is computationally efficient and can be used with adaptive optimization algorithms.

#### Minimizing the Eulerian differential

Consider a function $U:G\to {\mathbb{R}}$ that we aim to minimize. Let *V* be an admissible Hilbert space of vector fields on *Ω* embedded in ${C}_{0}^{1}(\Omega,{{\mathbb{R}}}^{d})$. We define an Eulerian differential in *V* if there exists a linear form $\partial \bar{U}\in {V}^{*}$ such that for all *v* ∈ *V*:

7 $${\left(\bar{\partial }U(\varphi )| v\right)}_{E}={\left.{\partial }_{t}U(\varphi \circ {\varphi }_{0t}^{v})\right|}_{t=0}$$

and ${\varphi }_{0t}^{v}(x)={\exp }_{id}(tv)(x)=x+{\int }_{0}^{t}v({\varphi }_{0s}^{v}(x))ds$ is the flow of the vector field *v* starting from the identity. This definition of Eulerian differential is different from the one in ref. ^70^ to perform all updates (*v*) in the tangent space at identity and leverage Jacobian-free descent (see later). The goal is to choose a suitable *v* such that the directional change of the Eulerian differential along *v* is negative, making *v* a descent direction. A more familiar rate of change of *U* along a curve *v* is given by the *Gateaux derivative*:

8 $${\left.\left(\frac{\partial U}{\partial \varphi }\right|v\right)}_{G}={\left.{\partial }_{t}U(\varphi+tv)\right|}_{t=0}$$

The Eulerian differential is closely related to the Gateaux derivative of *U* at *φ* as:

$${\left(\bar{\partial }U(\varphi )| v\right)}_{E}={\left.\left(\frac{\partial U}{\partial \varphi }\right| \, J(\varphi )v\right)}_{G}$$

using chain rule. The right side is further expanded as:

$${\left(\bar{\partial }U(\varphi )| v\right)}_{E}=\int _{\Omega }{\left(\frac{\partial U}{\partial \varphi }(\varphi )(x)\right)}^{\top }J(\varphi (x))v(x)dx$$

where *J*(*φ*)(*x*) = *J*(*φ*(*x*)) with slight abuse of notation. We introduce the Gateaux derivative and relate it to the Eulerian derivative because we typically have access to the Gateaux derivative using automatic differentiation tools like PyTorch, but to perform optimization on the group of diffeomorphisms, we need to compute the Eulerian differential. Choosing

$${v}_{d}(x)=-J{(\varphi (x))}^{\top }\,\frac{\partial U}{\partial \varphi }(\varphi )(x)$$

gives us:

$${\left(\bar{\partial }U(\varphi )| {v}_{d}\right)}_{E}=- \int _{\Omega }{\left\Vert J{(\varphi )}^{\top }\frac{\partial U}{\partial \varphi }(\varphi )(x)\right\Vert }^{2}dx < 0$$

This choice of *v*_*d*_(*x*) is therefore a descent direction for the Eulerian differential of *U* at *φ*. To perform gradient descent on the Eulerian differential at *φ*, we need to compute the descent direction *v*_*d*_, perform the exponential map with a small learning rate *η*_*t*_, and perform the update:

$${\varphi }_{t+1}={\varphi }_{t}\circ {\exp }_{id}({\eta }_{t}{v}_{d})$$

For small enough *η*_*t*_, the exponential map can be approximated with a retraction map (i.e. ${\exp }_{id}({\eta }_{t}{v}_{d})\approx id+{\eta }_{t}{v}_{d}$), which is quick to compute.

We quickly contextualize the key differences between Gateaux gradient descent and our proposed Eulerian descent. First, the steepest descent direction in Gateaux gradient descent is $-\frac{\partial U}{\partial \varphi }(\varphi )$, whereas it is $-J{(\varphi )}^{\top }\frac{\partial U}{\partial \varphi }(\varphi )$ in Eulerian descent. Second, the update rule in Gateaux gradient descent is ${\varphi }_{t+1}={\varphi }_{t}-{\eta }_{t}\frac{\partial U}{\partial \varphi }(\varphi )$, whereas it is ${\varphi }_{t+1}={\varphi }_{t}\circ {\exp }_{id}({\eta }_{t}{v}_{d})$ in Eulerian descent. These two differences capture the essence of performing optimization on the group of diffeomorphisms in contrast to optimizing on the (Euclidean) ambient space directly.

#### Adaptive optimization on diffeomorphisms

Note that for small enough *t*, the descent direction *v*_*d*_(*x*) can also be interpreted as a vector in the tangent space at identity, with ${\varphi }_{0t}^{v}={\exp }_{id}(tv)$ since ${\varphi }_{00}^{v}=id$, and ${\partial }_{t}{\varphi }_{0t}^{v}{| }_{t=0}=v$. Descent directions over gradient descent iterations *i* denoted as ${v}_{d}^{(i)}$ all lie on the same vector space, i.e. the tangent space at identity. Therefore, first order algorithms like Adam can be applied on the sequence of descent directions ${v}_{d}^{(i)}$ which now lie in the same vector space, without requiring computing the metric tensor, parallel transport or change of coordinates (charts) throughout the optimization process. This framework leveraging the group structure forms the core of our adaptive optimization algorithm for diffeomorphisms. Our framework is therefore a significant advantage over Riemannian optimization methods which require parallel transport of the momentum and curvature vectors at each iteration.

#### Jacobian-free Eulerian descent

We provided an obvious choice of descent direction *v*_*d*_(*x*) for the Eulerian differential of *U* at *φ*. The Gateaux derivative $\frac{\partial U}{\partial \varphi }$ is readily obtained using automatic differentiation tools like PyTorch. However, the descent direction requires us to multiply this derivative with the Jacobian of the diffeomorphism *J*(*φ*), which may be computationally expensive. However, in most diffeomorphic image registration applications, the role of the diffeomorphism is warp the image by performing local translations, scaling and shearing without introducing large local rotations. Mathematically, we consider the polar form of the Jacobian *J*(*φ*)(*x*) = *U*(*x*)*P*(*x*) where *U*(*x*) is a unitary matrix, and *P*(*x*) is a positive definite matrix. We assume that for most applications, *U*(*x*) ≈ *I*_*d*×*d*_, making *J*(*φ*)(*x*) positive definite. With this assumption, we can choose the modified descent direction

$$v^{\prime}_{d} (x)=-\frac{\partial U}{\partial \varphi }(\varphi )(x)$$

and the Eulerian differential at *φ* is

$${\left(\bar{\partial }U| v{\prime} \right)}_{E}=- \int _{\Omega }{\left(\frac{\partial U}{\partial \varphi }(\varphi )(x)\right)}^{\top }J(\varphi (x))\frac{\partial U}{\partial \varphi }(\varphi )(x)dx < 0$$

since $v{\prime} {(x)}^{\top }J(\varphi (x))v{\prime} (x)\ge 0$ for all *x* ∈ Ω, owing to the (assumed) positive definiteness of *J*(*φ*)(*x*). For all experiments, Jacobian-free descent directions $v{\prime} (x)$ are used, and they provide faster runtime and with same accuracy. Adaptive first-order optimization can now be performed on this modified sequence on descent directions $v{{\prime} }^{(i)}(x)$, saving significant computational and memory overhead by avoiding computation of *J*(*φ*).

Note that this algorithm using the Eulerian differential is only possible due to the group structure of diffeomorphisms. For an arbitrary Riemannian manifold ${{{\mathcal{M}}}}$ and points $\varphi,{\varphi }_{0t}^{v}\in {{{\mathcal{M}}}}$, the operation $\varphi \circ {\varphi }_{0t}^{v}$ does not make sense. The additional group structure of $G(\Omega,{{\mathbb{R}}}^{d})$ allows us to propose an Eulerian descent algorithm without performing Lie algebra optimization, or Riemannian gradient descent, both of which are computationally expensive for diffeomorphisms.

#### Alternative formulations for Eulerian differential

Our definition of (7) is different from the one in ref. ^70^ in two subtle but important ways. First, we do not define the registration objective in terms of the group action or pullback image *φ*. *I* = *I*(*φ*^−1^(*x*)), and instead define the objective in terms of the pushforward image *I*(*φ*(*x*)). This is to avoid computing and storing both *φ* for autodifferentiation and *φ*^−1^ for computing the objective, implementational simplicity, and consistency with more modern registration framework formulation. FireANTs provides additional functionality to compute *φ*^−1^*post hoc* using a multi-scale objective function similar to the image matching objective: ${\varphi }^{-1}={{{{\rm{argmin}}}}}_{\psi \in G}{\sum }_{x\in \Omega }\parallel \psi (\varphi (x))-x{\parallel }_{2}^{2}+\parallel \varphi (\psi (x))-x{\parallel }_{2}^{2}$. This allows researchers to obtain an inverse transform on a *post hoc* basis without computing *φ*^−1^*during* optimization. This subroutine is also used in the symmetric registration objective to compute the final transformation $\varphi={\varphi }_{M}\circ {\varphi }_{F}^{-1}$. The second difference is the definition of the Eulerian differential itself - note that we use the composition $U(\varphi \circ {\varphi }_{0t}^{v})$ in (7) instead of $U({\varphi }_{0t}^{v}\circ \varphi )$. Defining the Eulerian differential using the second formulation without using the group action *φ*. *I* implies that we will compute the velocity field in the Lagrangian frame, i.e. $V(y)=v(\varphi (x))=-\frac{\partial U}{\partial \varphi }$. To compute adaptive optimization updates and Lipschitz constant to scale the learning rate (see Appendix E), we need to compute the corresponding velocity field in the Eulerian frame, i.e. *v*(*x*) = *V*(*φ*^−1^(*y*)) which requires computing *φ*^−1^. Therefore, we choose a definition that avoids computing *φ*^−1^ and allows inexpensive adaptive optimization updates.

### Interpolation strategies for multi-scale registration

Classical approaches to deformable image registration is performed in a multi-scale manner. Specifically, an image pyramid is constructed from the fixed and moving images by downsampling them at different scales, usually in increasing powers of two. Optimization is performed at the coarsest scale first, and the resulting transformation at each level is used to initialize the optimization at the next finer scale. Specifically, for the fixed image *I* and the moving image $I{\prime}$ and *K* levels, let the downsampled versions be ${\{{I}_{k}\}}_{k=1}^{K}$ and ${\{I{{\prime} }_{k}\}}_{k=1}^{K}$, where *k* is the scale index from coarsest to finest. At the *k*-th scale, the transformation *φ*_*k*_ is optimized as:

$${\varphi }_{k}^{*}={{{{\rm{argmin}}}}}_{{\varphi }_{k}\in G}L({I}_{k},I{{\prime} }_{k}\circ {\varphi }_{k})$$

where *φ*_*k*_ is initialized as:

$${\varphi }_{k}=\left\{\begin{array}{ll}id \hfill & \,{{{\rm{if}}}}\,k=1 \hfill \\ \,{{{\rm{Upsample}}}}\,({\varphi }_{k-1}) & \,{{{\rm{otherwise}}}}\end{array}\right.$$

Unlike existing gradient descent based approaches, our Riemannian adaptive optimizer also contains state variables *m*_*k*_ corresponding to the momentum and *ν*_*k*_ corresponding to the EMA of squared gradient, at the same scale as *φ*_*k*_, which require upsampling as well.

Unlike upsampling images, upsampling warp fields and their corresponding optimizer state variables requires careful consideration of the interpolation strategy. Bicubic interpolation is a commonly used strategy for upsampling images to preserve smoothness and avoid aliasing. However, bicubic interpolation of the warp field can lead to overshooting, leading to introducing singularities in the upsampled displacement field when there existed none in the original displacement field. In contrast, bilinear or trilinear interpolation does not lead to overshooting, and therefore diffeomorphism of the upsampled displacement is guaranteed, if the original displacement is diffeomorphic. We demonstrate this using a simple 2D warp field in Supplementary Fig. 9b. On the left, we consider a warp field created by nonlinear shear forces. This warp field does not contain any tears or folds—and is diffeomorphic. We upsample this warp field using bicubic interpolation (top) and bilinear interpolation (bottom). We also plot a heatmap of the negative of the determinant of the Jacobian of the upsampled warp, with a white contour representing the zero level set. Qualitatively, bicubic interpolation introduces noticeable folds in the warping field, leading to non-diffeomorphisms in the upsampled warp field. The heatmap shows a significant portion of the upsampled warp field has a negative determinant, indicating non-invertibility. On the other hand, bilinear interpolation looks blocky but preserves diffeomorphism everywhere, as also quantitatively verified by the absence of a zero level set in the heatmap. The complete algorithm is described in Algorithm 1.

#### Algorithm 1

**Algorithm for FireANTs** We outline the key steps in FireANTs—computing the Jacobian-free Eulerian descent direction which is simply the Gateaux derivative. If the boolean use_jac is specified, then use the steepest Eulerian descent direction instead. This descent direction is then modified using any adaptive optimization algorithm denoted as optstate. The warp field is then updated using the exponential map or retraction map for small *ϵ*_*i*_. After optimization at a given scale, the warp field is upsampled using bilinear or trilinear interpolation to the next scale until optimization is complete for all steps.

1: **Input:** Fixed image *I*_*f*_, Moving image *I*_*m*_

2: Scales [*s*_1_, *s*_2_, …, *s*_*n*_], Iterations [*T*_1_, *T*_2_, …*T*_*n*_], *n* scales

3: optstate optimizer state (for Adam, RMSProp, etc.)

4: use_jac boolean specifying whether to use Jacobian in descent direction

5:

6: Initialize $\varphi \leftarrow {{{{\bf{id}}}}}_{{s}_{1}}$.           ⊳ Initialize warp to identity at first scale

7: Initialize *l* ← 1.   ⊳ Initialize current scale

8: **while** *l*≤*n* **do**

9:  Initialize *i* ← 0

10:    Initialize $${I}_{f}^{l},{I}_{m}^{l}\leftarrow \,{{{\rm{downsample}}}}({I}_{f},{s}_{l}),{{{\rm{downsample}}}}\,({I}_{m},{s}_{l})$$

11:  **while** *i* < *T*_*l*_ **do**

12:   $${U}_{i}\leftarrow C({I}_{f}^{l},{I}_{m}^{l}\circ {\varphi }^{i})+R(\varphi )$$

13:     Compute $v{\prime} (x)\leftarrow \frac{\partial {U}_{i}}{\partial \varphi }({\varphi }^{(i)})(x)$   ⊳ Jacobian-free Eulerian descent direction

14:      **if** use_jac **then**

15:    Compute $v{\prime} (x)\leftarrow {J}^{\top }({\varphi }^{(i)}(x))v{\prime} (x)$   ⊳ Eulerian descent direction

16:      **end if**

17:    Update $(v{\prime} (x),\,{\mathtt{optstate}})\leftarrow {\mathtt{optstate}}\,(v{\prime} (x))$      ⊳ Apply and update optimizer state

18:      Update $${\varphi }^{(i+1)}\leftarrow {\varphi }^{(i)}\circ {\exp }_{id}({\epsilon }_{i}v{\prime} )\approx {\varphi }^{(i)}\circ (id+{\epsilon }_{i}v{\prime} )$$

19:   *i* ← *i* + 1

20:    **end while**

21:     **if** *l* < *n* **then**

22:    *φ* ← Upsample(*φ*, *s*_(*l*+1)_)      ⊳ Upsample warp to next scale using bilinear/trilinear interpolation

23:    **end if**

24:    *l* ← *l* + 1

25: **end while**

### Reporting summary

Further information on research design is available in the Nature Portfolio Reporting Summary linked to this article.

## Supplementary information

Supplementary Information

Reporting Summary

Transparent Peer Review file

## Source data

Source Data

## Data Availability

The following datasets were used in the study and are publicly available for free: Klein et al. neuromapping challenge datasets (IBSR18, CUMC12, MGH10, LPBA40): https://www.synapse.org/#!Synapse:syn3251018; OASIS, NLST, Abdomen MRCT registration datasets: https://learn2reg.grand-challenge.org/Datasets/; EMPIRE10 lung dataset: https://empire10.grand-challenge.org/Download/; RnR Expansion Microscopy mouse dataset: https://rnr-exm.grand-challenge.org/data/; Fluorescence micro-optical sectioning tomography (fMOST) imaging for mouse brain data: https://knowledge.brain-map.org/data/K1YP17A0QIKJOMOAIS4; PRIME-DE Macaque dataset: https://fcon_1000.projects.nitrc.org/indi/indiPRIME.html; Ultracortex dataset: https://openneuro.org/datasets/ds005216/versions/1.1.0/download; Waxholm Rat Brain dataset: https://www.nitrc.org/frs/?group_id=1081#; Allen CCFv3 mouse brain dataset: https://atlas.brain-map.org/atlas; AZBA Zebrafish dataset: https://azba.wayne.edu/; ZBrains Zebrafish dataset: https://zebrafishexplorer.zib.de/ Source data are provided with this paper.

## Code availability

The code for FireANTs is publicly available at https://github.com/rohitrango/fireants^83–96^.
