# Supplementary material for: Adaptive Riemannian optimization for multi-scale diffeomorphic matching
Source: Nat Commun. 2026 Jun 9;17:4774. doi: 10.1038/s41467-026-72508-3 (PMC13249883; doi:10.1038/s41467-026-72508-3)
Supplement: Supplementary file 1 — Supplementary Information [file 41467_2026_72508_MOESM1_ESM.pdf]

Supplementary Material

A Related Work

The field of image registration has evolved through several distinct phases, introducing progressively more expressive mathematical and computational frameworks. Early work in computational anatomy established the foundational view of registration as the problem of mapping anatomical variability through smooth, invertible transformations between coordinate systems. Pioneering contributions by Grenander and colleagues formalized anatomical shapes as elements of a deformable template space, leading to probabilistic formulations of anatomical variability and population analysis [1]. These works introduced the notion that anatomical differences could be quantified through the geometry of transformations rather than through intensity differences alone, thereby providing the mathematical basis for subsequent developments in diffeomorphic mapping. Other works model diffeomorphisms as solutions of elastic matching [2,3] and large deformation kinematics [4,5]. To mitigate some of the limitations of these approaches, the Large Deformation Diffeomorphic Metric Mapping (LDDMM) framework [6] provided a rigorous variational and geometric formulation of registration. In LDDMM, deformations are modeled as flows of smooth velocity fields generating geodesics on the diffeomorphism group under a right-invariant metric. Subsequent extensions introduced more efficient parameterizations and regularizers, including stationary velocity fields [7], shooting formulations [8], and Riemannian approaches to statistical analysis on the manifold of diffeomorphisms [9]. A complementary approach to ensure smooth and anatomically plausible deformations is to supplement the image similarity objective with a regularization function that is applied on the displacement field. Typical examples include minimizing the bending energy [10,11], total variation [12], diffusion regularizer [13,14,15] that aim to constrain the degrees of freedom of the deformation field. Regularization can also be implicit - different deep network architectures induce different implicit regularizations [16], and choice of representation of the nonlinear transform (stationary velocity field, B-Spline, downsampled displacement fields) constrains the solution space, providing implicit regularization [11,17]. These advances in representation and regularization links registration to broader concepts in geometric mechanics and fluid dynamics, providing both theoretical interpretability and a pathway for optimization-based implementation. Subsequent gradient-descent based optimization formulations [18,19] led to the development of the Advanced Normalization Tools (ANTs) [20], which is a state-of-the-art registration framework for medical image registration. ANTs is used routinely across a broad range of biomedical and life sciences research workflows, and is the de-facto standard for image registration. However, ANTs uses a simple gradient-descent based approach instead of utilizing powerful and efficient adaptive optimization algorithms, and is slow and not scalable due to a CPU-only implementation, and does not leverage GPUs to exploit the massively parallelizable nature of the problem.

More recently, the field has shifted toward model-based deep learning approaches that approximate the optimization solution with feedforward inference for inference speed. Methods such as VoxelMorph [14] and subsequent unsupervised learning based variants [21,22,23] learn parametric mappings that approximate solutions to variational registration problems. However, most methods cannot represent solutions to time-dependent velocity fields, therefore most deep learning methods rely on stationary velocity field representations to model Lie group of diffeomorphisms using its Lie algebra. However, stationary velocity fields have a few computational and mathematical limitations, outlined in §E. Another limitation of deep networks is the large activation memory overhead for inference, making them infeasible for high-resolution registration. Other

works[24,25,26] use approximations like bandlimited velocity fields to model efficient inference, at the cost of potentially losing some of the high-frequency details in the velocity field that is potentially useful for modelling cortical folding patterns, or large atrophy of ventricles in diseased patients. Moreover, the memory overhead and scalability of these methods to large datasets is not well studied. Another limitation of these methods is their drop in performance on out-of-distribution datasets[27,28,29,30,31], that has motivated the need for domain-agnostic or foundational models[30,31]. Other works[32] also claim generalization due to architectural design choices that mimic registration specific operations. In our experiment setup, we compare our method with these domain-agnostic methods to test the true long tail generalization capabilities of domain-agnostic registration algorithms. We wish to preserve the full expressivity of time-depdendent velocity fields for modelling diffeomorphisms, and enable adaptive optimization on this space directly, while maintaining high computational efficiency and low memory overhead for inference. This motivates revisiting the classical optimization-based method ANTs, and extending it to enable powerful adaptive optimization for improved accuracy and convergence while improving computational efficiency and scalability using a GPU implementation.

B Datasets and Evaluation Metrics

We provide details about the datasets and evaluation metrics used in the paper.

B.1 In-vivo brain MRI mapping challenges (Klein *et al.*and OASIS)

Klein *et al.*neuromapping challenge Brain image data and their corresponding labels for 80 normal subjects were acquired from four different datasets. The *LPBA40* dataset contains 40 brain images and their labels to construct the LONI Probabilistic Brain Atlas (LPBA40). All volumes were skull-stripped, and aligned to the MNI305 atlas [33] using rigid-body transformation to correct for head tilt. For all these subjects, 56 structures were manually labelled and bias-corrected using the BrainSuite software. The *IBSR18* dataset contains brain images acquired at different laboraties through the Internet Brain Segmentation Repository. The T1-weighted images were rotated to be in Talairach alignment and bias-corrected. Manual labelling is performed resulting in 84 labeled regions. For the *CUMC12* dataset, 12 subjects were scanned at Columbia University Medical Center on a 1.5T GE scanner. Images were resliced, rotated, segmented and manually labeled, leading to 128 labeled regions. Finally, the *MGH10* dataset contains 10 subjects who were scanned at the MGH/MIT/HMS Athinoula A. Martinos Center using a 3T Siemens scanner. The data is bias-corrected, affine-registered to the MNI152 template, and segmented. Finally the images were manually labeled, leading to 74 labeled regions. All datasets have a volume of $256 \times 256 \times \{128, 124\}$ voxels with varying amounts of anisotropic voxel spacing, ranging from $0.84 \times 0.84 \times 1.5$mm to $1 \times 1 \times 1.33$mm. ANTs was one of the top performing methods for this challenge, performing well robustly across all four datasets.

A natural way to evaluate whether two images are in a common coordinate frame is to evaluate the accuracy of overlap of gross morphological anatomical structures. The method considers measures of volume and surface overlap, volume similarity, and distance measures to evaluate the alignment of anatomical regions. Given a source label map S_r and target label map T_r and a cardinality operator $|.|$, we consider the following overlap measures. We consider 'target overlap' and 'mean overlap' (also known as Dice score) as the primary measures of agreement between

the source and target label maps.

$$TO_r = \frac{|S_r \cap T_r|}{|T_r|}, MO_r = 2\frac{|S_r \cap T_r|}{|S_r| + |T_r|} \tag{4}$$

The aggregates over all regions are given by:

$$TO = \frac{1}{N_r}\sum_r TO_r \quad , \quad MO = \frac{1}{N_r}\sum_r MO_r \tag{5}$$

Klein *et al.*[34] also propose a 'Union Overlap' metric which is a monotonic function of the Dice score. Therefore, we do not use this in our evaluation. To complement the above agreement measures, we also compute false negatives (FN), false positives (FP), and volume similarity (VS) coefficient for anatomical region r:

$$FN_r = \frac{|T_r \backslash S_r|}{|T_r|}, \quad FP_r = \frac{|S_r \backslash T_r|}{|S_r|}, \quad VS_r = 2\frac{|S_r| - |T_r|}{|S_r| + |T_r|} \tag{6}$$

Comparison on other metrics proposed in [34] and regionwise analysis are shown in Supplementary Fig. 8 and Supplementary Fig. 7. Similar to the overlap metrics, we compute the aggregates as in the original evaluation denoted by $FN_{Klein}, FP_{Klein}, VS_{Klein}$ and average over regions denoted simply by FN, FP, VS.

OASIS dataset On the OASIS dataset, we use same the evaluation criteria as in the Learn2Reg challenge[35], i.e. Dice score overlap and 95th percentile of the Haussdorf distance computed for 35 subcortical structures. This leads to a total of 12 evaluation metrics that we use to compare our method with 4 baselines - ANTs, Demons[36], VoxelMorph[14] and SynthMorph[30], representing established classical and deep learning registration algorithms.

In total, we compare with state-of-the-art baselines on over *2000 brain volume pairs*, with varying number of labeled anatomical regions and resolutions.

B.2 Lung CT mapping challenges (EMPIRE10 and NLST)

Registration of temporally spaced breathhold scans can help in tracking disease progression, or registration between inspirion and expiration scans can enable improved monitoring of airflow and pulmonary function. The EMPIRE10 challenge [37] aims to provide a platform for in-depth evaluation and fair comparison of available registration algorithms for this application. The dataset consists of 30 pairs of chest CT scans, with intra-subject registration across a variety of healthy or diseased subjects. The scan pairs consist of inspiration-expiration scans, breathhold scans over time, scans from 4D data, ovine data, contrast-noncontrast, and artificially warped scan pairs. The ovine data was acquired where breathing was controlled, and metallic markers were surgically implanted to provide landmark annotations, followed by a hole-filling algorithm to disguise the markers so that registration algorithms cannot use this artificial information. Artificially warped scan pairs also provide ground truth correspondences for landmarks and lung boundaries. The challenge provides a broad range of data complexity, voxel sizes and image acquisition differences. ANTs is, again one of the top performing methods in this challenge. Unlike the brain datasets, ground truth labels for fissure and landmarks are not provided for validation. Therefore, we rely on the evaluation metrics computed privately by the challenge organizers in the evaluation server. We compare our method with two powerful baselines (i) ANTs, which optimizes the diffeomorphism directly, and (ii) the *DARTEL*[7] formulation optimizing a stationary velocity field (SVF), where the diffeomorphism is obtained using an exponential map

of the SVF. We first affinely align the binary lung masks of the moving and fixed images using Dice loss[38]. This is followed by a diffeomorphic registration using the intensity images.

We use the Adam optimizer with learning rate of 3e-3, and a multi-scale optimization with downsampling rates of 6,4,2,1 for 200, 100, 50, 20 iterations. This is followed by a diffeomorphic registration step with the same multi-scale resolutions and 200,150,75,25 iterations and a learning rate of 0.25. We use a Gaussian kernel for gradient smoothing with $\sigma_{\text{grad}} = 6.0$ and warp smoothing with $\sigma_{\text{warp}} = 0.4$. The optimal values for $\sigma_{\text{grad}}, \sigma_{\text{warp}}$ are found by a hyperparameter grid search, and are strikingly close to the parameters used in the ANTs submission.

We evaluate three criteria: (1) fissure alignment errors (%)—the fraction of misaligned fissure voxels (Figs. 2b and 2e), (2) landmark distance in mm (Fig. 2d), and (3) singularity errors—the fraction of non-diffeomorphic voxels (Fig. 2c). Fig. 2 highlights the impact of representation choice in modeling diffeomorphisms. DARTEL, using an exponential map, performs significantly worse than ANTs across all metrics by three orders of magnitude. In contrast, our method reduces fissure alignment error by 5$\times$ compared to ANTs and outperforms it in 5 out of 6 landmark subregions. While all methods theoretically ensure diffeomorphism, SVF-based approaches introduce singularity errors due to non-adaptive scaling-and-squaring. We discuss the limitations of SVF-based approaches in §E. ANTs also introduces some singularities, whereas our method computes numerically perfect diffeomorphic transforms. Finally, Fig. 2e compares fissure alignment errors among EMPIRE10 submissions, showing FireANTs achieves the lowest landmark errors and the fastest runtime among the top 10 methods, setting new benchmarks in computational efficiency and accuracy. Our method, on the other hand computes numerically perfect diffeomorphic transforms. Finally, we compare the fissure alignment error of all submissions in the EMPIRE10 challenge, and show the top 10 algorithms in Fig. 2e. Results demonstrate that FireANTs attains the lowest landmark alignment errors compared to an array of contemporary state-of-the-art algorithms.

NLST dataset For the NLST dataset[39], we compare with representative state-of-the-art optimization and deep-learning baselines. We use the evaluation criteria provided by the challenge, and measure results on the Robust Target Registration Error (TRE30) in millimeters between the registered keypoints. Results in Fig. 2f show that FireANTs outperforms all baselines on the NLST dataset, with improvements of upto 51.6% in robust target registration error (TRE30) of provided keypoints compared to state-of-the-art deep learning benchmarks including Im2Grid, Vector-Field Attention, RWC-Net, and a 50.8% improvement in TRE30 over foundation models like unigradICON. This demonstrates the broad applicability of FireANTs beyond neuroimaging applications.

B.3 Other Datasets and Metrics

PRIMatE Data Exchange (PRIME-DE) A growing body of research has documented the utility of MRI data to study neuroanatomical organization and function of non-human primates. The PRIMatE Data Exchange (PRIME-DE) resource[40] provides a platform for the neuroimaging community to facilitate the mapping of the non-human primate connectome. We use a subset of this dataset collected from five different sources: Aix-Marseille Université, Mount Sinai School of Medicine, McGill University, Stem Cell and Brain Research Institute, and the University of California, Davis, resulting in 116 subjects, and subsequently 13340 subject pairs for registration. We use the nBEST deep learning framework to perform cerebrum extraction, followed by tissue segmentation. Since the images are markedly different than human brains, we affinely register all of the extracted cerebrum volumes to the first subject sorted by name to bring them to a

common coordinate space and metadata. This is followed by a diffeomorphic registration using the intensity images. We use the Dice score of the registered tissue segmentations to evaluate the quality of registration.

Ultracortex The Ultracortex dataset[41] hosts a unique collection of ultra-high field (9.4 Tesla) MRI data of the human brain. This dataset includes detailed structural images and high-quality manual segmentations, making it an invaluable resource for researchers in neuroimaging and computational neuroscience. Out of the 86 T1-weighted images with resolutions spanning from 0.6 to 0.8mm, precise manual segmentation of the gray and white matter for each hemisphere is provided for 12 subjects. We use the dataset and manually provided segmentations to evaluate the quality of registration of cortical surface mapping using Dice score. Note that all deep learning methods run out of memory at 0.6 to 0.8mm resolutions, therefore we resample the images to 1.0mm isotropic resolution for evaluation of deep learning methods.

Rodent Datasets We use four rodent datasets in this study: Waxholm Rat Brain, Allen CCFv3 mouse brain, RnR-ExM mouse isocortex, and BICCN mouse dataset. The datasets feature high-resolution atlases of the rat and mouse brain with four different modalities (T2*w MRI, STPT, ExM, fMOST) respectively. The motivation for using the datasets is to provide a benchmark for *cross-species, multimodal* registration (Waxholm $\rightarrow$ Allen CCFv3), perform well on a high-resolution registration task and leaderboard (RnR-ExM), and to faithfully reproduce high-resolution rodent atlases (BICCN). Similar to the Ultracortex dataset, most deep learning methods run out of memory at 25μm resolution for these images, therefore we resample the images to 50μm resolution for evaluation of deep learning methods. To handle the multimodal nature of the cross-species registration task, we use Anatomix[42] as a modality-agnostic feature extractor as feature images to perform registration. Since the cross-species templates have different labelmaps, comparing Dice scores directly is not possible. The Waxholm template comprises 95 labeled regions, while the Allen CCFv3 template includes over 300 regions defined in the complete ARA ontology. To enable a comparable level of anatomical granularity, we coarsened the Allen CCFv3 parcellation to 34 regions by collapsing all subregions beyond depth 3 in the ontology hierarchy into their corresponding parent nodes. We compute the Mutual Information (MI) between the registered label map corresponding to the Waxholm template image to that of the Allen CCFv3 template image to evaluate the quality of registration. For the fMOST and RnR-ExM datasets, we use the images to perform pairwise registration and atlas generation respectively. The performance on the RnR-ExM dataset is evaluated using the Dice score of the registered label map corresponding to the ExM image pairs on a private evalaution server. For the BICCN dataset, we only provide qualitative results due to the lack of an evaluation criteria.

Zebrafish Datasets Analysis of the zebrafish is a growing field of research due to its unique advantages as a vertebrate model organism. The zebrafish brain is small yet structurally complex, offering a tractable system for studying whole-brain organization, development, and function at cellular resolution. High-quality atlases such as AZBA and Z-Brains provide detailed anatomical and gene expression reference templates, enabling cross-modality and cross-sample comparison. These datasets present a valuable testbed for registration algorithms, as they involve significant structural variability, diverse imaging modalities (including confocal, light-sheet, and two-photon microscopy), and finely detailed neuroanatomical annotations. Accurate registration in this setting is critical for integrating large-scale imaging data and mapping functional or genetic information onto common anatomical frameworks. The adult and larval zebrafish brains have very different

structural organization, and show very different characteristics than human brains. This is a challenging registration task to truly access the out-of-distribution generalization capabilities of registration algorithms. Due to the lack of a consensus on appropriate evaluation criteria beyond qualitative comparison for these datasets, we use the mutual information between the registered image and template image to evaluate the quality of registration.

Learn2Reg Abdomen MRCT registration This dataset is used as a testbed to ablate the effect of Jacobian-free optimization on abdominal MRCT registration. Abdomen CT-MR registration is a conceptually different registration task copmared to neuroanatomical or pulmonary registration with completely unrelated anatomical structures, organization, and biomechanical dynamics. We use the validation split provided by the challenge to evaluate the performance of FireANTs with and without Jacobian-free optimization.

C Modular software implementation to enable effective experimentation

Registration is a key part of many data processing pipelines in the clinical literature. Our software implementation is designed to be extremely flexible, e.g., it implements a number of existing registration methods using our techniques, modular, e.g., the user can choose different group representations (rigid or affine transforms, diffeomorphisms), objective functions, optimization algorithms, loss functions, and regularizers. Users can also stack the same class of transformations, but with different cost functions. For example, they can fit an affine transform using label maps and Dice loss, and use the resultant affine matrix as initialization to fit another affine transform using the cross-correlation registration objective. This enables seamless tinkering and real-time investigation of the data. Deformations can also be composed in increasing order of complexity (rigid $\rightarrow$ affine $\rightarrow$ diffeomorphisms), thereby avoiding multiple resampling and subsequent resampling artifacts. We have developed a simple interface to implement custom cost functions, which may be required for different problem domains, with ease; these custom cost functions can be used for any of the registration algorithms out-of-the-box. Our implementation can handle images of different sizes, anisotropic spacing, without the need for resampling into a consistent physical spacing or voxel sizes. All algorithms also support multi-scale optimization (even with fractional scales) and convergence monitors for early-stopping.

Our software is implemented completely using default primitives in PyTorch. All code and example scripts is available at https://github.com/rohitrango/fireants.

D On the Ill-conditioning of Image Registration

Image registration is a highly ill-conditioned, and non-convex problem necessitating advanced optimization methods for convergence. To provide more intuition on the effect of κ on convergence of the SGD algorithm, we consider a toy example of a 2D optimization problem. Specfically, we consider a loss function $f_\kappa(x, y) = x^2 + \kappa y^2$ where $\kappa > 1$ becomes the condition number of the problem. Qualitatively, the effect of the first term diminishes exponentially fast with κ (Supplementary Fig. 5a). Quantitatively, we run both SGD and Adam optimization for a 1000 iterations starting from the point $(x, y) = (5, 5)$. Supplementary Fig. 5c shows that SGD works extremely well for $\kappa = 1$ which is the best-conditioned loss function, but quickly gets stuck for $\kappa \geq 100$. On the contrary, Adam is invariant to the condition number and converges to

the minima for all values of κ. This is because for a diagonal Hessian (as in this case), the second-order adaptive terms are proportional to the diagonal elements of the Hessian. These condition numbers are vanishingly small compared to those in typical image registration tasks, which can exceed 10^5, making them extremely ill-conditioned.

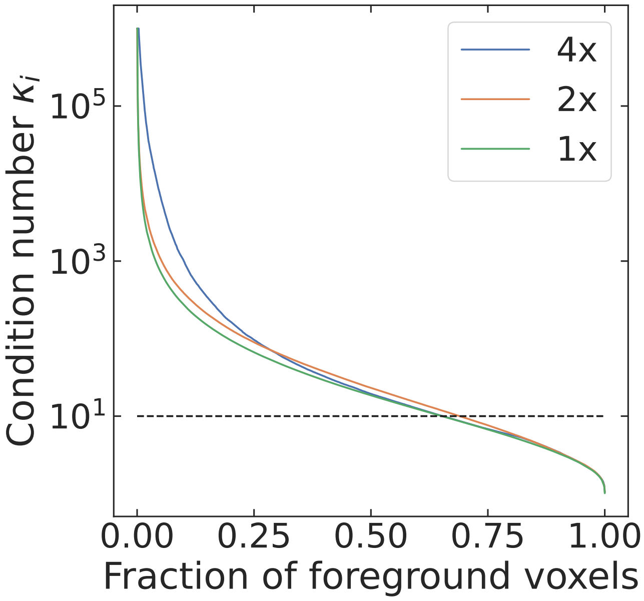

Supplementary Figure 1: Deformable image registration is ill-conditioned. To quantitatively examine ill-conditioning in registration, we compute the distribution of per-pixel condition number for a MRI registration task, at different image downsampling factors (denoted as 1x, 2x, and 4x). A high condition number signifies exacerbated ill conditioning and requires higher-order optimization. A horizontal dashed line denoting $\kappa = 10$ is drawn as a reference for substantial ill conditioning. Across all scales, a substantial fraction of foreground voxels are ill-conditioned ($\kappa > 10$), necessitating adaptive first-order optimization for faster convergence and accurate registration.

We also consider a more realistic, but tractable scenario of the convex loss function $f_{\kappa,\theta}(x, y) = x_\theta^2 + \kappa y_\theta^2$, where

$$\begin{bmatrix} x_\theta \\ y_\theta \end{bmatrix} = \begin{bmatrix} \cos(\theta) & \sin(\theta) \\ -\sin(\theta) & \cos(\theta) \end{bmatrix} \begin{bmatrix} x \\ y \end{bmatrix}$$

We choose $\theta = \pi/3$ for this experiment. This is simply a rotated version of the previous family of loss functions, as shown in Supplementary Fig. 5b. The trajectories obtained from optimization using SGD (Supplementary Fig. 5d) are virtually identical to that in Supplementary Fig. 5c since the new gradients are simply rotated versions of the previous gradients, and the distance from the minima is invariant to the rotation. However, the trajectories from Adam optimization are qualitatively very different, owing to the increasing difference between the true Hessian and its diagonal approximation. Even so, the final point is at a distance of less than 10^{-3} units to the minima for $\kappa = 1000$, showing the effectiveness of adaptive optimization even for ill-conditioned, non-diagonal Hessians. This is a strong motivation to extend adaptive optimization for non-Euclidean diffeomorphic registration, which is very high-dimensional and ill-conditioned.

E Limitations of Stationary Velocity Fields

A common approach in diffeomorphic registration is to use stationary velocity fields, i.e. velocity fields that are constant in time. This velocity field is also an element of the Lie algebra that can be used to generate a diffeomorphism using the exponential map. Since the velocity field itself resides in Euclidean space, adaptive optimization algorithm like Adam can be applied to optimize the velocity field. Many deep learning methods employ this approach since it is hard to produce valid diffeomorphic transforms using a network but it is easy to produce a valid stationary velocity field. CLAIRE[43] mentions the limitations of this approach without further elaboration. We discuss three limitations of the SVF based optimization approach:

Computational Cost Optimizing the velocity field requires computing the exponential map using the scaling-and-squaring approach ((3)) to obtain the diffeomorphism. Typical registration pipelines run scaling-and-squaring 6-8 times to obtain the diffeomorphism, and the backprop requires another 6-8 steps of iterative backward calls to compute the gradient of the velocity field. This is a significantly expensive operation performed *every iteration* of the optimization, leading to a substantial slowdown in runtime. In contrast, direct optimization requires only one warp composition to perform the diffeomorphic update $\varphi_{t+1} = \varphi_t \circ (id + \eta_t v_t)$. The significant difference in runtime is observed for ANTs and DARTEL in Fig. 4b. SVFs also cannot represent diffeomorphisms that are integrals of time-dependent velocity fields, which are more flexible and can represent a wider range of deformations[15,44,43].

Tradeoff between Numerical Accuracy and Computational Cost Exponential maps of SVFs are mathematically diffeomorphic in nature, but it is observed that numerically SVFs may not be diffeomorphic. Other works[15] show that SVF based baselines like Log Demons have significantly more non-diffeomorphic voxels than time-dependent velocity fields like SyN and NODEO. We see similar trends in Fig. 2c, where the SVF based DARTEL has significantly more non-diffeomorphic voxels than the direct optimization in ANTs and FireANTs. This numerical inaccuracy traces back to the discretization error in the scaling and squaring approach, which is essentially Euler integration of the velocity field. In the base case of the scaling-and-squaring approach ((2)), $\varphi^{(1/2^M)} = id + v_0/2^M$ is not guaranteed to be a diffeomorphism unless the Lipschitz constant of v_0, denoted as $LP(v_0)$ is less than 2^M. The inductive recursion step ((3)) only preserves the diffeomorphic property if the base case is a diffeomorphism, otherwise the non-diffeomorphism can propagate throughout the subsequent steps to the final diffeomorphism. Here, we provide a proof that the base case is a diffeomorphism only for v_0 with Lipschitz constant less than 2^M, showing that velocity fields with large deformations or highly variable deformations require finer Euler integration steps (i.e. larger M) to ensure numerical diffeomorphisms.

Theorem 1. For a $C^\infty(\Omega, \mathbb{R}^d)$ velocity field v_0 with compact support on Ω such that $v_0(x) = 0$ on $x \in \partial\Omega$, the transform $\varphi = id + \epsilon v_0$ is a diffeomorphism for $|\epsilon| < 1/LP(v_0)$, where $LP(v_0)$ is the Lipschitz constant of v_0.

Proof. Since v_0 is a $C^\infty(\Omega, \mathbb{R}^d)$ (is continuously differentiable and is compact on Ω) velocity field, the Jacobian of the velocity exists, and is denoted as $J(v_0)$. We invoke the Hadamard's global inverse function theorem[45] (HGIF theorem) to show that φ is a diffeomorphism for $|\epsilon| < 1/LP(v_0)$.

The HGIF theorem requires that φ is smooth (true by our definition), and the Jacobian of the transformation is non-singular for all $x \in \Omega$, and that $\|J\varphi(x)^{-1}\|$ is bounded for all $x \in \Omega$.

Since v_0 is defined only on a compact domain Ω, we use the Whitney extension theorem[46] to extend v_0 to a $C^\infty(\mathbb{R}^d)$ velocity field by simply setting $v_0(x) = 0$ for $x \in \mathbb{R}^d \setminus \Omega$.

For $x \in \mathbb{R}^d \setminus \Omega$, we have $\varphi(x) = x$, and therefore $J\varphi(x) = I$ which is invertible, and $\|J\varphi(x)^{-1}\| = 1$ which is bounded.

For $x \in \Omega$, we have

$$J\varphi(x) = I + \epsilon J v_0(x) \tag{7}$$

$$\Rightarrow \|J\varphi(x) - I\| = |\epsilon| \|Jv_0(x)\| \leq |\epsilon| LP(v_0) < 1 \tag{8}$$

since $|\epsilon| < 1/LP(v_0)$. Since $\|J\varphi(x) - I\| < 1$, $J\varphi(x)$ is non-singular for all $x \in \Omega$ from the Neumann convergent series of matrix $(I - A)$ (i.e. $A^{-1} = \sum_{k=0}^{\infty}(I - A)^k$).

For $A = J\varphi(x)$, we have $\|I - A\| < 1$ from the above inequality. Let $\|I - A\| \leq \delta$ for some $\delta < 1$. Using the Neumann convergent series of matrix $(I - A)$ (i.e. $A^{-1} = \sum_{k=0}^{\infty}(I - A)^k$ for $\|I - A\| < 1$), we have

$$\|A^{-1}\| \leq \sum_{k=0}^{\infty} \|(I - A)^k\| \tag{9}$$

$$\leq \sum_{k=0}^{\infty} \delta^k = \frac{1}{1 - \delta} \tag{10}$$

This shows that $\|J\varphi(x)^{-1}\| \leq \frac{1}{1-\delta}$ for all $x \in \Omega$ and is bounded.

Since φ is $C^\infty(\mathbb{R}^d, \mathbb{R}^d)$, and the Jacobian is non-singular and its inverse is bounded for all $x \in \mathbb{R}^d$, we have that φ is a diffeomorphism for all $x \in \mathbb{R}^d$. □

If $|\epsilon| \geq 1/LP(v_0)$, then $\|J\varphi(x) - I\| < 1$ may not hold and the Jacobian may be singular for some $x \in \Omega$, breaking local invertibility. When scaling-and-squaring is employed, the velocity field might have large magnitudes to capture large deformations during optimization, leading to a large Lipschitz constant. Fixing the number of integration steps M can lead to numerical non-diffeomorphisms if the Lipschitz constant exceeds 2^M. In principle, M should adaptively chosen to the lowest value such that $2^M > LP(v_0)$.

Empirical Verification To demonstrate this empirically, we choose three 1D velocity fields over the interval $\Omega = [-1, 1]$ with increasing Lipschitz constants (illustrated in Supplementary Fig. 2), and plot the amount of non-diffeomorphic voxels as a function of M. We choose 1D velocity fields for simplicity and easy visualization but all results generalize to higher dimensions. The three velocity fields are named and defined as follows:

- Simple gaussian: $v(x) = \exp(-5x^2)$
- Gaussian with sinusoidal: $v(x) = \exp(-5x^2)(1 + \sin(20\pi x))$
- Complex modulation: $v(x) = \exp(-5x^2)(1 + 0.7\sin(\pi\exp(|\pi x|^3)))$

The fraction of non-diffeomorphic voxels as a function of M is shown in Supplementary Fig. 3, along with the Lipschitz constant of the velocity fields. This plot shows that the Simple Gaussian velocity field required only 1 integration step to ensure a diffeomorphism over Ω but the sinusoidal velocity field required 6 steps and the complex modulation velocity field required 10 steps. Note that all three velocity fields are bounded by 1, but their Lipschitz constants are different by orders of magnitude. The result of the exponential map computed with increasing number of

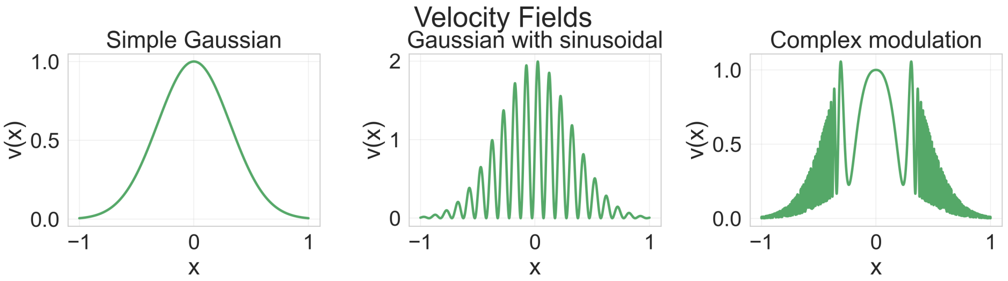

Supplementary Figure 2: Interplay between Lipschitz constant and number of integration steps M for scaling-and-squaring. Three 1D velocity fields with increasing Lipschitz constants to illustrate the dependence of the number of integration steps M on ensuring numerically accurate diffeomorphisms.

integration steps M for each velocity field is also visualized in Supplementary Fig. 4. Velocity fields with larger Lipschitz constants require a larger number of integration steps regardless of the actual magnitude of the velocity field. Since the Lipschitz constant of the velocity field is not known a priori, this creates a tradeoff between numerical accuracy and computational cost.

In contrast, when optimizing diffeomorphisms directly using the update rule $\varphi_{t+1} = \varphi_t \circ (id + \epsilon_t v_t)$, the scaling factor ϵ_t is chosen to be $\eta / LP(v_t)$ where η is the learning rate. $LP(v_t)$ can be bounded by the half of the norm of the velocity field divided by the resolution of the image, since we have

$$LP(v_t) = \sup_{x,y\in\Omega} \frac{\|v_t(x) - v_t(y)\|}{\|x-y\|} \leq \sup_{x'\in\Omega} 2\frac{\|v_t(x')\|}{\|\delta x'\|} \tag{11}$$

avoiding the need to compute the Lipschitz constant directly.

Sensitivity to Perturbations and limits of Expressivity Another potential source of numerical instability arises from the sensitivity of diffeomorphisms to perturbations in their underlying velocity fields. While perturbation and sensitivity analyses are well established for matrix exponentials, often showing that output deviations grow exponentially with the norm of input perturbations[47,48]. Several works have also investigated the singularities of the Euler equation [49,50,51] that leads to blowups in geodesic flows that represent diffeomorphisms. Unlike finite-dimensional Lie groups, the derivative of the exponential can fail to be surjective, possibly producing ill-conditioning and numerical instability near certain vector fields representing conjugate directions[52]. SVFs have a few limitations in regards to expressivity. For example, there are diffeomorphisms arbitrarily close to the identity that are not contained in flows (1-parameter subgroups or SVFs)[53], showing that the exponential map is not surjective to the group of diffeomorphisms even locally. The strong dependence on initial conditions and parameters observed in such systems suggests that analogous sensitivities may contribute to numerical instability and inexpressivity in SVF-based optimization methods. This is empirically observed in Supplementary Fig. 12, where the *exp* representation underperforms for the same cost function and dataset, potentially due to instability and inexpressivity being factors in the small performance degradation since the other parameters of the optimization (loss function, regularization) are kept constant or determined using cross-validation (optimal learning rate, for example).

Direct optimization of diffeomorphisms do not suffer from these limitations, since the perturbations of the diffeomorphism (outputs) are controlled directly by the magnitude of the velocity field in the update rule $\varphi_{t+1} = \varphi_t \circ (id + \epsilon_t v_t)$, and any diffeomorphism close to the

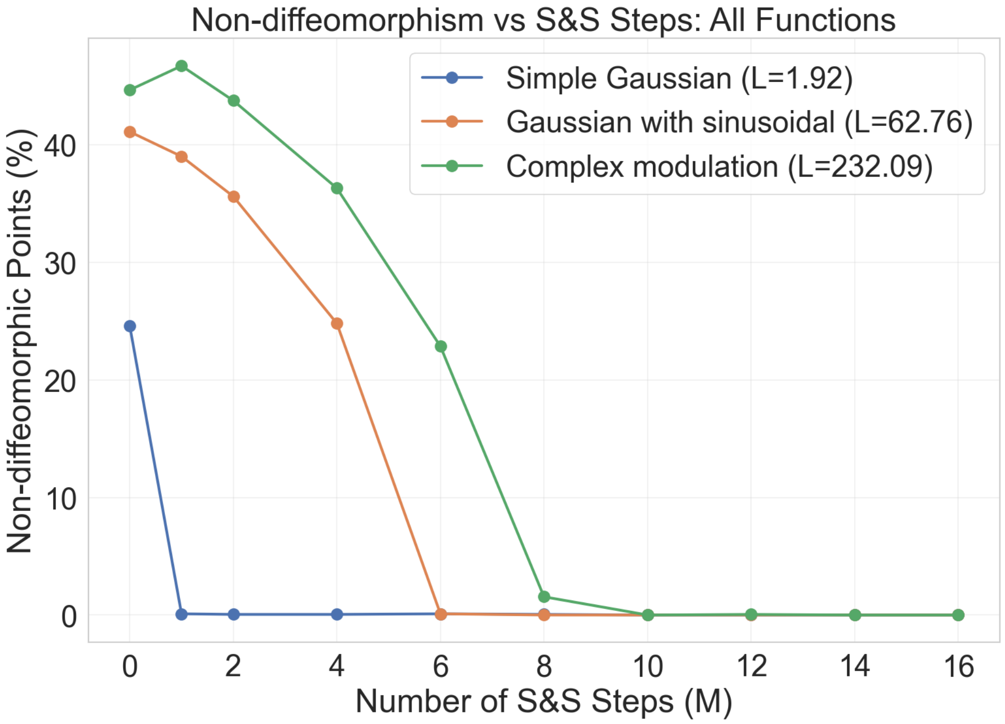

Supplementary Figure 3: Fraction of non-diffeomorphic voxels as a function of M for the three velocity fields. Empirical verification of the dependence between Lipschitz constant and number of integration steps M to ensure numerical diffeomorphisms. This limits the expressivity of SVF based optimization methods.

identity can be obtained trivially by controlling v_t and ϵ_t appropriately.

Supplementary Table 1: Quantitative performance on OASIS validation set. FireANTs performs competitively with state-of-the-art registration methods on the OASIS dataset on both Dice Overlap and Haussdorf distance.

**Validation metrics on OASIS**		
**Method**	**Dice**	**HD95**
Affine (Baseline)	0.572 ± 0.051	3.831 ± 0.718
ANTs [20]	0.786 ± 0.033	2.209 ± 0.534
VoxelMorph [14]	0.753 ± 0.145	-
LogDemons [36]	0.804 ± 0.022	2.068 ± 0.448
SynthMorph [30]	0.785 ± 0.023	2.311 ± 0.452
FireANTs	0.791 ± 0.028	2.793 ± 0.602

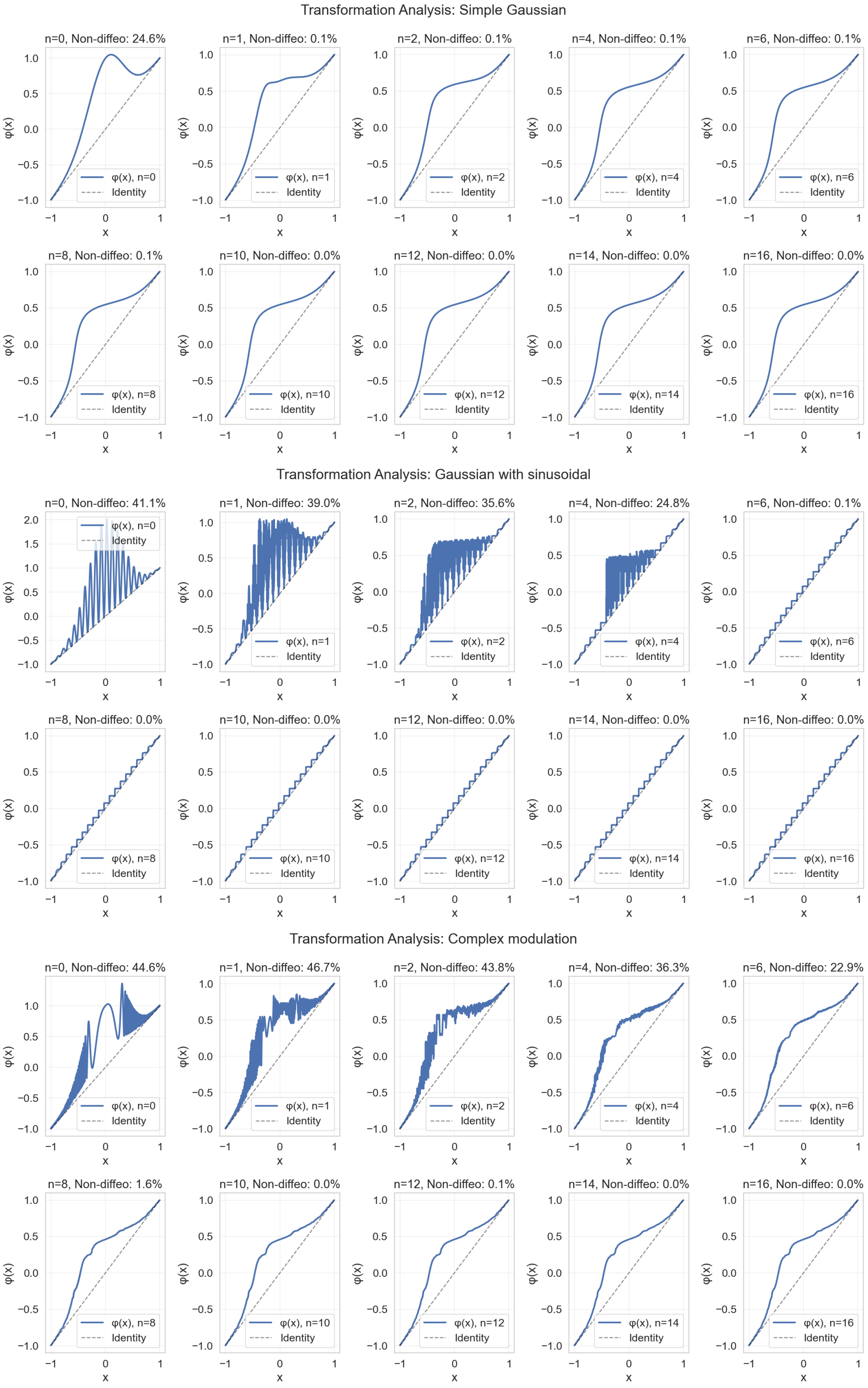

Supplementary Figure 4: Illustrative example of the effect of the number of integration steps M for scaling-and-squaring and the final deformation obtained for the three velocity fields. Larger Lipschitz constants require a larger number of integration steps to ensure numerical diffeomorphisms with the scaling-and-squaring approach.

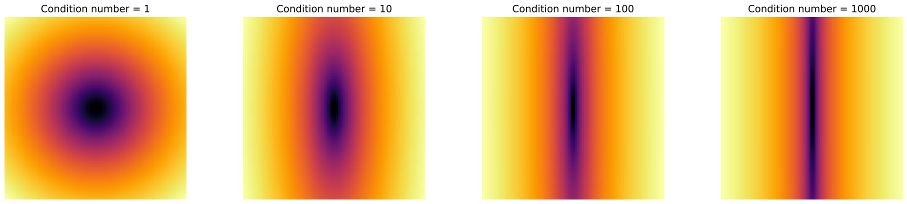

(a) Log-Loss landscape of the toy problem $f_\kappa(x, y) = x^2 + \kappa y^2$ for $\kappa = 1, 10, 100, 1000$. The log-loss becomes increasingly sharp along the y-direction as κ increases.

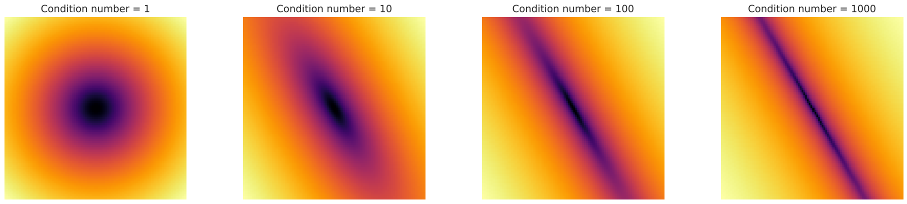

(b) Log-Loss landscape of the toy problem $f_\kappa(x, y) = x_\theta^2 + \kappa y_\theta^2$ for $\kappa = 1, 10, 100, 1000$, where (x_θ, y_θ) is the coordinate (x, y) rotated by an angle θ about the origin.

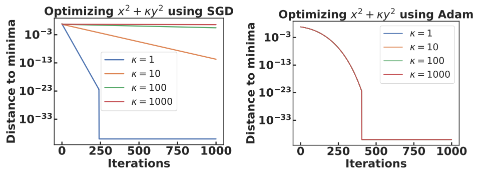

(c) Optimization of Supplementary Fig. 5a using SGD and Adam shows that SGD fails to recover the minima for $\kappa \geq 100$ while Adam is *invariant* to the condition number for diagonal Hessian matrices. This is a strong motivation to use first order adaptive optimization for registration where the condition number can exceed 10^5.

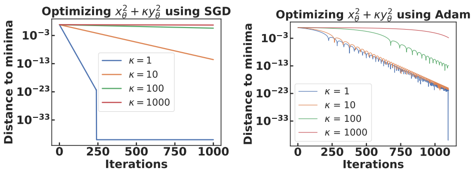

(d) Optimization of Supplementary Fig. 5b using SGD shows identical optimization trajectories as Supplementary Fig. 5c. Adam, however, is not invariant to the condition number because the difference between the true Hessian and its diagonal approximation increases with κ. Even so, the final point is at a distance of less than 10^{-3} units to the minima, showing the mitigating effect of adaptive optimization even for non-diagonal Hessians.

Supplementary Figure 5: Toy problems to illustrate the effect of the condition number on vanilla and adaptive optimization. (a): Log-loss landscape of a toy problem with increasing condition number along the y-direction. Intuitively, gradients become increasingly less informative as the condition number increases. **(b)**: Log-loss landscape of the same toy problem rotated by an angle θ about the origin. **(c)**: Optimization of the toy problem using SGD and Adam shows that SGD fails to recover the minima for $\kappa \geq 100$ while Adam is *invariant* to the condition number for diagonal Hessian matrices. **(d)**: Optimization of the toy problem rotated by an angle θ about the origin using SGD and Adam shows that SGD fails to recover the minima for $\kappa \geq 100$ while Adam is *more robust* to the rotation.

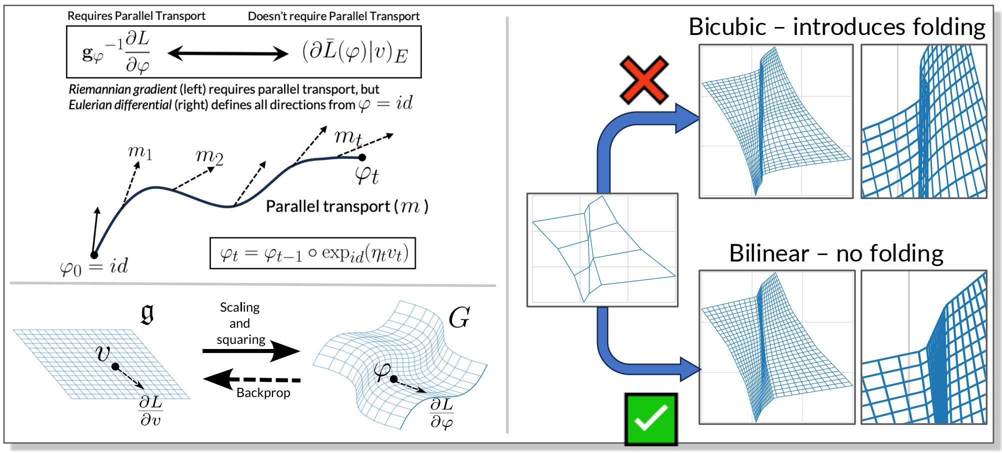

Supplementary Figure 6: Technical framework of FireANTs: **Left** shows the technical contributions of FireANTs. We extend Adaptive Optimization to multi-scale Diffeomorphisms by first writing the Riemannian gradient update, and then avoiding parallel transport of the optimization state by leverging the interchangability of the Riemannian gradient at arbitrary transform φ_t with the Riemannian gradient at $\varphi = \mathbf{Id}$. For the Lie-algebra representation, the Gateaux derivative $\frac{\partial L}{\partial \varphi}$ is projected to $\frac{\partial L}{\partial v}$ using analytical backprop. Since the Lie algebra is a vector space, we use standard adaptive optimizers (see §4.3.2 for more details). **Right** takes a closer look at multi-scale interpolation for diffeomorphisms represented as a warp field. Bicubic interpolation can introduce folding of the warp field at a finer resolution due to overshooting, but bilinear interpolation does not. Therefore, we use this for interpolating the warp field and the optimizer state.

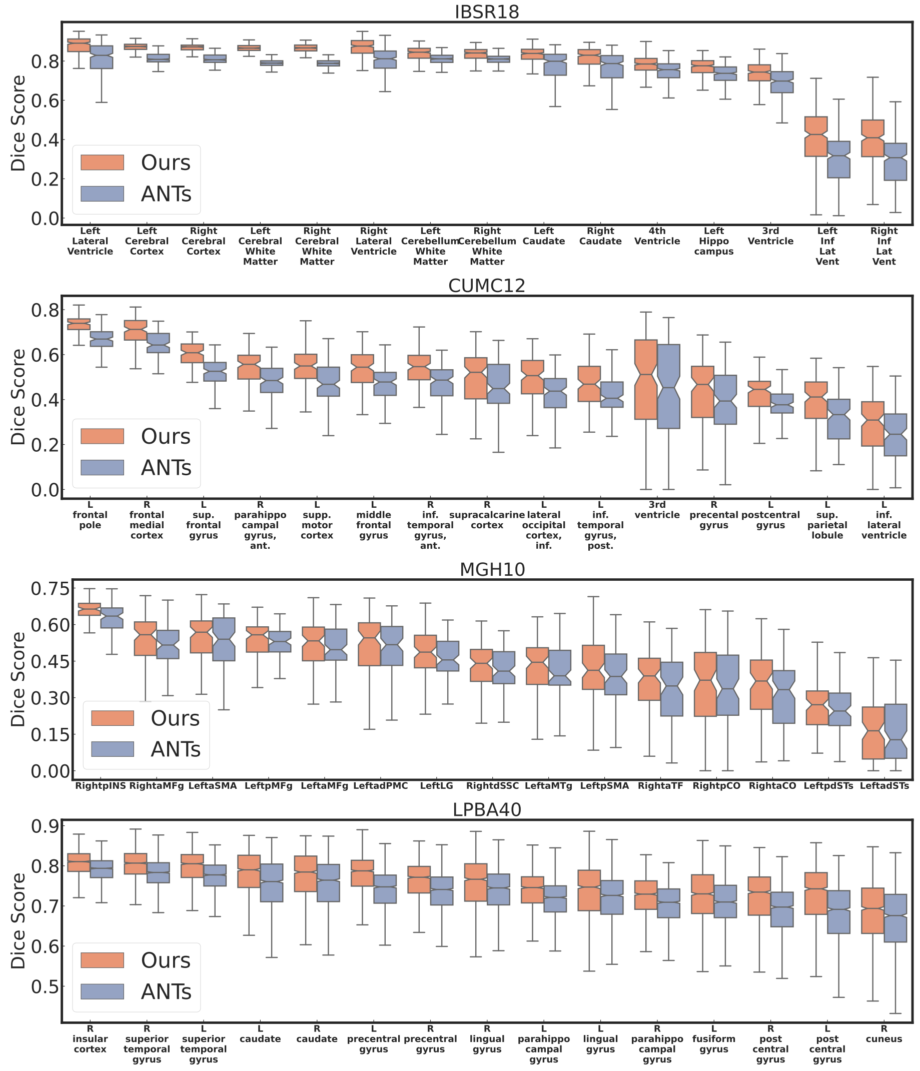

Supplementary Figure 7: Regionwise target overlap on the brain MRI datasets: We further evaluate regionwise overlap scores by sampling 15 regions from each dataset, and comparing their distribution using our method and ANTs. We show results for IBSR18 ($n = 306$), CUMC12 ($n = 132$), MGH10 ($n = 90$), and LPBA40 ($n = 1560$) datasets. Center line in the box plots is the median, and box bounds are the interquartile range (25th–75th percentiles), whiskers extend to the minimum and maximum values within $1.5\times$ IQR of the lower/upper quartiles. Our method has a much higher median score, and better interquartile ranges across regions, demonstrating both accuracy and robustness.

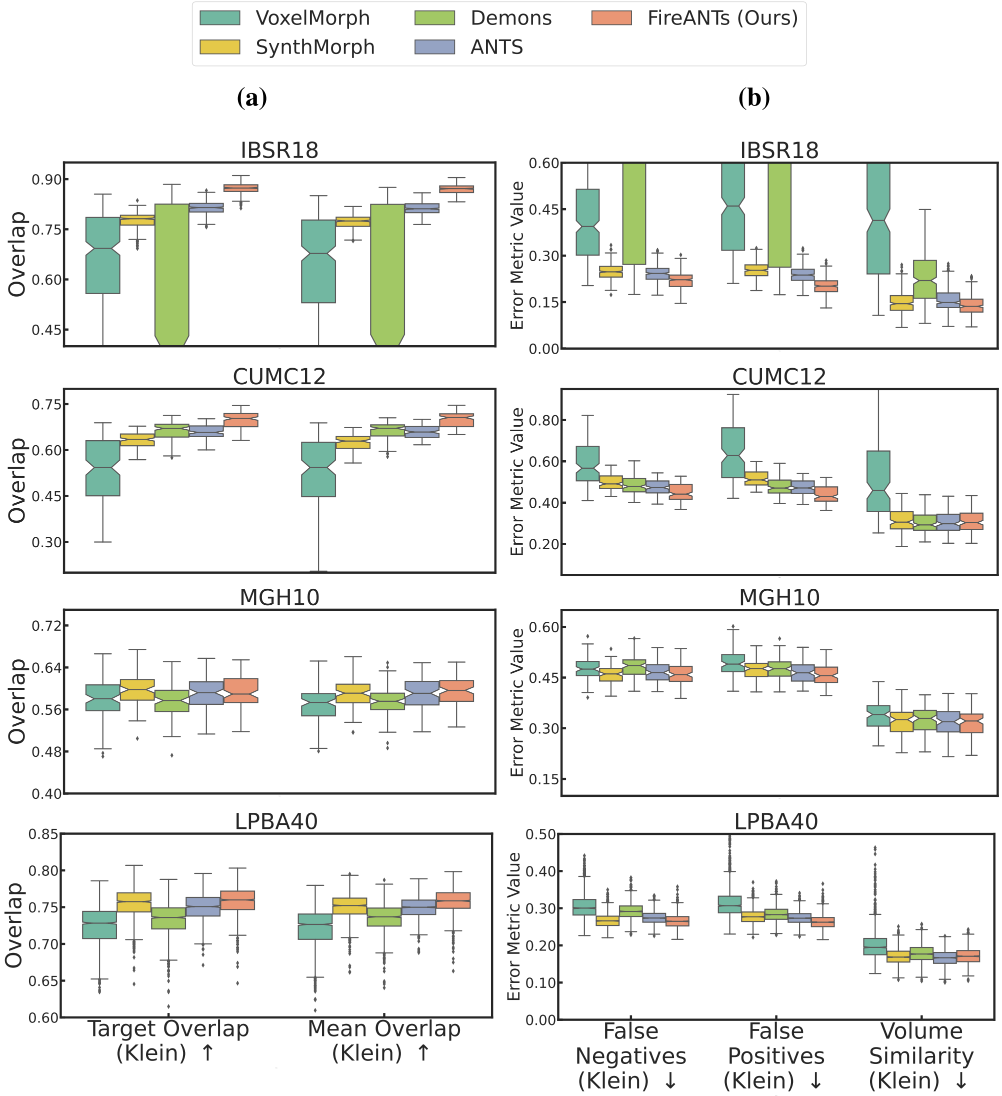

Supplementary Figure 8: Comparison of our method with ANTs on 4 MRI brain datasets: Registration quality is validated by measuring volume overlap of label maps between the fixed and warped label maps. We show results for IBSR18 ($n = 306$), CUMC12 ($n = 132$), MGH10 ($n = 90$), and LPBA40 ($n = 1560$) datasets. Center line in the box plots is the median, and box bounds are the interquartile range (25th–75th percentiles), whiskers extend to the minimum and maximum values within $1.5\times$ IQR of the lower/upper quartiles. Points beyond the whiskers are outliers. **(a)**: For anatomical region r, warped (binary) label map S_r and fixed label map T_r, target and mean overlap are defined as $|S_r \cap T_r|/|T_r|$ and $2|S_r \cap T_r|/(|S_r|+|T_r|)$. We define the aggregate target overlap over all anatomical regions as $\sum_r (|S_r \cap T_r|/|T_r|)$ and Klein *et al.*[34] define it as $(\sum_r |S_r \cap T_r|)/(\sum_r |T_r|)$, likewise for other metrics. The latter aggregation is denoted with the suffix (Klein) in the figure. In all four datasets, the boxplots show a narrower interquartile range and substantially higher median than ANTs (higher is better), underscoring the stability and accuracy of our algorithm. **(b)**: Other measures of anatomical label overlap used in[34] are false positives ($|T_r \backslash S_r|/|T_r|$), false negatives ($|S_r \backslash T_r|/|S_r|$), and volume similarity ($2(|S_r| - |T_r|)/(|S_r| + |T_r|)$) (lower is better). We observe similar trends as in *(a)*, with a narrower interquartile range and substantially lower median values. Results of per region overlap metrics are in the Supplementary Fig. 7.

(a) Trick to avoid parallel transport in Riemannian Adaptive Optimization using Eulerian differentials

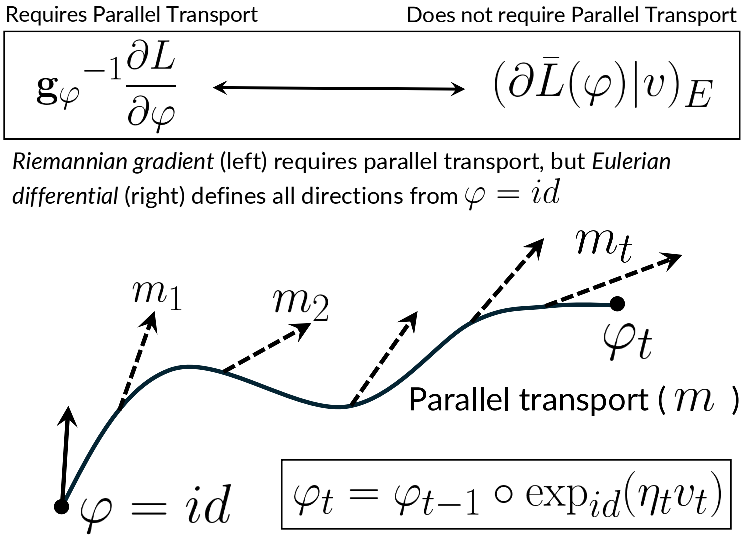

(b) Bicubic interpolation of diffeomorphic map does not preserve diffeomorphism

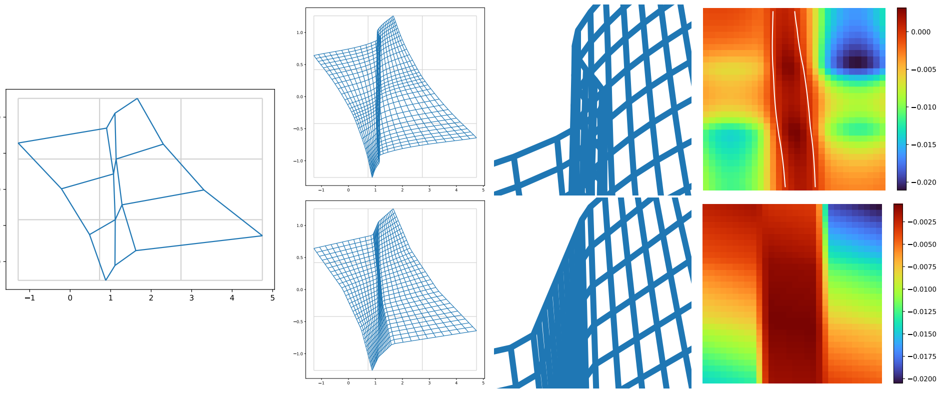

Supplementary Figure 9: Overview of tricks for multi-scale adaptive optimization for diffeomorphisms: (a) We exploit the group structure of diffeomorphisms to define an Eulerian differential that avoids the need for parallel transport in adaptive optimization algorithms. (b) We show the effect of downsampling on the warp and determinant of the Jacobian for a single image pair. The first column shows the initial warp, and the second and third columns show the warp and determinant of the Jacobian for the cubic and bilinear interpolations, respectively.

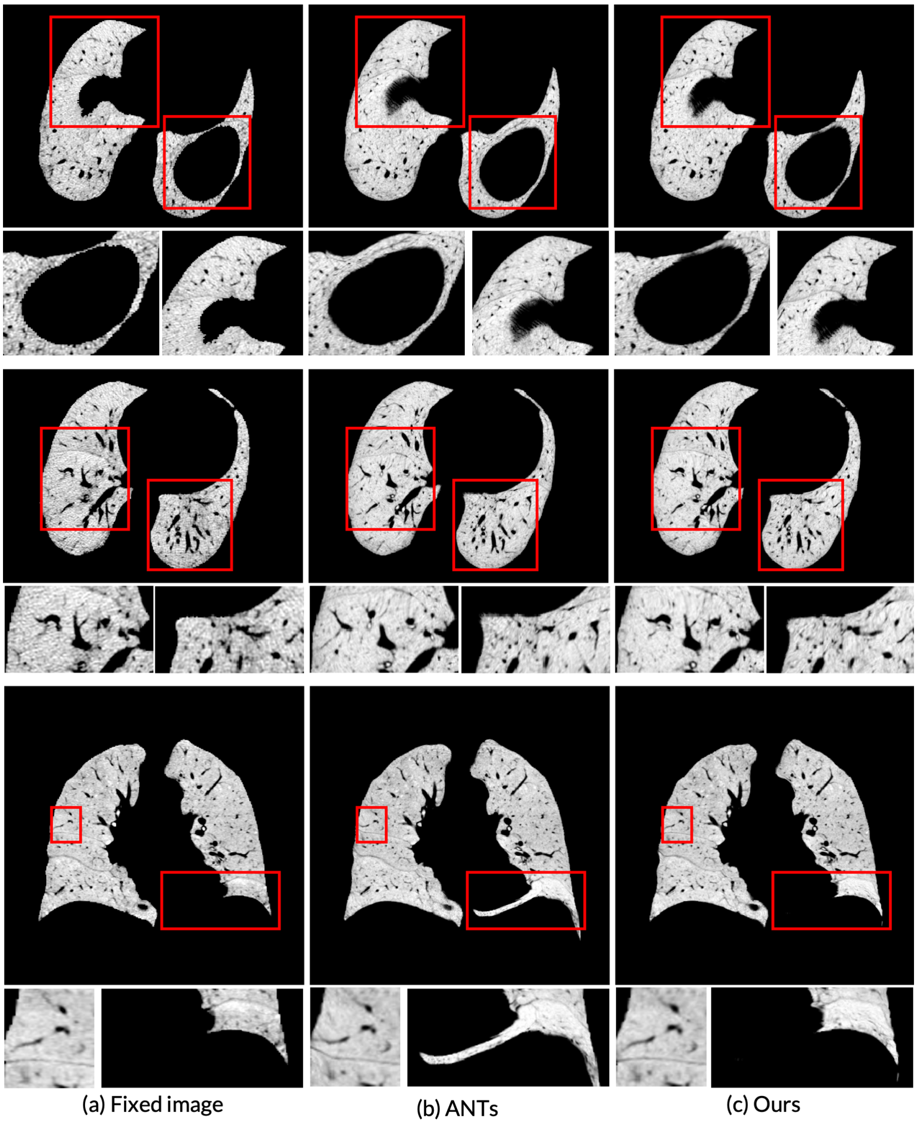

Supplementary Figure 10: Qualitative results on EMPIRE10 challenge: (a) shows the fixed image, (b) shows the registration performed by ANTs, and (c) our method, all with zoomed in regions. ANTs performs a coarse registration with ease, but still leaves out critical alignment of lung boundary and airways by not utilizing adaptive optimization. Our method performs *perfectly* diffeomorphic registration by construction, and does not lead to any registration errors, both in the lung boundaries or internal features.

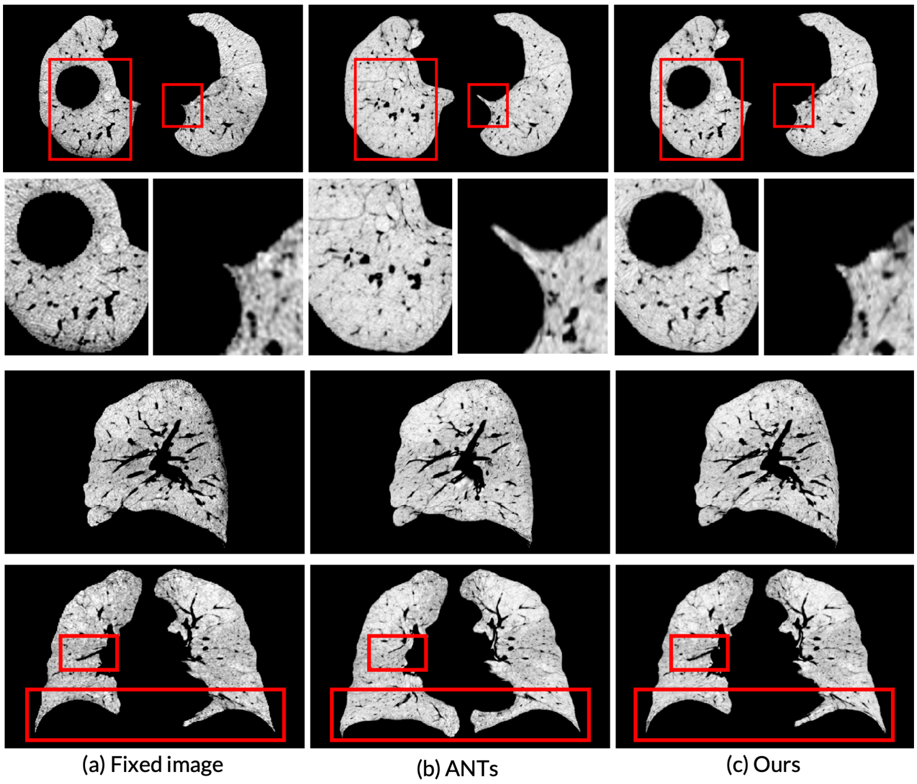

Supplementary Figure 11: More Qualitative results on EMPIRE10 challenge: **(a)** shows the fixed image, **(b)** shows the registration performed by ANTs, and **(c)** our method, all with zoomed in regions. ANTs performs a coarse registration with ease, but still leaves out critical alignment of lung boundary and airways by not utilizing adaptive optimization. Our method performs *perfectly* diffeomorphic registration by construction, and does not lead to any registration errors, both in the lung boundaries or internal features.

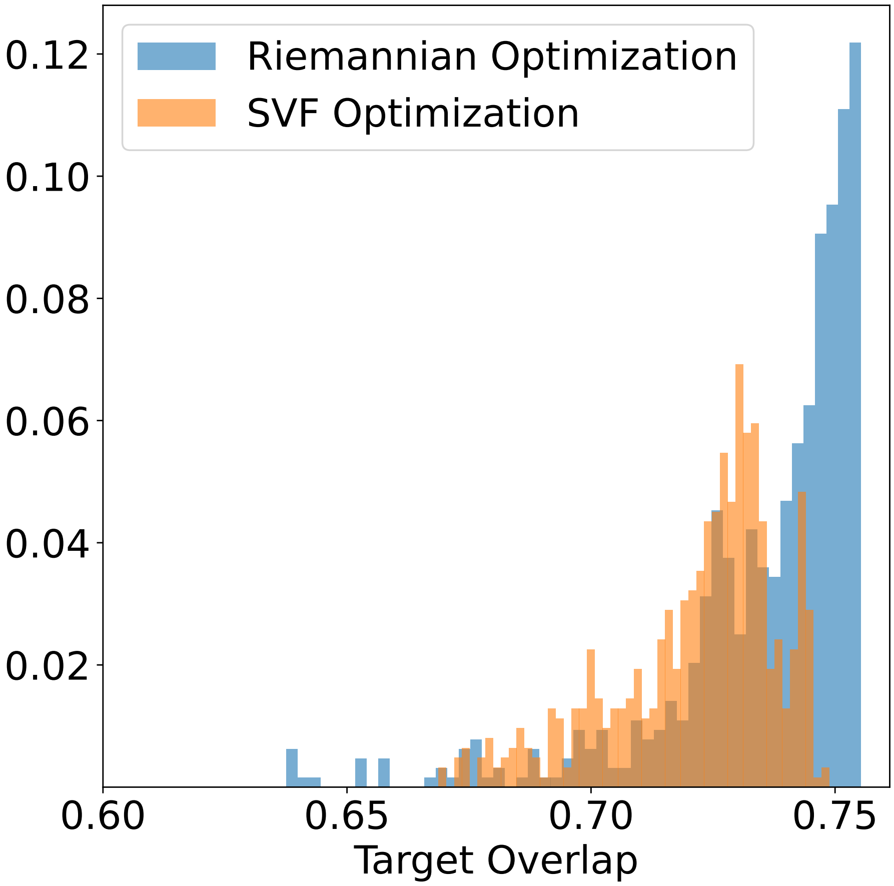

Supplementary Figure 12: Comparison of exponential versus direct optimization on LPBA40 dataset: We run the hyperparameter grid search on the LPBA40 dataset using direct Riemannian gradient updates with Adam optimizer (we denote as as *rgd*), and optimizing the velocity field by computing the exponential map to represent the diffeomorphism (we denote as *exp*) across all the configurations shown in Fig. 5(a). We plot the results with $n = 621$ hyperparameter configurations. The average target overlap for each configuration is then stored, and a histogram of target overlap values of the dataset is constructed. Note that the *rgd* variant has a significantly more number of configurations near the optimal value, and the average performance and the overall distribution of our optimization is better for the *rgd* variant than *exp*. Similar trends can be observed for the EMPIRE10 lung challenge in Fig. 2, where the *exp* representation underperforms for the same cost function, data, etc. Therefore, we recommend direct RGD optimization for diffeomorphisms.

References

[1] U. Grenander and M. I. Miller, "Computational anatomy: An emerging discipline," *Quarterly of applied mathematics*, vol. 56, no. 4, pp. 617–694, 1998.

[2] J. C. Gee, M. Reivich, and R. Bajcsy, "Elastically deforming a three-dimensional atlas to match anatomical brain images," 1993.

[3] J. C. Gee and R. K. Bajcsy, "Elastic matching: Continuum mechanical and probabilistic analysis," *Brain warping*, vol. 2, pp. 183–197, 1998.

[4] G. E. Christensen, R. D. Rabbitt, and M. I. Miller, "Deformable templates using large deformation kinematics," *IEEE transactions on image processing*, vol. 5, no. 10, pp. 1435–1447, 1996.

[5] G. E. Christensen, S. C. Joshi, and M. I. Miller, "Volumetric transformation of brain anatomy," *IEEE transactions on medical imaging*, vol. 16, no. 6, pp. 864–877, 1997.

[6] M. F. Beg, M. I. Miller, A. Trouvé, and L. Younes, "Computing large deformation metric mappings via geodesic flows of diffeomorphisms," *International journal of computer vision*, vol. 61, pp. 139–157, 2005.

[7] J. Ashburner, "A fast diffeomorphic image registration algorithm," *Neuroimage*, vol. 38, no. 1, pp. 95–113, 2007.

[8] M. I. Miller, A. Trouvé, and L. Younes, "Geodesic shooting for computational anatomy," *Journal of mathematical imaging and vision*, vol. 24, no. 2, pp. 209–228, 2006.

[9] P. T. Fletcher, C. Lu, S. M. Pizer, and S. Joshi, "Principal geodesic analysis for the study of nonlinear statistics of shape," *IEEE transactions on medical imaging*, vol. 23, no. 8, pp. 995–1005, 2004.

[10] H. J. Johnson and G. E. Christensen, "Landmark and intensity-based, consistent thin-plate spline image registration," in *Biennial International Conference on Information Processing in Medical Imaging*, pp. 329–343, Springer, 2001.

[11] D. Rueckert, L. I. Sonoda, C. Hayes, D. L. Hill, M. O. Leach, and D. J. Hawkes, "Non-rigid registration using free-form deformations: application to breast mr images," *IEEE transactions on medical imaging*, vol. 18, no. 8, pp. 712–721, 2002.

[12] V. Vishnevskiy, T. Gass, G. Szekely, C. Tanner, and O. Goksel, "Isotropic total variation regularization of displacements in parametric image registration," *IEEE transactions on medical imaging*, vol. 36, no. 2, pp. 385–395, 2016.

[13] A. Reithmeir, J. A. Schnabel, and V. A. Zimmer, "Learning physics-inspired regularization for medical image registration with hypernetworks," in *Medical Imaging 2024: Image Processing*, vol. 12926, pp. 625–635, SPIE, 2024.

[14] G. Balakrishnan, A. Zhao, M. R. Sabuncu, J. Guttag, and A. V. Dalca, "Voxelmorph: a learning framework for deformable medical image registration," *IEEE transactions on medical imaging*, vol. 38, no. 8, pp. 1788–1800, 2019.

[15] Y. Wu, T. Z. Jiahao, J. Wang, P. A. Yushkevich, M. A. Hsieh, and J. C. Gee, "Nodeo: A neural ordinary differential equation based optimization framework for deformable image registration," in *Proceedings of the IEEE/CVF conference on computer vision and pattern recognition*, pp. 20804–20813, 2022.

[16] D. Ulyanov, A. Vedaldi, and V. Lempitsky, "Deep image prior," in *Proceedings of the IEEE conference on computer vision and pattern recognition*, pp. 9446–9454, 2018.

[17] M. Modat, P. Daga, M. J. Cardoso, S. Ourselin, G. R. Ridgway, and J. Ashburner, "Parametric non-rigid registration using a stationary velocity field," in *2012 IEEE Workshop on Mathematical Methods in Biomedical Image Analysis*, pp. 145–150, IEEE, 2012.

[18] B. B. Avants, C. L. Epstein, M. Grossman, and J. C. Gee, "Symmetric diffeomorphic image registration with cross-correlation: evaluating automated labeling of elderly and neurodegenerative brain," *Medical image analysis*, vol. 12, no. 1, pp. 26–41, 2008.

[19] B. Avants and J. C. Gee, "Geodesic estimation for large deformation anatomical shape averaging and interpolation," *Neuroimage*, vol. 23, pp. S139–S150, 2004.

[20] B. B. Avants, N. Tustison, G. Song, *et al.*, "Advanced normalization tools (ants)," *Insight j*, vol. 2, no. 365, pp. 1–35, 2009.

[21] X. Jia, J. Bartlett, T. Zhang, W. Lu, Z. Qiu, and J. Duan, "U-net vs transformer: Is u-net outdated in medical image registration?," *arXiv preprint arXiv:2208.04939*, vol. 1, 2022.

[22] T. C. Mok and A. C. Chung, "Large deformation diffeomorphic image registration with laplacian pyramid networks," in *Medical Image Computing and Computer Assisted Intervention–MICCAI 2020: 23rd International Conference, Lima, Peru, October 4–8, 2020, Proceedings, Part III 23*, pp. 211–221, Springer, 2020.

[23] T. C. Mok and A. C. Chung, "Conditional deformable image registration with convolutional neural network," in *Medical Image Computing and Computer Assisted Intervention–MICCAI 2021: 24th International Conference, Strasbourg, France, September 27–October 1, 2021, Proceedings, Part IV 24*, pp. 35–45, Springer, 2021.

[24] M. Zhang and P. T. Fletcher, "Finite-dimensional lie algebras for fast diffeomorphic image registration," in *International conference on information processing in medical imaging*, pp. 249–260, Springer, 2015.

[25] J. Wang and M. Zhang, "Deepflash: An efficient network for learning-based medical image registration," in *Proceedings of the IEEE/CVF conference on computer vision and pattern recognition*, pp. 4444–4452, 2020.

[26] X. Jia, J. Bartlett, W. Chen, S. Song, T. Zhang, X. Cheng, W. Lu, Z. Qiu, and J. Duan, "Fourier-net: Fast image registration with band-limited deformation," in *Proceedings of the AAAI Conference on Artificial Intelligence*, vol. 37, pp. 1015–1023, 2023.

[27] R. Jena, D. Sethi, P. Chaudhari, and J. Gee, "Deep learning in medical image registration: Magic or mirage?," *Advances in Neural Information Processing Systems*, vol. 37, pp. 108331–108353, 2024.

[28] B. Jian, J. Pan, M. Ghahremani, D. Rueckert, C. Wachinger, and B. Wiestler, "Mamba? catch the hype or rethink what really helps for image registration," in *International Workshop on Biomedical Image Registration*, pp. 86–97, Springer, 2024.

[29] H. Liu, D. Ruan, and K. Sheng, "Unsupervised deformable image registration revisited: Enhancing performance with registration-specific designs," in *Medical Imaging with Deep Learning - Short Papers*, 2025.

[30] M. Hoffmann, B. Billot, D. N. Greve, J. E. Iglesias, B. Fischl, and A. V. Dalca, "Synthmorph: learning contrast-invariant registration without acquired images," *IEEE transactions on medical imaging*, vol. 41, no. 3, pp. 543–558, 2021.

[31] L. Tian, H. Greer, R. Kwitt, F.-X. Vialard, R. San José Estépar, S. Bouix, R. Rushmore, and M. Niethammer, "unigradicon: A foundation model for medical image registration," in *International Conference on Medical Image Computing and Computer-Assisted Intervention*, pp. 749–760, Springer, 2024.

[32] Y. Liu, J. Chen, L. Zuo, A. Carass, and J. L. Prince, "Vector field attention for deformable image registration," *Journal of Medical Imaging*, vol. 11, no. 6, pp. 064001–064001, 2024.

[33] A. C. Evans, D. L. Collins, S. Mills, E. D. Brown, R. L. Kelly, and T. M. Peters, "3d statistical neuroanatomical models from 305 mri volumes," in *1993 IEEE conference record nuclear science symposium and medical imaging conference*, pp. 1813–1817, IEEE, 1993.

[34] A. Klein, J. Andersson, B. A. Ardekani, J. Ashburner, B. Avants, M.-C. Chiang, G. E. Christensen, D. L. Collins, J. Gee, P. Hellier, *et al.*, "Evaluation of 14 nonlinear deformation algorithms applied to human brain mri registration," *Neuroimage*, vol. 46, no. 3, pp. 786–802, 2009.

[35] A. Hering, L. Hansen, T. C. Mok, A. C. Chung, H. Siebert, S. Häger, A. Lange, S. Kuckertz, S. Heldmann, W. Shao, *et al.*, "Learn2reg: comprehensive multi-task medical image registration challenge, dataset and evaluation in the era of deep learning," *IEEE Transactions on Medical Imaging*, vol. 42, no. 3, pp. 697–712, 2022.

[36] T. Vercauteren, X. Pennec, A. Perchant, N. Ayache, *et al.*, "Diffeomorphic demons using itk's finite difference solver hierarchy," *The Insight Journal*, vol. 1, 2007.

[37] K. Murphy, B. Van Ginneken, J. M. Reinhardt, S. Kabus, K. Ding, X. Deng, K. Cao, K. Du, G. E. Christensen, V. Garcia, *et al.*, "Evaluation of registration methods on thoracic ct: the empire10 challenge," *IEEE transactions on medical imaging*, vol. 30, no. 11, pp. 1901–1920, 2011.

[38] L. R. Dice, "Measures of the amount of ecologic association between species," *Ecology*, vol. 26, no. 3, pp. 297–302, 1945.

[39] N. L. S. T. R. Team, "Data from the national lung screening trial (nlst)," 2013.

[40] M. P. Milham, L. Ai, B. Koo, T. Xu, C. Amiez, F. Balezeau, M. G. Baxter, E. L. Blezer, T. Brochier, A. Chen, *et al.*, "An open resource for non-human primate imaging," *Neuron*, vol. 100, no. 1, pp. 61–74, 2018.

[41] L. Mahler, J. Steiglechner, B. Bender, T. Lindig, D. Ramadan, J. Bause, F. Birk, R. Heule, E. Charyasz, M. Erb, V. J. Kumar, G. E. Hagberg, P. Martin, G. Lohmann, and K. Scheffler, ""ultracortex: Submillimeter ultra-high field 9.4t brain mr image collection and manual cortical segmentations"," 2024.

[42] N. Dey, B. Billot, H. E. Wong, C. J. Wang, M. Ren, P. E. Grant, A. V. Dalca, and P. Golland, "Learning general-purpose biomedical volume representations using randomized synthesis," *arXiv preprint arXiv:2411.02372*, 2024.

[43] A. Mang, "Claire: Scalable gpu-accelerated algorithms for diffeomorphic image registration in 3d," in *Explorations in the Mathematics of Data Science: The Inaugural Volume of the Center for Approximation and Mathematical Data Analytics*, pp. 167–215, Springer, 2024.

[44] Y. Wu, M. Dong, R. Jena, C. Qin, and J. C. Gee, "Neural ordinary differential equation based sequential image registration for dynamic characterization," *arXiv preprint arXiv:2404.02106*, 2024.

[45] Hadamard, "Sur les transformations ponctuelles," *Bulletin de la Société Mathématique de France*, vol. 34, pp. 71–84, 1906.

[46] H. Whitney, "Analytic extensions of differentiable functions defined in closed sets," in *Hassler Whitney Collected Papers*, pp. 228–254, Springer, 1992.

[47] C. Van Loan, "The sensitivity of the matrix exponential," *SIAM Journal on Numerical Analysis*, vol. 14, no. 6, pp. 971–981, 1977.

[48] W. Zhu, J. Xue, and W. Gao, "The sensitivity of the exponential of an essentially nonnegative matrix," *Journal of Computational Mathematics*, pp. 250–258, 2008.

[49] T. D. Drivas and T. M. Elgindi, "Singularity formation in the incompressible euler equation in finite and infinite time," *EMS Surveys in Mathematical Sciences*, vol. 10, no. 1, pp. 1–100, 2023.

[50] S. C. Preston, "For ideal fluids, eulerian and lagrangian instabilities are equivalent," *Geometric and Functional Analysis*, vol. 14, no. 5, pp. 1044–1062, 2004.

[51] J. M. Lee, *Geometry and analysis of some Euler-Arnold equations*. City University of New York, 2018.

[52] D. G. Ebin, G. Misio? ek, and S. C. Preston, "Singularities of the exponential map on the volume-preserving diffeomorphism group," *Geometric and Functional Analysis*, vol. 16, no. 4, pp. 850–868, 2006.

[53] J. Milnor, *Remarks on infinite-dimensional Lie groups*. North-Holland., 1984.
