# Supplementary material for: Adaptive Riemannian optimization for multi-scale diffeomorphic matching
Source: Nat Commun. 2026 Jun 9;17:4774. doi: 10.1038/s41467-026-72508-3 (PMC13249883; doi:10.1038/s41467-026-72508-3)
Supplement: Supplementary file 2 — Reporting Summary [file 41467_2026_72508_MOESM2_ESM.pdf]

nature portfolio

Corresponding author(s): James C. Gee

Last updated by author(s): Mar 9, 2026

Reporting Summary

Nature Portfolio wishes to improve the reproducibility of the work that we publish. This form provides structure for consistency and transparency in reporting. For further information on Nature Portfolio policies, see our Editorial Policies and the Editorial Policy Checklist.

Statistics

For all statistical analyses, confirm that the following items are present in the figure legend, table legend, main text, or Methods section.

n/a	Confirmed	
☐	☒	The exact sample size (*n*) for each experimental group/condition, given as a discrete number and unit of measurement
☐	☒	A statement on whether measurements were taken from distinct samples or whether the same sample was measured repeatedly
☒	☐	The statistical test(s) used AND whether they are one- or two-sided<br>*Only common tests should be described solely by name; describe more complex techniques in the Methods section.*
☒	☐	A description of all covariates tested
☒	☐	A description of any assumptions or corrections, such as tests of normality and adjustment for multiple comparisons
☐	☒	A full description of the statistical parameters including central tendency (e.g. means) or other basic estimates (e.g. regression coefficient) AND variation (e.g. standard deviation) or associated estimates of uncertainty (e.g. confidence intervals)
☒	☐	For null hypothesis testing, the test statistic (e.g. *F*, *t*, *r*) with confidence intervals, effect sizes, degrees of freedom and *P* value noted<br>*Give P values as exact values whenever suitable.*
☒	☐	For Bayesian analysis, information on the choice of priors and Markov chain Monte Carlo settings
☒	☐	For hierarchical and complex designs, identification of the appropriate level for tests and full reporting of outcomes
☒	☐	Estimates of effect sizes (e.g. Cohen's *d*, Pearson's *r*), indicating how they were calculated

Our web collection on statistics for biologists contains articles on many of the points above.

Software and code

Policy information about availability of computer code

Data collection	All datasets are public and no software was used on our part for data collection
Data analysis	Code used for data analysis:<br>- FireANTs (our tool) used for image registration: https://github.com/rohitrango/FireANTs (main branch)<br>- ANTs (used as baseline, provides other tools like bias correction for neuroimaging): https://github.com/ANTsX/ANTs (latest stable build)<br>- ITK-SNAP v4.2.2 (used for visualization) : https://www.itksnap.org/pmwiki/pmwiki.php?n=Downloads.SNAP4<br>- SimpleITK (used for data preprocessing and normalization, Demons algorithm): https://github.com/SimpleITK/SimpleITK/ (latest stable build)<br>- Bigstream (used for mouse isocortex dataset) : https://github.com/JaneliaSciComp/bigstream/ (latest commit)<br>- matplotlib (for generating plots) : https://github.com/matplotlib/matplotlib (latest stable build)

For manuscripts utilizing custom algorithms or software that are central to the research but not yet described in published literature, software must be made available to editors and reviewers. We strongly encourage code deposition in a community repository (e.g. GitHub). See the Nature Portfolio guidelines for submitting code & software for further information.

Data

Policy information about availability of data

All manuscripts must include a data availability statement. This statement should provide the following information, where applicable:

- Accession codes, unique identifiers, or web links for publicly available datasets
- A description of any restrictions on data availability
- For clinical datasets or third party data, please ensure that the statement adheres to our policy

All datasets used in the paper are in the public domain:

Brain images used in Klein et.al. neuromapping challenge: https://www.synapse.org/#!Synapse:syn3251018
- requires a synapse account (free) to download, free to view

OASIS and NLST datasets: https://learn2reg.grand-challenge.org/Datasets/
- requires a grand-challenge account (free) to download

EMPIRE10 lung dataset: https://empire10.grand-challenge.org/Download/
- requires a grand-challenge account (free) to download

Expansion microscopy mouse dataset: https://rnr-exm.grand-challenge.org/data/
- requires a grand-challenge account (free) to download

Fluorescence micro-optical sectioning tomography (fMOST) imaging for mouse brain data: https://knowledge.brain-map.org/data/K1YP17A0QIKJOMOAIS4
- available for free
- alternate link used for Get Your Brain Together hackathon (https://insightsoftwareconsortium.github.io/GetYourBrainTogether/) : https://download.brainlib.org/hackathon/2022_GYBS/input/fMOST/subject/

PRIME-DE Macaque dataset: https://fcon_1000.projects.nitrc.org/indi/indiPRIME.html
– free to download

Ultracortex dataset: https://openneuro.org/datasets/ds005216/versions/1.1.0/download
– free to download

Waxholm Rat Brain dataset: https://www.nitrc.org/frs/?group_id=1081#
– free to download

Allen CCFv3 mouse brain dataset: https://atlas.brain-map.org/atlas
– free to download

AZBA Zebrafish dataset: https://azba.wayne.edu/
– free to download

ZBrains Zebrafish dataset: https://zebrafishexplorer.zib.de/
– free to download

Research involving human participants, their data, or biological material

Policy information about studies with human participants or human data. See also policy information about sex, gender (identity/presentation), and sexual orientation and race, ethnicity and racism.

Reporting on sex and gender	N/A. All data from public datasets used in the study are anonymized and no other information about the subject (e.g., race, sex, gender, age, etc.) were used in this study.
Reporting on race, ethnicity, or other socially relevant groupings	N/A. All data from public datasets used in the study are anonymized and no other information about the subject (e.g., race, sex, gender, age, etc.) were used in this study.
Population characteristics	No population characteristic covariates (except imaging) were used in this study.
Recruitment	Recruitment of subjects was done by the authors of the respective public dataset collection studies. Biases of these studies may propagate in the form of imaging artifacts or characteristics which may bias the performance of all baselines and our method equally (since no methods use these covariates directly).
Ethics oversight	N/A

Note that full information on the approval of the study protocol must also be provided in the manuscript.

Field-specific reporting

Please select the one below that is the best fit for your research. If you are not sure, read the appropriate sections before making your selection.

- [x] Life sciences
- [ ] Behavioural & social sciences
- [ ] Ecological, evolutionary & environmental sciences

For a reference copy of the document with all sections, see nature.com/documents/nr-reporting-summary-flat.pdf

Life sciences study design

All studies must disclose on these points even when the disclosure is negative.

Sample size	For all public community-standard datasets and challenges, we use the full dataset and experiment setup as outlined in their respective papers to facilitate fair evaluation of our method. The sample sizes for each dataset were independently deemed sufficient in their respective dataset studies.
Data exclusions	For the template generation experiment, not all subjects were used from the dataset : 56 subjects were used for fMOST and 16 subjects were used for OASIS. The exclusion (inclusion) criteria were based on a random selection of subjects and computational capacity for the baseline.
Replication	All experiments are reproducible since our proposed method is not a stochastic algorithm. Moreover, we fix the random seeds of all methods to ensure any non-deterministic implementations (for example, convolutions used for calculating cross-correlation) are made reproducible.
Randomization	All subjects were selected for all experiments, and random selection was only employed for the template generation experiment.
Blinding	N/A

Reporting for specific materials, systems and methods

We require information from authors about some types of materials, experimental systems and methods used in many studies. Here, indicate whether each material, system or method listed is relevant to your study. If you are not sure if a list item applies to your research, read the appropriate section before selecting a response.

Materials & experimental systems

n/a	Involved in the study
x	Antibodies
x	Eukaryotic cell lines
x	Palaeontology and archaeology
x	Animals and other organisms
x	Clinical data
x	Dual use research of concern
x	Plants

Methods

n/a	Involved in the study
x	ChIP-seq
x	Flow cytometry
x	MRI-based neuroimaging

Plants

Seed stocks	N/A
Novel plant genotypes	N/A
Authentication	N/A
