# Supplementary material for: Adaptive Riemannian optimization for multi-scale diffeomorphic matching
Source: Nat Commun. 2026 Jun 9;17:4774. doi: 10.1038/s41467-026-72508-3 (PMC13249883; doi:10.1038/s41467-026-72508-3)
Supplement: Supplementary file 3 — Transparent Peer Review file [file 41467_2026_72508_MOESM3_ESM.pdf]

nature portfolio

Peer Review File

Adaptive Riemannian Optimization for Multi-Scale Diffeomorphic Matching

Corresponding Author: Professor James Gee

This file contains all reviewer reports in order by version, followed by all author rebuttals in order by version.

Version 0:

Reviewer comments:

Reviewer #1

(Remarks to the Author)
In this paper, the authors propose a new free library FireANTs, dedicated to biomedical image registration. This library extends ANTs, which is one of the most used libraries in medical image re-registration applications since more than 10 years.

Most of the paper deals with applications of FireANTs on various dataset and demonstrate its practical interest. The authors however communicate on the methodological contribution section 4 (adaptive Riemannian Optimization), which is of high interest in the medical image registration community. My main concern with this paper, is that the contribution is not specifically tested, it is extremely poorly discussed. The bibliography is also extremely general and does not specify which strategies have been proposed in more than 40 years of research in medical image registration to take into account the need to regularize deformations at different scales. The methodology is also based on a non-conventional mathematical definition of the image registration problem. Different claims are also false in my opinion.

Although I have no doubt that the implementation of the FireANTs library is of high quality, I would then recommend to reject this contribution. I would also encourage the authors to submit a deeply revised version of the paper to a more methodological journal, after properly assessing and discussing their scientific contribution.

Please find below more specific comments.

(Remarks on code availability)

Major comments:

* Section 2: In a journal like Nature Communications, I would expect to read a discussion of why the authors believe that their methodological choice make their method better that the others. Comparison with other methods may indeed be impacted by pre-processing choices or finer hyperparameter tuning. The only clear message here is that FireANTS is a competitive strategy but not necessarily the best. From a scientific perspective, focusing on the comparison between ANTS and FireANTS would be pertinent to make cristal clear what is the impact of the methodological contributions of Section 4.

* Section 2: The difference of the results between ANTS and FireANTS in figures 1 and 2 does not seem to be significant, which again suggests that the authors should develop more indepth comparisons.

* Section 2.6: Sentence: « The utilization of GPU-based toolkits including FireANTs reduces the runtime from 2-3 days / one week for 1mmin-vivo / 300µm ex-vivo scans on a multi-core workstation, to less than 5 minutes on a GPU » : It seems here that the only reason for the gain of time is the use of GPUs. The speedup due to the use of GPUs and to the contributions of section 4 should be evaluated independently. Speedups of a factor higher than 100 is common when using GPUs in image processing, but the true scientific contribution is what is developed in section 4. This should be evaluated independently.

Section 4.2: The idea in section 4.2. is potentially interesting but deserves to be strongly developed. In particular, the mathematical definition of the spatial regularization term is particularly unclear although it should be central in this paper.

This term if what gives a control on the desired level of regularization in classic registration algorithms since the first optical flow methods. A proper definition of what is minimized is required in an article with solid mathematical foundations.

Sections 4.4 and 4.5: this part of the paper is probably the most interesting one in my opinion. It would deserve to be strongly developed and properly assessed. Here all contributions are tested simultaneously with no discussion of the independent impact of separate contributions

Other comments:

* Abstract: The abstract is well written but is a bit confusing. It is not clear whether the main contribution is a methodology (Hessian esimates) or the design of a fast registration library (the strong speed up).

* Section 1: Sentence « Due to the dense and high-dimensional nature, these methods are often plagued with ill-posedness, curse of dimensionality, difficulty in optimization and scalability to high-resolution data » : These claims and strong and partially false in my opinion. They should be illustrated with solid references. I specifically disagree with the fact that the registration of dense images causes ill-posedness.

* Section 1: Sentences: « Within dense deformations, diffeomorphisms are of special interest … This allows us to accurately model the deformation between images and also ensures that the deformation is physically plausible » : This claim is also false: diffeomorphisms only ensure that the topology of the deformed shapes is preserved. This property is indeed of high interest, but this is different to what the authors wrote.

* Section 1: Again mixing methodological and engineering contributions (GPU implementation) makes the introduction awkward.

* Section 2: DNN methods are used in the tests. Why not introducing them and their benefits in introduction. This could be made from an engineering perspective. How they implicitly spatially regularize the deformations would also be of high interest since this is the topic of this paper.

* Section 3: The discussion section is non-informative. It mainly repeats what was already written in Section 2, plus well known ideas in diffeomorphic image registration. Please discuss different insights illustrating the benefit of the methodology Section 4.

* Section 4.1.1: Sentence « However, the rate of convergence of these algorithms are contingent on the severity of ill-conditioning »: Strong claim with which I partially disagree. Temporal regularization in LDDMM
like algorithms makes the problem well posed and diffeomorphic methods that use stationary velocity fields can hardly address very large deformations with gradient-descent like approaches but are not temporally ill-conditioned.

Reviewer #2

(Remarks to the Author)
This paper presents FireANTs, an adaptive Riemannian optimization algorithm for dense diffeomorphic image registration that extends adaptive Hessian-based gradient updates in the velocity space (a.k.a. tangent space of diffeomorphisms) to the non-Euclidean space of diffeomorphisms. The core contribution of FireANTs lies in leveraging the Lie group structure of diffeomorphisms to develop a Jacobian-free Eulerian differential-based gradient descent scheme that avoids computationally expensive operations for dense image registration. Additionally, the authors introduce multi-scale optimization of the deformation fields given their reduced computational overhead and ease of optimization. Extensive experiments demonstrate faster convergence, with less computational cost with superior accuracy as compared to existing methods such as ANTs and DARTEL.

Dense image registration is a well-known ill-posed and long-standing challenge in the computer vision and medical image analysis communities. The proposed method, which significantly accelerates the widely used ANTs software, holds great promise for advancing biomedical imaging workflows. However, given the rapid progress in deep learning–based registration methods, many of which already achieve superior efficiency and competitive accuracy, the motivation behind this work requires clearer justification. The proposed FireANTs was validated on multiple datasets with encouraging results in terms of both speed and accuracy. While the authors compare their method to a selection of deep learning approaches, the rationale for these choices is not well explained, and several state-of-the-art models that may outperform the proposed method are not included. Furthermore, there are technical aspects of the proposed framework that require justification to validate the claimed advantages. Below are my major questions:

-The introduction does not clearly articulate the novelty or the specific gaps this work addresses beyond computational efficiency. Many classical and deep learning–based registration methods also targeted reductions in time and memory costs,

yet these related efforts are largely underrepresented or insufficiently discussed in the current manuscript. Meanwhile, the literature review is limited, particularly in the introduction. Several related methods that also optimize in the velocity space or use efficient diffeomorphic modeling are not cited or compared. Examples include but not limited to [1-8].

[1] Finite-Dimensional Lie Algebras for Fast Diffeomorphic Image Registration, 2023;
[2] Deepflash: An efficient network for learning-based medical image registration, 2020.
[3] Fourier-net: Fast image registration with band-limited deformation, 2023.
[4] Fourier-net+: Leveraging band-limited representation for efficient 3d medical image registration, 2023.
[5] NeurEPDiff: Neural operators to predict geodesics in deformation spaces, 2023.
[6] Nephi: Neural deformation fields for approximately diffeomorphic medical image registration, 2024.
[7] Nodeo: A neural ordinary differential equation based optimization framework for deformable image registration, 2022.
[8] Diffeomorphic Image Registration With Neural Velocity Field, 2023.

-The rationale behind comparing the selected baseline methods on deep learning–based registration is unclear. Moreover, methods compared in Fig. 2(d–e) show accuracy only without reporting inference times. This weakens the argument that FireANTs is more efficient.

-The method assumes a unitary matrix U(x) in the polar decomposition of the Jacobian J(\phi)=U(x)P(x). This approximation removes all rotational components. While the authors justify that large rotations are rare, this is not universally true, e.g., in lung CT data, where airway and fissure rotations are significant. The impact of this approximation needs to be justified and well supported empirically.

-A valuable addition would be an ablation study comparing Jacobian-free vs. Jacobian-based gradient directions. Meanwhile, the manuscript would benefit from a theoretical analysis of the algorithm’s time complexity to support claims about scalability and efficiency.

-In Fig. 2(b), the claim that FireANTs achieves zero percent singularity errors (compared to SVF or DARTEL) is questionable. Assuming all methods optimize the same objective with different optimization schemes, a detailed explanation of why FireANTs avoids singularities while others do not is needed. Were regularization parameters identical? How were they selected?

- In Fig.4, the hardware configurations used for baseline methods (e.g., ANTs, DARTEL) are not specified. Since FireANTs runs on GPU (A6000), comparing it to CPU-based baselines without clarification loses the fairness.

-The paper would benefit from a better organization, particularly in distinguishing prior work from the authors’ original contributions. Sections 4.3.1, 4.3.2, and much of the content in 4.4 (prior to 4.4.1) largely summarize established methods and should be moved to a dedicated background or related work section.

Minor comments:

-Contrary to Bigstream’s affine registration, the authors claim to preserve the in-frame/in-plane context during their affine registration step for the RnR mouse registration task. Please provide additional details on how the affine matrix is optimized.

-Inconsistent use of symbols - η vs ϵ (update step size).

-Related references, for example, geodesic shooting methods are mentioned without proper citations.

-The manuscript would benefit from thorough proofreading and sentence refinement to improve its overall clarity and writing quality.

(Remarks on code availability)
The code appears to be well-organized and documented. The published repository seems to include all the necessary functionalities; however, this reviewer has not had the opportunity to examine the implementation in detail or perform any testing.

Reviewer #3

(Remarks to the Author)
Summary: Authors propose a new way to optimize for diffeomorphisms for image registration. The technique relies on the Eulerian differential as was discussed in Chapter 10 of Laruent Younes' book on Shapes and Diffeomorphisms. The main technical difference that I can see is that here authors assume that the Jacobian of the diffeomorphism, which is an expensive part to compute, to be positive definite. Based on this assumption, they define a Jacobian-free descent direction. Moreover, they use this in a momentum based optimizer and this leads to tremendous speed ups in the minimization. Authors show results for various datasets.

Strengths:
1. The results this article reports are extremely impressive. Authors indeed solve very challenging registration problems in lightning speeds. Furthermore, based on the metrics they show, the resulting deformation field remains a diffeomorphism.

2. The amount of experimentation in this article is very impressive. Authors thoroughly assessed their methods and compared with state-of-the-art techniques. This is indeed an excellent experimental design and section overall.
3. The technical descriptions of alternative minimization approaches are extremely well done. I believe the method section until 4.4 is really concise yet informative.
4. The proposed method seems sound but I have some questions that I will pose later on.
5. Overall I think this is a great article and with better descriptions of the actual method, it can really shine and would have a significant impact on the field.

Major Weaknesses:
1. It seems to me that the technique is largely motivated from Younes' book - Sections 10.2 and 10.4. I believe this similarity should be further discussed and the developments here can be linked to the method described therein.
2. The implication of the main assumption, which states that the Jacobian of the transformation is positive definite, should be discussed and analyzed.
3. It is unclear to me why the momentum and EMA of the squared gradients can be computed without any special treatment. If Eulerian differential is indeed a Riemannian gradient, then how come the state variables can be used as they are without requiring a parallel transport?
4. The description of the method starting from 4.4 may require a bit more clarifications.
a. I believe the term ϕ_{0t}^v is not defined in the text.
b. The Eulerian differential is denoted as $\partial \bar{U}$ but can it be $\bar{\partial} U$?
c. The definition of the Eulerian differential seems to be the same in between [61] and here, but perhaps how it is used may be different.

(Remarks on code availability)

Reviewer #4

(Remarks to the Author)
This paper proposes a new registration method, named FireANTs. Technically, it mainly claims two contributions. First, it uses the Eulerian differential to get the descent directions, which can be efficiently computed through GPU-based libraries by means of the Gateaux derivatives, leveraging the property that the diffeomorphism is also a Lie group. Second, it validated the proposed method in many datasets and shown that the proposed method is orders of magnitude faster than the SOTA methods. This improved efficiency therefore enables the dense registration across high resolution images (like sub-micron mouse isocortex volumes), which are prohibitively slow when using the traditional method. So, the proposed method can potentially extend the registration application range for a wider imaging field. The paper is well written and organized. The self-inclusion of the paper is good, and the method is clearly elaborated with rigorous formulations. The validation of the experiments shows substantial improvement in computation efficiency.
However, there is one important question that is not explicitly clarified. Is the efficiency improvement contributed to the GPU or the method itself. Because the computation of Gateaux is also expensive. For the comparison baseline methods, like ANTs and DARTEL, are they using the traditional CPU based version or their optimizations are also updated with the GPU version. If these baseline methods are using CPU version, then the comparison may not be fair, and the massive efficiency improvement cannot be only credited to the proposed framework. Also, besides the computation cost, some discussion on the computation complexity of the proposed method, ANTs, and DARTEL can be meaningful to clarify the efficiency originations.
The self-inclusion of the paper is quite good in most sections. However, a) in section 4.1.1, “In this work, we only consider a subgroup of diffeomorphisms”. It would be more comprehensive to point out the ones that “out of” the subgroup of diffeomorphisms. This would enlarge the whole view for the readers to follow the registration problem. b) since one contribution of the paper is the rigorous mathematic framework of the adaptive optimization in this compact supported subgroup of diffeomorphism. However, the essential motivation (from the application view, a large deformation can be decomposed into several small deformations?) of focusing on this subgroup of diffeomorphism is not explicitly discussed.
The paper addresses the condition number’s effect on the optimization, which is related to the cost function of the registration. The logic seems to be, even with diagonal Hessian (either in L2 registration loss or the handcrafted example in A.3), the ill-conditioning of the registration can substantially slow down the optimization. So, adaptive optimization is preferred. However, this is more related to the choice of the optimization strategy, not related to the motivation of proposing the Eulerian differential to get the descent direction. Because the comparison baselines can also use the adaptive optimizations.
The ANTs atlas construction not only aligns the shape but also aligns the intensity appearance. Is this the reason that in Fig. 6, the FireANTs registration looks not as “bright” as ANTs?
It is confusing to understand “Fig. 8 shows that across all resolutions, more than 60% of foreground pixels have a condition number greater than 10”.
The Fig. 9 index caption is missing.
In the comparison experiment, it is not clear whether the validation used the Jacobian-Free Eulerian descent or Jacobian-involved Eulerian descent. Therefore, the immediate question is, are there any big differences between them? What are the Pros and Cons? Some discussions can be helpful to clarify this question.
Two minor typos:
In sec. 2.2, “for deep methods”->”for deep learning methods”.
In sec. 4.3.1, “However, there a number of challenges”-> “However, there are a number of challenges”.
In summarize, this is a good self-included registration paper with well-validated effective registration improvement in practical. However, given the above comments, especially the non-clarified origin of efficiency, therefore, if the paper can

further convince that the efficiency improvement is indeed from the introduction of the framework, not from the GPU involved computation, the technique contribution would be substantial. The reviewer would be in favor of the publication. Otherwise, the reviewer feels that this paper may be more suitable for a specific journal focused on computer vision or image processing.

(Remarks on code availability)
The code of the paper is released with good documentation and the paper can be well reproduced.

Version 1:

Reviewer comments:

Reviewer #1

(Remarks to the Author)
The authors have provided substantial improvements to the article and their answers to my questions are relevant. I would recommend this article for publication in Nature Communication.

(Remarks on code availability)

Reviewer #2

(Remarks to the Author)
Thank you for the authors' thorough responses to my comments. I have no further concerns and am happy to recommend acceptance of the paper.

(Remarks on code availability)

Reviewer #3

(Remarks to the Author)
Authors have answered my concerns and questions.
With the modifications, clarified explanations and the additional results, I believe the article is in a good state and it will surely be an important contribution to the field of biomedical imaging for a diverse set of applications.

(Remarks on code availability)

Reviewer #4

(Remarks to the Author)
The authors did a great job addressing my issues. I do not have any questions.

(Remarks on code availability)

Open Access This Peer Review File is licensed under a Creative Commons Attribution 4.0 International License, which permits use, sharing, adaptation, distribution and reproduction in any medium or format, as long as you give appropriate credit to the original author(s) and the source, provide a link to the Creative Commons license, and indicate if changes were made.
In cases where reviewers are anonymous, credit should be given to 'Anonymous Referee' and the source.
The images or other third party material in this Peer Review File are included in the article's Creative Commons license, unless indicated otherwise in a credit line to the material. If material is not included in the article's Creative Commons license and your intended use is not permitted by statutory regulation or exceeds the permitted use, you will need to obtain permission directly from the copyright holder.
To view a copy of this license, visit https://creativecommons.org/licenses/by/4.0/

Nature Communications manuscript NCOMMS-25-25699-T

Dear Reviewers,

Thank you for the insightful comments and constructive feedback.

We are encouraged by the Reviewers' recognition of the manuscript's contributions, including the technical depth, extensive experimentation setup, and accessibility. To address the concerns with the clarity of ideas and strength of the claims in the paper, we have significantly revised the manuscript. Thematically, our revision clarifies and strengthens two aspects of the work. First, to demonstrate FireANTs's potential to disrupt numerous biomedical and life science workflows compared to other methods, we extend our experiment setup further to include comparisons on fourteen datasets spanning six model systems (human, ovine, non-human primate including Macaca mulatta and Macaque fascicularis, zebrafish, adult mouse, adult rat), three organ systems (brain, lung, abdomen), seven modalities (T1w MRI, T2*w MRI, CT, expansion microscopy, light sheet microscopy, micro-optical sectioning tomography, 9.4T MRI), and over 15,000 image pairs. Second, we improve clarity and strengthen our claims on the improvement in efficiency compared to the community standard ANTs, as well as state of the art deep learning on GPU memory, runtime and scalability to large problem sizes, establishing FireANTs as an efficient and accurate registration framework.

We provide a point-by-point response to the reviewers below which have significantly improved the quality of our manuscript.

Reviewer 1

In this paper, the authors propose a new free library FireANTs, dedicated to biomedical image registration. This library extends ANTs, which is one of the most used libraries in medical image re-registration applications since more than 10 years. Most of the paper deals with applications of FireANTs on various dataset and demonstrate its practical interest.

We appreciate the Reviewer's insightful feedback and their recognition of FireANTs's potential for significant practical impact.

The authors however communicate on the methodological contribution section 4 (adaptive Riemannian Optimization), which is of high interest in the medical image registration community. My main concern with this paper, is that the contribution is not specifically tested,...

Our evaluation setup is centered around comparison with ANTs, including assessment over four community-standard challenges (Klein, OASIS, EMPIRE10, NLST), and a broad range of additional datasets covering zebrafish (AZBA and ZBrains datasets), rodents (CCFv3 mouse brain atlas, Waxholm rat brain atlas, fMOST dataset from the BICCN hackathon), and macaque (PRIME-DE multi-site dataset) model systems, and applications in atlas generation (OASIS and fMOST). This work helps move the field by advancing our knowledge about the ill-conditioning in a typical registration problem, and by systematically studying the potential challenges to obtaining increased efficiency and accuracy in potential alternative designs like applying adaptive optimization on stationary velocity fields (computational cost, dependence on step size to ensure numerical diffeomorphisms, and sensitivity to perturbations), and applying Riemannian Adam on the space of diffeomorphisms (that requires parallel transport for transporting optimization state, which necessitates solving another differential equation). We introduce a new definition of the concept of Eulerian descent using terms that make it convenient to apply existing autodifferentiation packages like PyTorch and avoid computationally expensive steps like parallel transport and exponential map. Our mathematical formulation leads to a novel Adam optimizer that leverages the Lie group structure of diffeomorphisms. Since the primary technical methodological innovation in FireANTs is the powerful quasi-second-order optimizer made possible with our novel mathematical framework, the improvement in accuracy of labelmap, landmark, and intensity alignment is attributed to this new optimizer.

The contribution of efficiency is tested more thoroughly and fairly by comparing ANTs with FireANTs on the CPU and GPU. We increase the number of threads from 8 to 32 for ANTs, which lowers the speedup of FireANTs on GPUs, but we still maintain speedup of up to 2-3 orders of magnitude. On CPUs, FireANTs is up to 7x faster than ANTs with identical number of threads and RAM, specifically demonstrating the runtime improvement on runtime on CPU due to its more efficient implementation.

… it is extremely poorly discussed.

We regret that the Reviewer found the original exposition inadequate or unclear. Section 4 discusses the main technical contribution of enabling adaptive optimizations directly on the space of diffeomorphisms by introducing Preliminaries of the registration problem (Section 4.1), followed by the problem’s ill-conditioned nature (Section 4.2, Appendix D), which has spurred numerous works on quasi-second order optimizers. To our knowledge, there are no in-depth discussions in the existing literature of the quantitative extent of ill-conditioning seen in real-world image registration tasks. We review alternative representations of diffeomorphisms (stationary velocity fields) and highlight their limitations in terms of implementing numerically accurate diffeomorphisms, computational cost of the exponential map, and sensitivity to perturbations that can potentially make optimization difficult. Further, we discuss the computational cost of a naive implementation of Riemannian Adam for diffeomorphisms; specifically, the expensive step of parallel transport required for the optimizer state. Next, we introduce the concept of the Eulerian differential to exploit the Lie group structure for performing adaptive optimization.

While we believe the original discussion was comprehensive, we have included additional discussion in Section 4.4.2 on alternate formulations of the Eulerian differential to justify our choice for defining this differently than in Younes et al., namely, to avoid storing and updating extra terms like $\varphi^{\{-1\}}$

The bibliography is also extremely general and does not specify which strategies have been proposed in more than 40 years of research in medical image registration to take into account the need to regularize deformations at different scales.

The bibliography includes a wide variety of literature spanning seminal contributions in iterative optimization based methods, state-of-the-art deep learning methods, datasets and challenge benchmarks for evaluating registration algorithms, and application areas where image registration is a crucial workhorse operation. We cite some general non-biomedical applications of registration in the introduction to engage with a broad readership. While this work does not focus on regularization, we agree that it represents an important aspect of registration and have expanded the bibliography accordingly for background completeness. Specifically, we cover the most commonly applied explicit regularizers, including bending energy, total variation, and diffusion models, and the implicit regularization induced by the neural network architecture (for deep networks) and by the choice of solution representation (SVF, BSplines).

The methodology is also based on a non-conventional mathematical definition of the image registration problem. Different claims are also false in my opinion.

The non-conventional definition is chosen to be more aligned with recent deep learning formulations. This definition is simpler and more convenient to work with than the traditional definition that uses an auxiliary inverse. We have added Section 4.4.2 that discusses alternate formulations of the Eulerian differential to justify our choice for defining this differently than in Younes et al.

Section 2: In a journal like Nature Communications, I would expect to read a discussion of why the authors believe that their methodological choice make their method better that the others. Comparison with other methods may indeed be impacted by pre-processing choices or finer hyperparameter tuning. The only clear message here is that FireANTS is a competitive strategy but not necessarily the best.

We do not claim that our method is uniformly better than the state-of-the-art - the No Free Lunch theorem [NFL] makes it hard to justify any choice of optimizer and model universally over others. We, however, claim that FireANTs is a very efficient framework that also performs very accurate registration, consuming far fewer resources (time and memory) than other methods - both classical and deep learning methods alike. In that regard, we claim that our method is Pareto-efficient, and believe our experiments provide compelling support for our claims. Specifically, Figures.1-3 and Sections 2.1-2.4 show strong generalization of FireANTs across several benchmarks against state-of-the-art optimization and deep learning methods. Figure 4

and Section 2.5 demonstrate runtime efficiency compared to ANTs on both CPU and GPU, and show the inference runtime and memory advantages compared to SOTA deep learning methods. We believe these results showing strong generalization and scalability for a fundamental image processing task will be of significant interest to the broad readership of a journal like Nature Communications.

[NFL] No Free Lunch Theorem https://ieeexplore.ieee.org/document/1545946

From a scientific perspective, focusing on the comparison between ANTS and FireANTS would be pertinent to make cristal clear what is the impact of the methodological contributions of Section 4.

We agree and FireANTs is now compared with ANTs on 11 datasets spanning more than 15,000 image pairs (OASIS, LPBA40, IBSR18, CUMC12, MGH10, NLST, EMPIRE10, Ultracortex, PRIME-DE, zebrafish, CCFv3 and Waxholm templates), and shows improved performance on all datasets, which were carefully selected to and rigorously test the contributions of Section 4.

* Section 2: The difference of the results between ANTS and FireANTS in figures 1 and 2 does not seem to be significant, which again suggests that the authors should develop more indepth comparisons.

The median overlap scores of FireANTs outperforms the third quartile of all other methods for IBSR18 and CUMC12 – this is, we believe, a very strong result. On the EMPIRE10 dataset, our fissure alignment errors are lower than ANTs by about 70%, and on the NLST dataset, our landmark error is 61.1% lower than that of ANTs. These are, again, very strong relative improvements compared to ANTs.

Nevertheless, we invoke the No-Free Lunch theorem again, which implies that there is no universally superior algorithm, and consequently the gains across all datasets may not be as extreme as the ones shown above. However, we consider a broad spectrum of practical scenarios where FireANTs improves performance compared to ANTs, and does so while being much faster.

Registration speed has become crucially important because modern advances in imaging acquisition technology have dramatically increased both the resolution and volume of acquired data. High-throughput modalities such as light-sheet microscopy, multiplexed histology, and multi-modal MRI now produce terabyte-scale datasets that capture far richer structural and functional information than before. While this opens exciting opportunities for analysis of a semantically richer data, it also amplifies the computational burden of large-scale registration pipelines. In this context, accelerated registration methods like FireANTs is critical - not only to make these analyses tractable at population scales or native resolution levels, but also to enable rapid experimentation, iterative refinement, and real-time feedback in practical workflows.

* Section 2.6: Sentence: « The utilization of GPU-based toolkits including FireANTs reduces the runtime from 2-3 days / one week for 1mmin-vivo / 300μm ex-vivo scans on a multi-core workstation, to less than 5 minutes on a GPU » : It seems here that the only reason for the gain of time is the use of GPUs. The speedup due to the use of GPUs and to the contributions of section 4 should be evaluated independently. Speedups of a factor higher than 100 is common when using GPUs in image processing, but the true scientific contribution is what is developed in section 4. This should be evaluated independently.

We agree with the Reviewer and are grateful for this request. Indeed, we realized that the efficiency claims are obscured by multiple moving parts in the framework. We have significantly revised the manuscript to clarify the source of gains in efficiency. Compared to ANTs, there are two sources of runtime improvements: (1) **GPU usage:** which trivially increases speedup by two to three orders of magnitude, and (2) **efficient implementation and earlier convergence**: which leads to significant speedup on CPU with identical multi-scale iteration parameters and identical number of threads (which we set to 32 in all experiments to ensure a fairer comparison with multi-threaded ANTs). Figure 4(a) shows the runtime of ANTs and FireANTs on five MRI datasets to ablate the effect of varying data distributions on convergence speed. Figure 4(b) shows similar results for the EMPIRE10 dataset, where the runtime for ANTs and DARTEL are obtained from the challenge writeup.

We achieve this speedup in spite of the need in our work to perform additional operations like momentum and squared momentum updates, and adaptive gradient computation. The speed improvement on CPUs can be attributed to (a) faster convergence - FireANTs optimization typically requires far fewer iterations to reach convergence of the similarity loss function - and (b) our re-engineered versions leveraging certain PyTorch CPU functions.

Section 4.2: The idea in section 4.2. is potentially interesting but deserves to be strongly developed. In particular, the mathematical definition of the spatial regularization term is particularly unclear although it should be central in this paper. This term if what gives a control on the desired level of regularization in classic registration algorithms since the first optical flow methods. A proper definition of what is minimized is required in an article with solid mathematical foundations.

Section 4.2 addresses the ill-conditioning of the registration problem - and does not discuss spatial regularization specifically. In general, the paper actually does not address regularization at all; instead it (re-)derives the mathematical formulation required to perform adaptive optimization on the space of diffeomorphisms directly.

Spatial regularization is typically used to handle the ill-posedness of a problem. An optimization problem with a flat loss landscape is ill-posed (i.e. any parameter in the “flat region” is a minimizer) - this is where spatial regularization adds curvature to the loss landscape and biases the solution to have certain behavior. In contrast, we consider the ill-conditioning of the problem

- which refers to a non-uniform eigenspectra of the Hessian of the loss landscape. A simple problem like $f(x, y) = x^2 + 1000000(y - 1)^2$ is not ill-posed (there exists a unique minimizer $(x = 0, y = 1)$) but is ill-conditioned (with a condition number of 1000000). Adding spatial regularization to this problem will not meaningfully improve the convergence of an optimizer like SGD. In contrast, another problem like $f(x, y) = Relu(|x| - 1) + Relu(|y| - 1)$ is well conditioned but ill-posed (any value of (x, y) inside the unit square will minimize the function). Adding a spatial regularization like $R(x, y) = \lambda(x^2 + y^2)$ will bias the optimizer to converge close to the origin.

Sections 4.4 and 4.5: this part of the paper is probably the most interesting one in my opinion. It would deserve to be strongly developed and properly assessed. Here all contributions are tested simultaneously with no discussion of the independent impact of separate contributions

We agree with the reviewer that Sections 4.4 and 4.5 represent the most interesting and consequential parts of the paper, as they bring together all the requisite mathematical components to enable efficient, numerically accurate, and mathematically correct computation of adaptive diffeomorphic optimization.

Section 4.4 formulates the mathematical framework for performing Adam-like updates on the space of dense diffeomorphisms. We welcome reviewer clarification on which specific aspects of this development would benefit from further elaboration. We have added Section 4.4.2 discussing alternate formulations of the Eulerian differential and justify the choice of our particular definition.

* Abstract: The abstract is well written but is a bit confusing. It is not clear whether the main contribution is a methodology (Hessian esimates) or the design of a fast registration library (the strong speed up).

The main contributions of the paper are indeed twofold : we propose both a state-of-the-art optimization for diffeomorphic registration (methodology) **and** an efficient implementation that performs competitively to deep learning methods both on the CPU and GPU, and scales to much larger problems that is not permissible by DL methods and ANTs alike due to high resource requirements. We have updated the Abstract to clarify the contributions.

* Section 1: Sentence « Due to the dense and high-dimensional nature, these methods are often plagued with illposedness, curse of dimensionality, difficulty in optimization and scalability to high-resolution data » : These claims and strong and partially false in my opinion. They should be illustrated with solid references. I specifically disagree with the fact that the registration of dense images causes ill-posedness.

We reiterate the distinction between ill-posedness (a given registration problem having multiple solutions) which is a very well-known problem, and ill-conditioning (the eigenvalue spectrum of the loss landscape) which is a relatively lesser discussed problem due to the high-dimensional

nature of the problem. Ill-conditioning is colloquially mentioned in the literature but we are unable to find any literature that explicitly examines and analyzes the eigenvalue spectrum of a typical registration problem to establish ill-conditioning, and, therefore, have performed this analysis in Appendix D. The condition number is a well established indicator of ill-conditioning of an optimization problem, which is used here in the paper to illustrate the ill-conditioning of a typical image registration task.

Regularization, on the other hand, is applied in image registration precisely to restrict the solution space of the problem (i.e., to mitigate ill-posedness). We have added references [12], [19], [20] that specifically address the ill-posedness of image registration. The revised manuscript reads:

> Due to the dense and high-dimensional nature, these methods are often plagued with ill-posedness [12,19,20], difficulty in optimization, inefficient implementations, and lack of scalability to high-resolution data.

The curse of dimensionality was mentioned because an image of size N has 3N optimizable parameters – the dimensionality of the solution space grows cubically as the size of the problem (R^N vs R^{3N}). We have removed this phrase.

* Section 1: Sentences: « Within dense deformations, diffeomorphisms are of special interest … This allows us to accurately model the deformation between images and also ensures that the deformation is physically plausible » : This claim is also false: diffeomorphisms only ensure that the topology of the deformed shapes is preserved. This property is indeed of high interest, but this is different to what the authors wrote.

We have reworded the phrase. The revised manuscript now reads:

> This allows us to accurately model the correspondence between images while ensuring that the topological structure of the image domain is preserved under the correspondence transformation; i.e., no holes in or folding of the domain are introduced.

Section 1: Again mixing methodological and engineering contributions (GPU implementation) makes the introduction awkward.

We have rewritten the introduction to more clearly delineate the methodological and engineering contributions.

* Section 2: DNN methods are used in the tests. Why not introducing them and their benefits in introduction. This could be made from an engineering perspective.

We have rewritten the discussion section on deep learning methods and their benefits (as well as their limitations with respect to efficiency and scalability).

How they implicitly spatially regularize the deformations would also be of high interest since this is the topic of this paper.

The focus of our work is to enable an adaptive optimization algorithm on the space of dense diffeomorphisms and implement it in efficient software. Our effort does not propose new approaches to regularization, and, therefore, the implicit regularization of deep methods – while interesting in its own right - is not relevant to the discussion and content of this manuscript.

Section 3: The discussion section is non-informative. It mainly repeats what was already written in Section 2, plus well known ideas in diffeomorphic image registration. Please discuss different insights illustrating the benefit of the methodology Section 4.

Following the Reviewer's suggestion, we have substantially rewritten the Discussion section to limit repetition of the content from Section 2 and instead focus on an expanded discussion of the different insights obtained from our experiment setup, the mathematical limitations of alternate representations that justify our proposed mathematical framework, and the implications for large scale and large resolution imaging studies that can now be significantly accelerated with FireANTs.

* Section 4.1.1: Sentence « However, the rate of convergence of these algorithms are contingent on the severity of illconditioning »: Strong claim with which I partially disagree.

Superlinear or quadratic convergence of second-order methods is a well known result in optimization theory [numopt1, numopt2]. Most convergence bounds depend explicitly on the condition number of the optimization problem. Our paper references relevant methods that showcase the improved convergence rate of quasi-second order methods [28, 83, 84, 85]. We do not claim any new convergence bounds or results, and instead rely on established findings to motivate the benefits of an adaptive optimization method for diffeomorphisms.

[numopt1] https://www.ime.unicamp.br/~pulino/MT404/TextosOnline/NocedalJ.pdf
[numopt2] https://link.springer.com/book/10.1007/978-3-319-91578-4

Sections 4.4 and 4.5: this part of the paper is probably the most interesting one in my opinion. It would deserve to be strongly developed and properly assessed. Here all contributions are tested simultaneously with no discussion of the independent impact of separate contributions

As noted earlier in this response, Section 4 discusses in detail the preliminaries and methodological development of a computationally efficient Adam optimizer for diffeomorphisms. This development is extenstively evaluated through experimental comparisons with ANTs that demonstrate consistent improvements in registration quality. The operational contributions are tested on both CPUs and GPUs with respect to ANTs, showing consistent significant reductions in runtime; and the efficiency of FireANTs is demonstrated by comparing resource requirements with state-of-the-art deep learning methods. Deep Learning methods also require significant

resources for training - accounting for this additional cost would strengthen the efficiency advantages of FireANTs even further.

Reviewer 2

Remarks to the Author

This paper presents FireANTs, an adaptive Riemannian optimization algorithm for dense diffeomorphic image registration that extends adaptive Hessian-based gradient updates in the velocity space (a.k.a. tangent space of diffeomorphisms) to the non-Euclidean space of diffeomorphisms. The core contribution of FireANTs lies in leveraging the Lie group structure of diffeomorphisms to develop a Jacobian-free Eulerian differential-based gradient descent scheme that avoids computationally expensive operations for dense image registration. Additionally, the authors introduce multi-scale optimization of the deformation fields given their reduced computational overhead and ease of optimization. Extensive experiments demonstrate faster convergence, with less computational cost with superior accuracy as compared to existing methods such as ANTs and DARTEL.

We appreciate the Reviewer's concise summary of the contributions of our work. We are particularly encouraged by the Reviewer's observations that our "extensive experiments demonstrate faster convergence, with less computational cost with superior accuracy as compared to existing methods such as ANTs and DARTEL" and that our method "significantly accelerates the widely used ANTs software, holds great promise for advancing biomedical imaging workflows".

Dense image registration is a well-known ill-posed and long-standing challenge in the computer vision and medical image analysis communities. The proposed method, which significantly accelerates the widely used ANTs software, holds great promise for advancing biomedical imaging workflows. However, given the rapid progress in deep learning–based registration methods, many of which already achieve superior efficiency and competitive accuracy, the motivation behind this work requires clearer justification.

Regarding further justification of this work with respect to deep learning methods, we add experiments in the revised manuscript which show that deep learning methods do not necessarily achieve superior efficiency, but are instead resource-intensive, and many SOTA deep learning algorithms are in practice slower than FireANTs at inference. Section 2.5 and Figure 4 now compare the efficiency of FireANTs with ANTs and SOTA deep learning methods more holistically. Specifically, Figure 4(c) shows that for a typical OASIS brain scan, FireANTs performs multi-scale optimization in under a second and consumes around 10x less memory than state-of-the-art deep learning methods. FireANTs scales efficiently with image size, while other methods like SynthMorph and VFA run out of memory on an NVIDIA A6000 GPU (48GB) for an image volume only 6 times larger than an OASIS brain image. For context, a typical ex-vivo hemisphere would be anywhere from 8-27 times larger than an in-vivo brain image.

We add the following discussion in the results section of the paper comparing the efficiency of FireANTs with deep learning methods:

> Runtime and memory requirements compared to deep learning methods FireANTs is highly efficient compared to deep learning methods, both in terms of runtime and memory usage. We highlight the efficiency using three experiments: • Accuracy-Runtime tradeoff: We compare the performance, runtime, and memory usage of FireANTs with SOTA deep learning methods averaged over three neuroimaging datasets (LPBA40, Ultracortex, PRIME-DE). • Runtime and Memory requirements with increasing problem sizes: We take an OASIS MRI image pair and progressively upsample it by factors of 2×, 4×, 6×, 8× larger and measure the runtime and memory usage of each method. • Amortized runtime with increasing batch sizes: Amortized runtime over larger batches can improve GPU utilization and hide kernel launch and activation cache loading overheads. We perform batched inference using an OASIS MRI volume for increasing batch sizes, and plot the amortized runtime (i.e. runtime divided by batch size) as a function of the batch size, and keep increasing the batch size until we run out of memory.

Moreover, FireANTs is capable of registering at native resolution the RnR-ExM (expansion microscopy) mouse isocortex dataset, an image that is around 50x larger than MRI, on a single GPU. Existing algorithms can only downsample a 64x downsampled version, and consequently perform suboptimally. This shows the efficiency of FireANTs for standard clinical problems translates seamlessly to much larger problems prominent in biological and life sciences. FireANTs also supports batched inference, further amortizing the runtime to less than 0.25 seconds per pair on a single GPU (Figure4(c), right). We also compare the efficiency of FireANTs with Hypermorph that performs amortized hyperparameter learning in Figure 5(b). Training and inference in Hypermorph takes about 4.37x more GPU hours to generate the same grid search experiment on the LPBA40 dataset.

Our analysis in Figure 4 does not take into account the training time of most deep learning methods, which easily adds hundreds of GPU hours on top of resource requirements for inference, widening the gap in efficiency between FireANTs and other state-of-the-art deep learning methods.

While the authors compare their method to a selection of deep learning approaches, the rationale for these choices is not well explained, and several state-of-the-art models that may outperform the proposed method are not included. Furthermore, there are technical aspects of the proposed framework that require justification to validate the claimed advantages.

We set out to evaluate the generalization to the long tail of modalities in order to establish the broad scope of potential usage of FireANTs (see Figure 1 and Section 2.1). We compare FireANTs with state-of-the-art methods like SynthMorph, Vector Field Attention, and unigradICON that are foundational or domain-agnostic registration models which claim to generalize across modalities. ANTs is added as an ablation to compare the effect of our optimizer on performance. VoxelMorph and SynthMorph are also added in the Klein et. al. and OASIS challenges due to their established performance on these datasets. Evaluation on the EMPIRE10 dataset (Figure 2(e)) is performed by the challenge organizers, and we do not

choose the set of baselines. Although the EMPIRE10 challenge features deep learning methods like VIRNet, the challenge is dominated by classical methods since the CT scans in the challenge are very high resolution and most deep learning methods run out of memory at this resolution. Moreover, all images are of different voxel sizes presenting significant challenges to deep learning methods that require the fixed and moving images to be of the same size. Evaluation on the NLST dataset (Figure 2(f)) compares against deep networks including Im2Grid, VFA, RWC-Net, unigradICON, and SynthMorph that are either domain agnostic or trained on lungs specifically.
Our choice of baselines is therefore determined by (a) recency in claims of SOTA on the tested modality, (b) domain-agnostic models, and (c) independently evaluated performance on the EMPIRE10 and RnR ExM challenges. The performance of FireANTs exceeds these deep learning-based baselines across all the aforementioned challenge datasets, which we believe represents a substantial and comprehensive evaluation undertaking.

The introduction does not clearly articulate the novelty or the specific gaps this work addresses beyond computational efficiency. Many classical and deep learning–based registration methods also targeted reductions in time and memory costs, yet these related efforts are largely underrepresented or insufficiently discussed in the current manuscript.

As noted in the Reviewer’s introductory summary of our work, its innovation lies in a novel mathematical framework that allows quasi-second-order adaptive optimization on the space of diffeomorphisms. This novel formulation allows us to avoid both the expensive and sensitive scaling-and-squaring based optimization with a stationary velocity field and the computationally costly parallel transport associated with Riemannian gradient descent. The proposed method improves in performance substantially compared to ANTs and state-of-the-art deep learning methods.

Deep learning methods do not claim efficiency advantages per se, but rather trade a slow training process for a faster runtime. Most of these methods are compared against CPU-based ANTs, which we believe is unfair. We provide a more apples-to-apples inference time comparison in Figure 4.

We do not consider training times and GPU-hours of deep learning in this manuscript, which we posit is not a one-time cost, as even foundational models like Synthmorph and unigradICON cannot sufficiently cover the long-tail of bio/medical imaging modalities and applications. A training-free, model-free method like FireANTs is therefore expected to be of great practical value to the community by i) greatly improving inference time both on CPU and GPU, ii) using fewer resources (or equivalently processing more data for a given resource budget) and iii) working out-of-the-box on novel modalities and very high resolution (large scale) data. We have compared against deep learning methods that have targeted reductions in time and memory costs, and our analyses in Figures 4(c) and 5(b) show that FireANTs is highly efficient both in runtime and memory. Our manuscript cites CLAIRE and ConvexAdam as related iterative optimization methods that are implemented on GPU. However, CLAIRE does not support any loss function except MSE and optimizes an SVF. We discuss the limitations of adaptive

optimization on SVFs in Appendix E. ConvexAdam is not multi-scale, and runs out of memory on native resolution for images of the size of the OASIS dataset , suffering from scalability challenges. In contrast, FireANTs is primarily aimed at improving robustness and accuracy using multi-scale optimization and support for multimodal registration, scalability, and seamless user experience and extensibility.

Meanwhile, the literature review is limited, particularly in the introduction. Several related methods that also optimize in the velocity space or use efficient diffeomorphic modeling are not cited or compared. Examples include but not limited to [1-8].

Because of the very tight word limit, we struggled with this issue in our original submission. For this revision, we have added a related works section covering both optimization and deep learning methods in a new Appendix (A), with the recommended references included in the discussion.

We agree the papers listed in [1-8] are important. These all rely on some form of approximation (for example, bandlimited velocity fields) to potentially tradeoff accuracy for efficiency. In contrast, we aim to achieve better performance by enabling a more effective adaptive optimizer (which adds more compute instead of reducing it) and achieve better efficiency through a novel implementation that is performant both on CPUs and GPUs. These are fundamentally different ways to achieve efficiency, and we believe the papers are slightly orthogonal to the contribution of this paper. Moreover, the applicability of these methods is limited to the same distribution as the training data (unlike domain-agnostic or foundational methods that we primarily compare against), and the generalization to out-of-distribution is not specifically tested in these works.

Regarding optimizing in the velocity space, our method discusses the Lie algebra representation and the possibility of applying an adaptive optimization on this representation directly (Section 4.3.1). However, there are a few limitations of the velocity field representation that motivate our method to apply adaptive optimization on the diffeomorphism directly. Although a rigorous study of the limitations of velocity field representations is beyond the scope of this paper, we discuss these issues in substantial detail in Appendix E.

The rationale behind comparing the selected baseline methods on deep learning–based registration is unclear.

We have revised the manuscript to improve its clarity. Specifically, on the long tail of modalities, we add comparisons to baselines that claim domain-agnostic generalization (SynthMorph, unigradICON, VFA) to appropriately compare with a domain-agnostic method like FireANTs. On the NLST and EMPIRE10 datasets, we choose baselines that already provide pretrained models for lung datasets, since models trained on brain datasets typically do not perform well on lung datasets. We have made this clearer in the revised manuscript.

Moreover, methods compared in Fig. 2(d–e) show accuracy only without reporting inference times. This weakens the argument that FireANTs is more efficient.

We only report accuracy as documented in the official challenge results. We do not have access to any annotated data for the validation sets used in these challenges, nor any of their evaluation code. Subsequently, we are only able to report runtimes for our method and other methods that have independently reported their runtimes (ANTs and DARTEL). To strengthen the argument that FireANTs is efficient, we have additionally performed a holistic comparison that is summarized in Figures 4 and 5(b).

The method assumes a unitary matrix U(x) in the polar decomposition of the Jacobian J(\phi)=U(x)P(x). This approximation removes all rotational components. While the authors justify that large rotations are rare, this is not universally true, e.g., in lung CT data, where airway and fissure rotations are significant. The impact of this approximation needs to be justified and well supported empirically.

A valuable addition would be an ablation study comparing Jacobian-free vs. Jacobian-based gradient directions.

We only assume that the Jacobian does not rotate the gradient vector by **more than 90 degrees** to use the Jacobian-free gradient as a “valid” descent direction. In practice, rotational components can be induced locally by transporting neighboring voxels in non-parallel directions, and the compositional updates ensure that large rotations can be formed by composing these small rotational effects from the gradients.

We study the impact of this approximation by ablating the effect of Jacobian-free optimization on performance and runtime over three datasets - OASIS (brain), NLST (lungs) and L2R-AbdomenMRCT (abdomen). These datasets represent three different organ systems with diverse morphological variations. Figure 5(d) shows that on all three datasets, the registration accuracy is not affected in any meaningful way while runtime increases significantly by adding the Jacobian term. Results in Figure 2(e) also show that FireANTs has the best fissure alignment among *all* participating methods, demonstrating that Jacobian-free optimization does not lead to catastrophic failure in real-world applications. Except for the ablation study, all our experiments employ FireANTs configured to use Jacobian-free descent directions.

Meanwhile, the manuscript would benefit from a theoretical analysis of the algorithm’s time complexity to support claims about scalability and efficiency.

We did not originally include a time complexity analysis because the number of optimization iterations can be variable and is specified by the user. We have added a holistic comparison in Section 2.5 and Figures 4 and 5(b) to support our claims about scalability and efficiency.

-In Fig. 2(b), the claim that FireANTs achieves zero percent singularity errors (compared to SVF or DARTEL) is questionable. Assuming all methods optimize the same objective with different

optimization schemes, a detailed explanation of why FireANTs avoids singularities while others do not is needed. Were regularization parameters identical? How were they selected?

We provide further theoretical justification regarding the numerical accuracy of diffeomorphisms and the limitations of velocity fields in general in Appendix E. Specifically, to ensure that numerical diffeomorphism is obtained, the gradient update **(id + \eta_t v_t)** needs to be numerically diffeomorphic. This happens only if the Lipschitz constant of \eta_t v_t is less than 1. We have added a proof (see Theorem 1 in Appendix E). In our FireANTs implementation, we make sure that **\eta_t = learning_rate / norm(v_t)** which is a lower bound for the Lipschitz constant of **v_t** (see Equation 16). This ensures that we always perform numerically diffeomorphic updates.

In the case of scaling-and-squaring, the base case **\varphi = id + v / 2^N** needs to be diffeomorphic (similar to the diffeomorphic update above). This requires choosing a value of scaling-and-squaring steps **N** such that the Lipschitz constant of **v / 2^N** is not more than 1. However, most implementations using stationary velocity field representations use a fixed value of **N** regardless of the Lipschitz constant of **v**, which leads to numerically non-diffeomorphic updates for DARTEL and other SVF methods. We add details in Appendix E.

These properties are purely a function of the behavior of velocity field **v** and are unrelated to regularization, which we see both mathematically and empirically in Appendix E.

In Fig.4, the hardware configurations used for baseline methods (e.g., ANTs, DARTEL) are not specified. Since FireANTs runs on GPU (A6000), comparing it to CPU-based baselines without clarification loses the fairness.

For the EMPIRE10 challenge, the baseline numbers are reported by the participating teams and they do not provide further details on the hardware used. We add comparative numbers on the CPU for ANTs and FireANTs for five brain datasets on identical CPU hardware for fair comparison.

The paper would benefit from a better organization, particularly in distinguishing prior work from the authors’ original contributions. Sections 4.3.1, 4.3.2, and much of the content in 4.4 (prior to 4.4.1) largely summarize established methods and should be moved to a dedicated background or related work section.

Our goal in constructing the structure of Section 4 was to provide the relevant context to the reader as to what design decisions were considered (Adam on the velocity field directly; using parallel transport for Riemannian gradient descent) before proposing adaptive optimization using the Eulerian differential that utilizes the group operation. SVFs were not used due to the computational overhead of scaling-and-squaring, their sensitivity of perturbations on the final result, and their dependence on integration steps being a function of the velocity field (more in Appendix E). Riemannian gradient descent was not used due to the requirement for computationally expensive parallel transport.

Contrary to Bigstream’s affine registration, the authors claim to preserve the in-frame/in-plane context during their affine registration step for the RnR mouse registration task. Please provide additional details on how the affine matrix is optimized.

We use an Affine Registration module (also part of the FireANTs framework for compositive transforms), with a multi-scale registration objective using the normalized cross correlation loss. The affine matrix is optimized using an Adam optimizer with a learning rate of 0.03, which is orders of magnitude larger than most deep networks and is possible due to the more stable dynamics of the low-dimensional representation of an affine matrix that is of interest here. All our scripts are included as part of the codebase.

Inconsistent use of symbols - η vs ϵ (update step size). Related references, for example, geodesic shooting methods are mentioned without proper citations. The manuscript would benefit from thorough proofreading and sentence refinement to improve its overall clarity and writing quality.

We thank the reviewer for these observations. We use η to denote a learning rate, and ϵ to denote an arbitrary (but small) step size. We have made the necessary clarifications in the manuscript as well as improved its readability.

Reviewer 3

Authors propose a new way to optimize for diffeomorphisms for image registration. The technique relies on the Eulerian differential as was discussed in Chapter 10 of Laruent Younes' book on Shapes and Diffeomorphisms. The main technical difference that I can see is that here authors assume that the Jacobian of the diffeomorphism, which is an expensive part to compute, to be positive definite. Based on this assumption, they define a Jacobian-free descent direction. Moreover, they use this in a momentum based optimizer and this leads to tremendous speed ups in the minimization. Authors show results for various datasets.

Strengths:
1. The results this article reports are extremely impressive. Authors indeed solve very challenging registration problems in lightning speeds. Furthermore, based on the metrics they show, the resulting deformation field remains a diffeomorphism.
2. The amount of experimentation in this article is very impressive. Authors thoroughly assessed their methods and compared with state-of-the-art techniques. This is indeed an excellent experimental design and section overall.
3. The technical descriptions of alternative minimization approaches are extremely well done. I believe the method section until 4.4 is really concise yet informative.
4. The proposed method seems sound but I have some questions that I will pose later on.
5. Overall I think this is a great article and with better descriptions of the actual method, it can really shine and would have a significant impact on the field.

We very much appreciate the Reviewer’s positive assessment of both the strengths of our methods and our extensive experimental work.

It seems to me that the technique is largely motivated from Younes' book - Sections 10.2 and 10.4. I believe this similarity should be further discussed and the developments here can be linked to the method described therein.

While Younes’s work has indeed been deeply influential to our thinking, we only use the definition of the Eulerian differential from his book to draw a connection with the Gateaux derivative that is used in autodifferentiation pipelines. The rest of the technical contributions are novel to our knowledge. Even our definition of the Eulerian gradient deviates significantly from

the one used in Younes et al. We discuss the justification behind the alternate formulation in Section 4.4.2. Specifically, we use the pushforward image instead of the pullback image to compute the gradient with respect to the warp **\varphi** directly instead of storing its inverse. Similarly, the order in which the infinitesimal warp is composed in our paper (Equation 7) is opposite to that in Younes et al. In the latter formulation, the subsequent derivation would provide us an expression for **V(y) = v(\varphi(x))**. This quantity needs to be brought back to the Eulerian frame to compute adaptive estimates, requiring computation of **v(x) = V(\varphi^{-1}(y))** which requires the inverse. Our design decision avoids storing the inverse warp and computing the pullback of the gradient for optimization, leading to a more efficient implementation.

The implication of the main assumption, which states that the Jacobian of the transformation is positive definite, should be discussed and analyzed.

Since the transformation is diffeomorphic in nature, the Jacobian of the transformation is always positive definite by definition.

It is unclear to me why the momentum and EMA of the squared gradients can be computed without any special treatment. If Eulerian differential is indeed a Riemannian gradient, then how come the state variables can be used as they are without requiring a parallel transport?

This is because diffeomorphisms are also a Lie group, but a general Riemannian manifold is not. Specifically, the Eulerian gradient cannot be defined for a Riemannian manifold that is not a Lie group, since the **\circ** operator is meaningless for a general Riemannian manifold. We leverage this useful property to define gradients in the tangent space at identity, which makes adaptive estimates easy to compute without special treatment. Technically, other Lie groups like affine or rotational transformations can also use this formulation after defining the Eulerian differential appropriately. However, manifolds considered in [85] like Sphere, Steifel, Perspective Projective, and Lorentz manifolds are not groups, and thus a Eulerian differential cannot be defined. Therefore, optimization on these manifolds has to be performed with Riemannian gradients.

The only other special treatment for diffeomorphisms (or any other Lie group) would be to multiply the Gateaux derivative with the Jacobian of the transformation to compute the correct gradient descent direction for minimizing the Eulerian differential, but we empirically drop the term and see significant speedup with no penalty in performance (Figure 5(d)).

The description of the method starting from 4.4 may require a bit more clarifications.

While we believe the description is comprehensive and does not skip over any important steps, we welcome the opportunity to elaborate on any aspect that may be confusing to the Reviewer.

I believe the term ϕ_{0t}^v is not defined in the text.

The term is mentioned in Section 4.4 in the “Adaptive optimization on diffeomorphisms” paragraph **- \varphi_{0t}^v = \exp_{id}(tv),** but it is first defined in the beginning of the section. We have now moved the definition so that it comes after Equation 7.

The Eulerian differential is denoted as $\partial \bar{U}$ but can it be $\bar{\partial} U$? The definition of the Eulerian differential seems to be the same in between [61] and here, but perhaps how it is used may be different.

We have indeed changed the Eulerian differential to be **$\bar{\partial} U$**
The definition of Eulerian differential in our work is different than [61], and has been specifically formulated to be compatible with the group action of \varphi on the image as $**(I \circ \varphi)(x) = I(\varphi(x))$** instead of the group action $**(I \circ \varphi)(x) = I(\varphi^{-1}(x))$** used in [61]. We developed this alternative definition to avoid storing and updating $**\varphi^{-1}**$, and to permit obtaining the Gateaux derivative of φ directly using PyTorch. We have added a discussion in Section 4.4.2 that details our alternative formulation for the Eulerian differential, as well as the computational and convenience bases for this development.

Reviewer 4

This paper proposes a new registration method, named FireANTs. Technically, it mainly claims two contributions. First, it uses the Eulerian differential to get the descent directions, which can be efficiently computed through GPU based libraries by means of the Gateaux derivatives, leveraging the property that the diffeomorphism is also a Lie group. Second, it validated the proposed method in many datasets and shown that the proposed method is orders of magnitude faster than the SOTA methods. This improved efficiency therefore enables the dense registration across high resolution images (like sub-micron mouse isocortex volumes), which are prohibitively slow when using the traditional method. So, the proposed method can potentially extend the registration application range for a wider imaging field. The paper is well written and organized. The self-inclusion of the paper is good, and the method is clearly elaborated with

rigorous formulations. The validation of the experiments shows substantial improvement in computation efficiency.

We are appreciative that the Reviewer identifies exactly the core of the mathematical contribution of the paper. We are further encouraged that they recognize the improved efficiency and wider application range of our method across various biomedical and life science domains.

However, there is one important question that is not explicitly clarified. Is the efficiency improvement contributed to the GPU or the method itself. Because the computation of Gateaux is also expensive. For the comparison baseline methods, like ANTs and DARTEL, are they using the traditional CPU based version or their optimizations are also updated with the GPU version. If these baseline methods are using CPU version, then the comparison may not be fair, and the massive efficiency improvement cannot be only credited to the proposed framework.

We agree with the Reviewer that further clarification would be helpful as the efficiency gained is accrued across multiple novel developments: 1) a GPU algorithm; 2) a more efficient CPU implementation; and 3) a better optimizer that is expected to converge in fewer iterations - these three factors can be tested independently. Our revised manuscript examines each of the benefits independently by (a) comparing runtimes of CPU implementations of ANTs and FireANTs; (b) evaluating the improvements over a CPU implementation with a GPU implementation; and (c) comparing the runtime and memory efficiency compared to state-of-the-art deep learning methods.

To substantiate the claims on CPU implementation, we compare FireANTs with ANTs on CPUs on five brain MRI datasets as summarized in Figure 4(a). This shows a significant improvement in speedup with identical compute budget on multi-threaded CPU (which is now attributed to both efficient implementation and faster convergence - since we actually perform more computation due to the need to update the Adam optimization state in each iteration, and to compute the modified gradient). Figures 4(a) and 4(b) also show performance gains on GPUs, which demonstrate that this efficiency can be improved further by having a GPU implementation. Figure 4(c) shows that our method is highly competitive on runtime and up to 10x more memory efficient compared to deep learning methods on a GPU, opening its scalable application to much larger images than can be practically processed by the current state-of-the-art. Figure 5(b) shows that FireANTs also makes possible the routine conduct of hyperparameter studies by requiring significantly fewer resources than amortized hyperparameter learning methods.

In Figure 4(b), the runtimes of ANTs and DARTEL are provided by the participants in their challenge submission writeup. Since there is no reproducibility statement including the hardware used to report these numbers, we are unable to independently reproduce these runtimes and have, therefore, directly used the published numbers in the challenge writeup as a benchmark / baseline. However, the FireANTs speedups are consistent with those observed in Figure 4(a).

Also, besides the computation cost, some discussion on the computation complexity of the proposed method, ANTs, and DARTEL can be meaningful to clarify the efficiency originations.

We now include a discussion on the additional computational overhead incurred by optimizing a stationary velocity field (DARTEL) instead of optimizing the diffeomorphism on the Lie group directly (ANTs, FireANTs) in Appendix E.

The self-inclusion of the paper is quite good in most sections. However, a) in section 4.1.1, “In this work, we only consider a subgroup of diffeomorphisms”. It would be more comprehensive to point out the ones that “out of” the subgroup of diffeomorphisms. This would enlarge the whole view for the readers to follow the registration problem.

. b) since one contribution of the paper is the rigorous mathematic framework of the adaptive optimization in this compact supported subgroup of diffeomorphism. However, the essential motivation (from the application view, a large deformation can be decomposed into several small deformations?) of focusing on this subgroup of diffeomorphism is not explicitly discussed.

The paper addresses the condition number’s effect on the optimization, which is related to the cost function of the registration. The logic seems to be, even with diagonal Hessian (either in L2 registration loss or the handcrafted example in A.3), the ill-conditioning of the registration can substantially slow down the optimization. So, adaptive optimization is preferred. However, this is more related to the choice of the optimization strategy, not related to the motivation of proposing the Eulerian differential to get the descent direction. Because the comparison baselines can also use the adaptive optimizations.

We, again, agree with the Reviewer! We require an adaptive strategy for optimizing diffeomorphisms to mitigate ill conditioning, but that requires us to account for both the optimizer state updates and gradient updates to be diffeomorphic. We walk through the design choices we considered: specifically, Section 4.3.1. considers using Adam on the Lie algebra of stationary velocity fields directly, but discards this approach due to limitations discussed in Appendix E; Section 4.3.2 discusses the computational overhead associated with Riemannian gradient descent, which motivates a group-theoretic formulation instead, and our Jacobian-free approximation to further bring down computational overhead without sacrificing accuracy.

The key idea is to establish a connection between the Eulerian differential, that defines rate of change with respect to infinitesimal “diffeomorphic changes” (which is what we care about), and the Gateaux differential (regular derivative). This allows us to make diffeomorphic updates while having access to only the Gateaux differential (via PyTorch), and to define the Eulerian differential in a way that situates the gradient v in the tangent space at identity, making Adam updates trivial.

The ANTs atlas construction not only aligns the shape but also aligns the intensity appearance. Is this the reason that in Fig. 6, the FireANTs registration looks not as “bright” as ANTs?

We trace these differences back to a few subtle but key steps in the template generation process: (a) the implementations and chosen hyperparameters of the unsharp mask; and (b) inverse shape update equations. Particularly, the “saturated” appearance in FireANTs is very likely due to a different Laplacian update, and winsorizing the FireANTs template appropriately “recovers” the brightness. However, a less “bright” image preview does not imply a lower quality or inferior template. This is shown both quantitatively and qualitatively, since atlases generated by FireANTs have more high frequency details and have better Dice label map alignment.

It is confusing to understand “Fig. 8 shows that across all resolutions, more than 60% of foreground pixels have a condition number greater than 10”.

For background voxels, the gradients are zero, and their Hessians are flat. These voxels do not contribute to the ill conditioning of the problem. Therefore, we consider only foreground voxels.

In the comparison experiment, it is not clear whether the validation used the Jacobian-Free Eulerian descent or Jacobian-involved Eulerian descent. Therefore, the immediate question is, are there any big differences between them? What are the Pros and Cons? Some discussions can be helpful to clarify this question.

We now clarify that Jacobian-free Eulerian descent is used in all our experiments in the paper. In response to Reviewer’s question, we have included an ablation on the effect of adding the Jacobian term on both runtime and accuracy over three datasets - OASIS, NLST, and L2R-Abdomen MRCT (see Figure 5(d)). This experiment shows that our approximation does not significantly reduce accuracy, but makes the method significantly faster across various anatomical structures and configurations.

Two minor typos:
In sec. 2.2, “for deep methods”->”for deep learning methods”.
In sec. 4.3.1, “However, there a number of challenges”-> “However, there are a number of challenges”.

We thank the Reviewer for pointing out these typos. We have made these corrections.

In summarize, this is a good self-included registration paper with well-validated effective registration improvement in practical. However, given the above comments, especially the non-clarified origin of efficiency, therefore, if the paper can further convince that the efficiency improvement is indeed from the introduction of the framework, not from the GPU involved computation, the technique contribution would be substantial. The reviewer would be in favor of the publication. Otherwise, the reviewer feels that this paper may be more suitable for a specific journal focused on computer vision or image processing.

We thank the Reviewer, once again, for their constructive feedback and hope we have adequately addressed the multifactorial origin of the method’s efficiency by adding comparisons

on both CPUs and GPUs, and showing significant improvement in runtime across six datasets. The flexibility of FireANTs to run both on CPUs and GPUs provides another dimension of adaptability for researchers regardless of the availability of GPUs on the specific hardware that they utilize, ensuring that they can run and port the same algorithm under the hood .

Nature Communications manuscript NCOMMS-25-25699B

We thank the reviewers and editors for their thoughtful evaluations and positive assessment of our revised manuscript. Below we address the remaining minor points raised in this round of reviews.

Reviewer #1

Remarks to the Author:

The authors have provided substantial improvements to the article and their answers to my questions are relevant. I would recommend this article for publication in Nature Communication.

We appreciate the reviewer's positive feedback and glad we were able to address all concerns.

Reviewer #2

Remarks to the Author:

Thank you for the authors' thorough responses to my comments. I have no further concerns and am happy to recommend acceptance of the paper.

We appreciate the reviewer's positive feedback and glad we were able to address all concerns.

Reviewer #3

Remarks to the Author:

Authors have answered my concerns and questions.
With the modifications, clarified explanations and the additional results, I believe the article is in a good state and it will surely be an important contribution to the field of biomedical imaging for a diverse set of applications.

We appreciate the reviewer's positive feedback and glad we were able to address all concerns.

Reviewer #4

Remarks to the Author:

The authors did a great job addressing my issues. I do not have any questions.

We appreciate the reviewer's positive feedback and glad we were able to address all concerns.
